# Dysregulated AKT signaling reprograms osteosarcoma to drive selective reliance on EP300

**DOI:** 10.1084/jem.20251898

**Published:** 2026-10-20

**Authors:** Ian M. Delahunty, Stephanie Nance, Yang Zhang, Francois Lamoureux, Qi Liu, William C. Wright, K. Elaine Ritter, Noha A.M. Shendy, Bénédicte Brounais-Le Roye, Sandra J. Kietlinska, Mohammad A. Mohammad Nezhady, Yuan Feng, Barbara De Kegel, Matthew G. Rees, Mustafa Kocak, Ashish B. George, Meifen Lu, Anoop M. Kavirayani, Bo Wang, Yanling Liu, Paris E.A. Prinsen, Yousef Khashana, Vernon Justice Ebegboni, Melissa M. Ronan, Jennifer A. Roth, E. Alejandro Sweet-Cordero, Lillian M. Guenther, Paul Geeleher, Xiaotu Ma, Benjamin Ory, Jun Qi, Brian J. Abraham, Adam D. Durbin

**Affiliations:** 1 Division of Molecular Oncology, Department of Oncology, St. Jude Children’s Research Hospital, Memphis, TN, USA; 2 Department of Computational Biology, St. Jude Children’s Research Hospital, Memphis, TN, USA; 3 Nantes Université, INSERM UMR 1307, CNRS UMR 6075, CRCI2NA, Nantes, France; 4 Department of Cancer Biology, Dana-Farber Cancer Institute, Department of Medicine, Harvard Medical School, Boston, MA, USA; 5 St. Jude Graduate School of Biomedical Sciences, St. Jude Children’s Research Hospital, Memphis, TN, USA; 6 Broad Institute of MIT and Harvard, Cambridge, MA, USA; 7 Department of Pathology, St. Jude Children’s Research Hospital, Memphis, TN, USA; 8 Division of Pediatric Oncology, Department of Pediatrics, University of California San Francisco, San Francisco, CA, USA

## Abstract

Cancers depend on transcriptional control to maintain the malignant cell state. EP300 and CBP are paralogous master epigenetic enzymes that control normal and malignant transcription. Using a cancer-wide integrative chemical-genetic analysis, we demonstrate enhanced dependency on EP300, compared with CBP, in cell lines from most cancer lineages, including lethal osteosarcoma (OS). Using a selective EP300 degrader (JQAD1), we identify that a genetically defined subgroup of OS with dysregulation of the PI3K–AKT–mTOR pathway is genetically and chemically sensitive to loss of EP300. Activated AKT promotes sensitivity to JQAD1, driven by relocalization of EP300, CBP and H3K27ac to genetic subtype-enriched dependency loci. AKT inhibition combines with JQAD1 to synergistically suppress the growth of AKT-dysregulated OS cells in vitro and in orthotopic xenografts. These observations extend across >850 cancer cell lines, where synergy is predicted by elevated protein synthetic rates. These findings reveal determinants of EP300 degrader function in OS and provide a foundation for co-targeting EP300 and AKT in cancers with enhanced protein translation.

## Teaser

Here, Delahunty et al. identify that EP300 controls the growth of AKT-dysregulated osteosarcoma cells by regulating expression of state-specific genetic dependencies. AKT inhibitors synergize with EP300 degraders to suppress osteosarcoma growth in vitro and in vivo. These agents positively combine across cancer cells in a manner dependent on control of protein synthesis.

## Introduction

Gene expression is controlled by interactions of epigenetic enzymes and transcription factors, which establish and maintain gene promoter, enhancer, repressor, and insulator elements (Kwak et al., 2013; Margueron and Reinberg, 2010; Tie et al., 2009; Wang et al., 2009; Whyte et al., 2013). Driven by the activity of epigenetic enzymes, promoter and enhancer elements are either activated or repressed, leading to alterations in gene expression and the resultant malignant phenotype (Dawson and Kouzarides, 2012; Dawson et al., 2012; Jenuwein and Allis, 2001; Li and Seto, 2016). In cancer cells, these enzymes may be co-opted, driving aberrant activation or repression of gene expression and resulting in malignant phenotypes such as uncontrolled proliferation, repressed differentiation, or migratory behaviors (Wimalasena et al., 2020). How the activity of these enzymes is shaped by mutated or activated oncogenes to result in dysregulated transcription is an area of active interest. Further, since cancer cells broadly rely on epigenetic enzymes to control the malignant transcriptional program, and these enzymes may contain catalytic or non-catalytic binding pockets suitable for targeting with small molecules, there has been extensive interest in pharmacologic development and targeting of these proteins (Liu et al., 2024; Wimalasena et al., 2020; Zhou and Cole, 2025).

Two central proteins involved in the control of gene expression are EP300 and CBP (Arany et al., 1994; Ogryzko et al., 1996). EP300 and CBP (*KAT3B* and *KAT3A*) are paralogous lysine acetyltransferases that establish acetylation marks across the proteome, including acetylation of histone H3, lysine-27 (H3K27ac), a mark of active enhancer and promoter elements (Creyghton et al., 2010; Weinert et al., 2018; Zhou and Cole, 2025). These proteins are commonly co-expressed in cancer cells (Durbin et al., 2022); however, some cancer cells display enhanced reliance on one of these proteins due to mechanisms including selective recruitment by lineage-specific transcription factors (Durbin et al., 2022) or mutations in the other (Ogiwara et al., 2016). Due to their key role in promoting malignancy, medicinal chemistry efforts have led to small-molecule histone acetyltransferase (HAT), bromodomain (BRD), or KIX-domain inhibitors that target both of these proteins (Joy et al., 2021; Lasko et al., 2017; Liu et al., 2024; Ramaswamy et al., 2018; Shendy et al., 2024; Welti et al., 2021; Zhou and Cole, 2025). However, the high amino acid sequence homology between EP300 and CBP in these targetable domains hinders selective targeting, since available small-molecule inhibitors targeting EP300 typically also target CBP. These properties may lead to undesirable toxicities in untransformed cells, thereby reducing a potential therapeutic window. To take advantage of the enhanced reliance of individual tumor types on either EP300 or CBP, recently, we and others reported first-generation proteolysis-targeted chimeras (PROTACs), including JQAD1 and others, that selectively target EP300 for proteasomal degradation, with minimal effects on CBP (Chen et al., 2024; Durbin et al., 2022; Marsh et al., 2025). These compounds are well tolerated in vivo, likely due to the retention of CBP partially compensating for the loss of EP300 in untransformed cells (Durbin et al., 2022). These PROTACs, which drive proximity-based ubiquitination and proteasomal degradation of EP300 by bridging to the E3 ubiquitin ligase receptor cereblon (CRBN), are dependent on expression of *CRBN* (Durbin et al., 2022; Lee et al., 2023). However, key additional questions remain, including what mechanisms control compound efficacy and which tumor lineages may be optimal to selectively target EP300 or CBP.

Thus, here, we integrate functional genomics and chemical biology analyses to identify cell lines representing cancer lineages with enhanced reliance on EP300, compared with CBP, that are also more sensitive to the EP300-targeted PROTAC, JQAD1. We identify that EP300 is generally more required than CBP for growth of cancer cells across 53 cancer lineages. Osteosarcoma (OS), an aggressive bone sarcoma with no available targeted agents, is sensitive to genetic loss of EP300. A genetic subtype of OS, marked by dysregulation of the AKT signaling pathway, is specifically sensitive to JQAD1. We demonstrate that ectopic AKT activity drives sensitivity to JQAD1 in OS cells by enforcing genome-wide relocalization of EP300, CBP, and H3K27ac to drive the expression of genes required for the growth of AKT-dysregulated OS. To enhance the effects of single-compound targeting with JQAD1, we demonstrate that combinations of AKT inhibitors and JQAD1 synergistically suppress the growth of AKT-dysregulated OS cells in vitro and in orthotopic murine models in vivo. Testing this model across >850 cancer cell lines, we observe frequent additive to synergistic interactions between AKT inhibitors and JQAD1, related to AKT inhibitor–mediated disruption of protein synthesis. These observations provide a foundation for consideration of combination therapies targeting EP300 and AKT across multiple cancer lineages marked by elevated AKT signaling and EP300 dependency and identify a genetically defined subtype of OS with exceptional responses to combination treatment.

## Results

### EP300 is an enriched dependency and selectively required in OS

Targeting one of a paralogous, co-expressed set of proteins may enhance a therapeutic window by sparing the other paralog in untransformed cells. This strategy is challenging using small-molecule inhibitors with nonspecific activity against closely related paralogs. Previously, we developed the small-molecule PROTAC degrader of EP300, JQAD1, and demonstrated it had single-agent efficacy in pediatric neuroblastoma (Durbin et al., 2022). JQAD1 is well tolerated in vivo with broad anticancer effects across different cancer cell lines (Durbin et al., 2022). Thus, we sought to understand if other lineages in addition to neuroblastoma were candidates for selective targeting of EP300.

**Figure 1.**
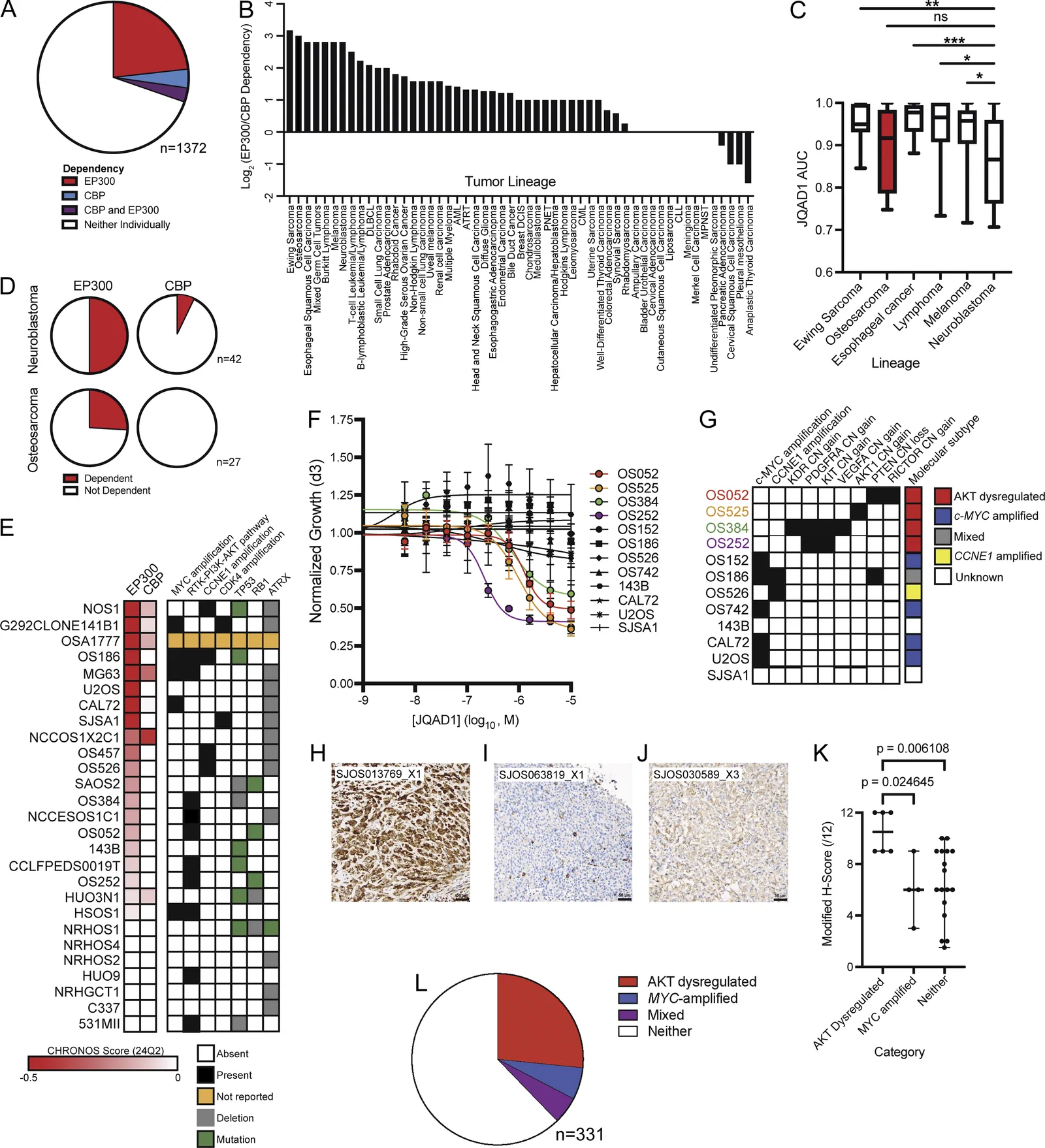
**EP300 is a selective dependency in AKT-dysregulated OS. (A)** EP300 and CBP dependency scores were extracted from the 1,372 cell lines in the 24Q2 release of DepMap. CHRONOS Score < −0.5 was used to identify dependency. **(B)** Relative log_2_ ratios of dependency on either EP300 or CBP across tumor lineages. Annotations from DepMap; sample numbers detailed in the Materials and methods. **(C)** JQAD1 AUC by PRISM screening across the top six lineages enriched for EP300, compared with CBP dependency in B. Data from Durbin et al. (2022). Box-and-whisker plots illustrate the median (wide line), quartiles (box), and 1.5*interquartile range (whiskers). *P < 0.05, **P < 0.01, ***P < 0.001 by Students' *t* test. **(D)** Dependency on EP300 and CBP plotted in OS (*n* = 27) and neuroblastoma (*n* = 42) cell lines, using CHRONOS Score < −0.5. **(E)** Dependency plot of OS cell lines in DepMap 24Q2 release, denoting CHRONOS Score and tumor genetics from the CCLE. RTK–PI3K–AKT pathway dysregulation noted by either copy number loss or mutation of *PTEN*, or gain/activating mutations of RTKs, *PIK3CA*, or *AKT1-3*, as detailed in the Materials and methods. **(F)** CellTiter-Glo analysis of OS cell lines treated with JQAD1, after 3 days of treatment. Bars = SD, *n* = 3 independent replicates. **(G)** Genetics of OS PDX lines in F, classifying each into a molecular subtype. **(H–J)** Representative IHC to phospho-Ser-473 AKT performed on OS PDX samples with genomic lesions defining them as AKT dysregulated (H), *c-MYC* amplified (I), or no known lesion in AKT or MYC pathways (“neither”) (J). Examples are reproduced from Fig. S1 for comparison. **(K)** Quantitation of histology scores of OS PDXs for phospho-Ser-473 AKT IHC in AKT dysregulated (*n* = 6), *MYC* amplified (*n* = 4), or no lesion in these pathways (neither) (*n* = 17). Bars represent data spread, with horizontal lines indicating the median value. Y axis represents H-scoring of phospho-Ser-473 AKT, as in the Materials and methods. P values were determined by two-way ANOVA with a post hoc Tukey test. **(L)** Distribution of 331 OS samples from the AACR GENIE version 19.0 public cohort. Identified are samples with mutations, CNVs or structural variants indicating status as AKT dysregulated, *MYC* amplified, mixed, or neither. Categories are determined as detailed in the Materials and methods*.*

**Figure S1.**
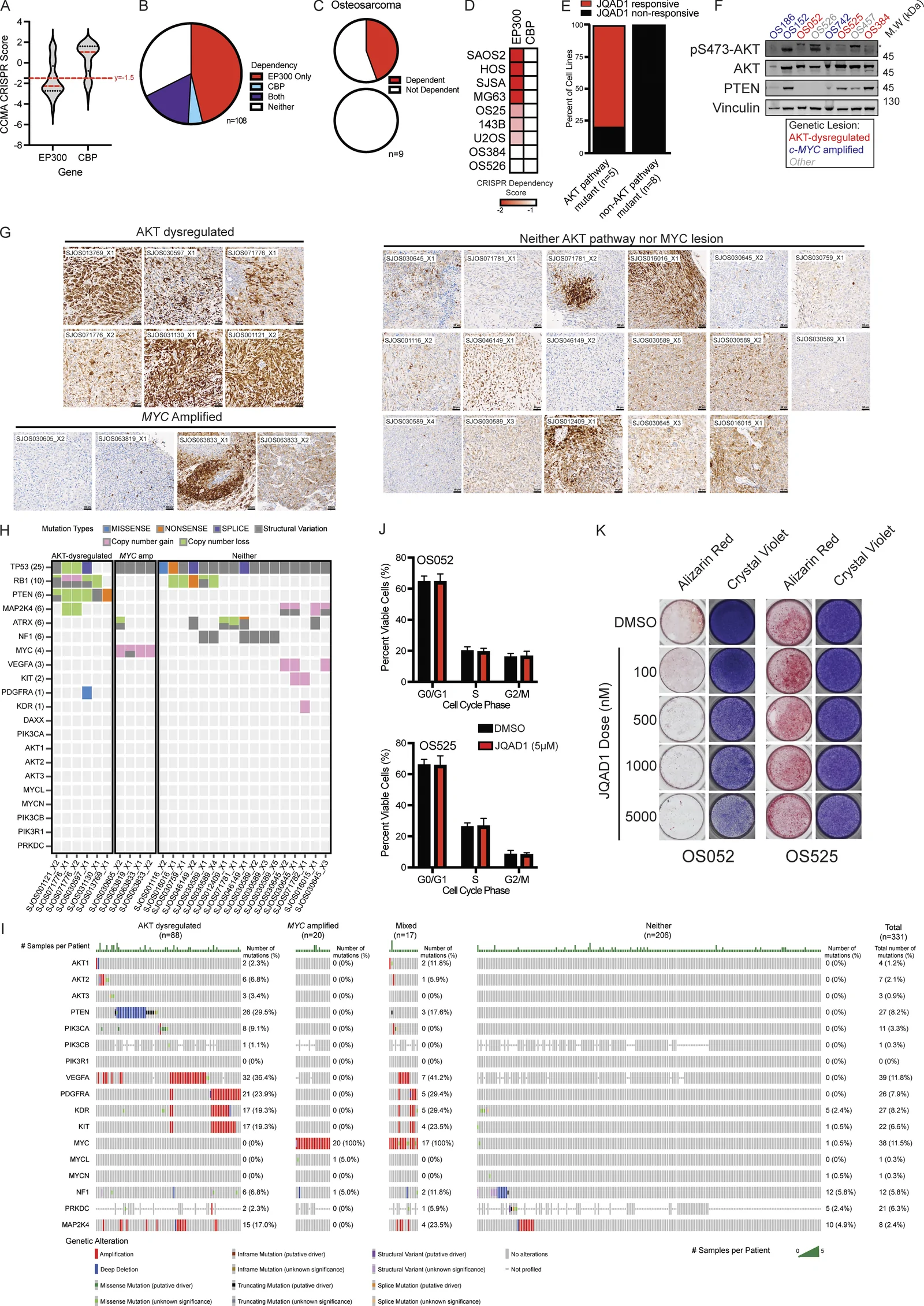
**EP300 is a selective dependency in a genetically defined subset of OS. (A) **CRISPR score of EP300 and CBP dependency across *n* = 108 cell lines from the CCMA. Dependency cutoff is CRISPR Score < −1.5. **(B)** Pie chart representation of all cell lines in CCMA noting fractions dependent on EP300 (50/108), CBP (5/108), both (18/108), or neither (35/108). **(C)** Pie chart representation of OS cell lines in CCMA (*n* = 9), noting fractions dependent on EP300 (4/9) or CBP (0/9). **(D)** Heatmap of dependency in OS cell lines in CCMA on EP300 or CBP. **(E)** Stacked bar plot of cell lines tested for JQAD1 sensitivity and their genetic status for lesions causing AKT dysregulation. Red = JQAD1 sensitive, black = JQAD1-insensitive. *n* = 5 (AKT dysregulated), *n* = 8 (AKT non-dysregulated). **(F)** Western blot of OS PDX cell lines for noted proteins for comparison. Data are representative of three independent lysates and blots. AKT-dysregulated PDX cell lines are highlighted in red. M.W, molecular weight. **(G)** IHC to phospho-Ser-473-AKT was performed on OS PDX samples with genomic lesions defining them as AKT-dysregulated (*n* = 6), *c-MYC*–amplified (*n* = 4), or no known lesion in AKT or MYC pathways (“neither”) (*n* = 17). Selected examples are also reproduced in Fig. 1. **(H)** Heatmap of gene mutations from *n* = 27 OS PDXs from G. Mutations profiled are those commonly found in OS, as detailed in the Materials and methods*.***(I)** Panel-based tumor sequencing from *n* = 331 OS patients obtained from the AACR GENIE version 19.0 cohort. Demonstrated are patients with mutational, copy number, and structural variant data. Samples were stratified by grouping: AKT dysregulated (*n* = 88), *MYC* amplified (*n* = 20), mixed (*n* = 17), or neither (*n* = 206). Percentages of variants or mutations are noted for each gene in each grouping, and overall in the cohort. **(J)** Cell cycle analysis of viable OS052 and OS525 cells after 48 h of treatment with DMSO or JQAD1 (5 μM). Bars = SD, *n* = 3 independent biological replicates. **(K)** OS052 and OS525 cells were cultured for 18 days in the presence of OB differentiation medium and treated with DMSO or a dose response of JQAD1. Mineralization was revealed after Alizarin red staining at the end of the culture. Cell viability was evaluated by crystal violet staining after 10 days of treatment with DMSO or a dose response of JQAD1. Data are representative of *n* = 3 independent replicates per dose and cell line. Source data are available for this figure: SourceData FS1.

We examined data from the Cancer Dependency Map (24Q2 release), to establish the effects of genetic loss of EP300 and CBP across all tumor cell lines (Fig. 1 A, CHRONOS Score < −0.5). Across 1,372 cell lines, genetic disruption of EP300 caused loss of fitness in 314 (23%) and disruption of CBP caused loss of fitness in 61 (4%). Genetic disruption of either EP300 or CBP caused loss of fitness in 45 cell lines (3%) (Fig. 1 A). To confirm these results, we examined similar data from a separate dataset, the Childhood Cancer Model Atlas (CCMA), which demonstrated again that EP300 was an enriched genetic dependency, compared with CBP (Fig. S1, A and B).

Next, we used these data to establish whether specific lineages more strongly required EP300 over CBP. Organizing cells by tumor cell lineage and ranking by the ratio of genetic dependency on EP300 versus CBP, we found that most lineages displayed enhanced genetic dependency on EP300, compared with CBP. Few lineages, such as anaplastic thyroid carcinoma, mesothelioma, cervical squamous cell carcinoma, and pancreatic adenocarcinoma, displayed enhanced reliance on CBP compared with EP300 (Fig. 1 B). As previously (Durbin et al., 2022; Lee et al., 2023), neuroblastoma showed enhanced reliance on EP300 compared with CBP. However, other lineages displayed even greater relative dependency on EP300 (Fig. 1 B). To identify lineages potentially susceptible to pharmacologic disruption using the EP300-targeted protein degrader, JQAD1, we reanalyzed our prior screening data, in which JQAD1 was tested across 544 cancer cell lines (Durbin et al., 2022). Of the top six lineages nominated by DepMap analysis, JQAD1 frequently reduced cell growth in OS cells, comparably with neuroblastoma (Fig. 1 C and Tables S1 and S2). While only 7/27 (25.9%) of OS cell lines were genetically dependent on EP300 using a stringent CHRONOS Score cutoff of < −0.5, none were genetically dependent on CBP (Fig. 1 D). In comparison, while 20/42 (47.6%) of neuroblastoma cell lines were genetically dependent on EP300, these cell lines were also occasionally dependent on CBP (2/41 = 4.9%) (Fig. 1 D). Similarly, in the CCMA dataset, EP300 was a genetic dependency in 4/9 OS cell lines, and no cells were dependent on CBP (Fig. S1, C and D). Thus, a proportion of OS cell lines are both highly dependent on EP300 but not CBP and are susceptible to JQAD1 treatment.

OS is a genetically disordered tumor with rapid tumor evolution and unstable genetics (Espejo Valle-Inclan et al., 2025; Meltzer and Helman, 2021). EP300 is implicated in OS, functioning to reorganize enhancer elements that drive pro-metastatic gene expression (Morrow et al., 2018). OS genomes often bear mutations in *TP53* and *RB1*, amplifications of c-*MYC*, *CDK4*, *CCNE1*, and *AKT1*, and loss of *PTEN* and *ATRX* (Chen et al., 2014; Kovac et al., 2015; Sayles et al., 2019). These genetic lesions may have prognostic significance; for example, loss of *PTEN* and amplification of c-*MYC* have each been associated with metastatic disease and poorer overall survival (Feng et al., 2020; Gong et al., 2017; Marinoff et al., 2023; Zhou et al., 2019). Contributing to the dismal survival rates in metastatic/relapsed OS patients are limited advancements in targeted drug development. Thus, we next sought to determine if there were correlations between dependency on common genetic aberrations in OS and EP300 dependency. By correlating genomic events found in OS in the Cancer Cell Line Encyclopedia (CCLE) with DepMap dependency data (*n* = 27 cell lines), we did not observe any single-gene mutational patterns predisposing to EP300 dependency (Fig. 1 E). We did, however, observe that several EP300-dependent lines bore mutations in critical multi-gene pathways. In addition to deletion of *PTEN*, OS cell lines, as well as primary tumors and patient-derived xenografts (PDXs) (Perry et al., 2014; Sayles et al., 2019; Schott et al., 2024), often display copy number gain of upstream growth factors and receptors such as *VEGFA*, *KDR*, *PDGFRA*, and *KIT*, in addition to gains of *PIK3CA*, *AKT1*, or *RICTOR* and loss of *PTEN*. Since these mutations are components of a classical receptor tyrosine kinase (RTK)–PI3K–AKT–mTOR pathway (Vasan and Cantley, 2022), we termed tumors bearing these mutations “AKT-dysregulated” OS.

Next, we validated the effects of EP300 degradation in OS cell lines. In addition to conventional OS cell lines, to limit the potential confounding effects of genomically unstable cell lines in culture, we used a recently reported group of low-passage OS cell lines derived from PDXs (Sayles et al., 2019; Schott et al., 2024). JQAD1 elicited dose-dependent growth inhibition in four cell lines: one conventional (OS252) and three low-passage PDX cell lines: OS052, OS525, and OS384 (Fig. 1 F). Each of these harbored mutations consistent with AKT dysregulation, including multiple RTK copy number gains (OS384, OS252), *RICTOR* gains and *PTEN* loss (OS052), and amplification of *AKT1* (OS525) (Fig. 1 G). In contrast, JQAD1-resistant cells (OS152, OS186, OS526, OS742, 143B, CAL72, U2OS, and SJSA1) harbored other genomic events, including amplification of c-*MYC* or *CCNE1* (Fig. 1 G).

To confirm that these genetic lesions resulted in dysregulated AKT activity, we performed western blotting of PDX-derived cells for activated AKT (phosphorylated Ser-473), in addition to total AKT and PTEN proteins. PhosphoSer-473-AKT was elevated in cell lines with predicted AKT dysregulation (Fig. S1 F). Next, we examined tissue sections from 27 human low-passage OS PDXs with matched whole-genome sequencing (WGS) and RNA sequencing (RNA-seq) data. WGS analysis of known AKT pathway members, along with *c-MYC*/*MYCN*/*MYCL* and other genes commonly mutated in OS, identified samples with isolated AKT pathway mutations (AKT dysregulated, *n* = 6/27), *c-MYC*/*MYCN*/*MYCL* gene amplifications (“MYC amplified,” *n* = 4/27), or no mutations in these genes (*n* = 17/27) (Table S3 and Fig. S1, G and H). Immunohistochemistry (IHC) demonstrated statistically significantly increased phospho-Ser473-AKT in predicted AKT-dysregulated PDXs, compared with other groups (Fig. 1, H–K; and Fig. S1, G and H). To identify if similar changes were also found in human samples, we examined sequencing panel-based data from 331 patients with OS, derived from the AACR GENIE database version 19.0 public cohort (AACR Project GENIE Consortium, 2017). AKT dysregulation mutations were found in 88/331 patients, which was similar to the proportion identified in the PDX models (Fig. 1 L and Fig. S1 I). These data suggest that mutations predicted to promote activation of the AKT pathway (“AKT dysregulation”) are associated with activation of the pathway in cell lines and PDXs in vivo and similarly found in human primary tumors. Further, these data indicate that activation of this axis may be correlated with JQAD1 activity in OS.

### AKT dysregulation drives sensitivity to the targeted EP300 degrader JQAD1

**Figure 2.**
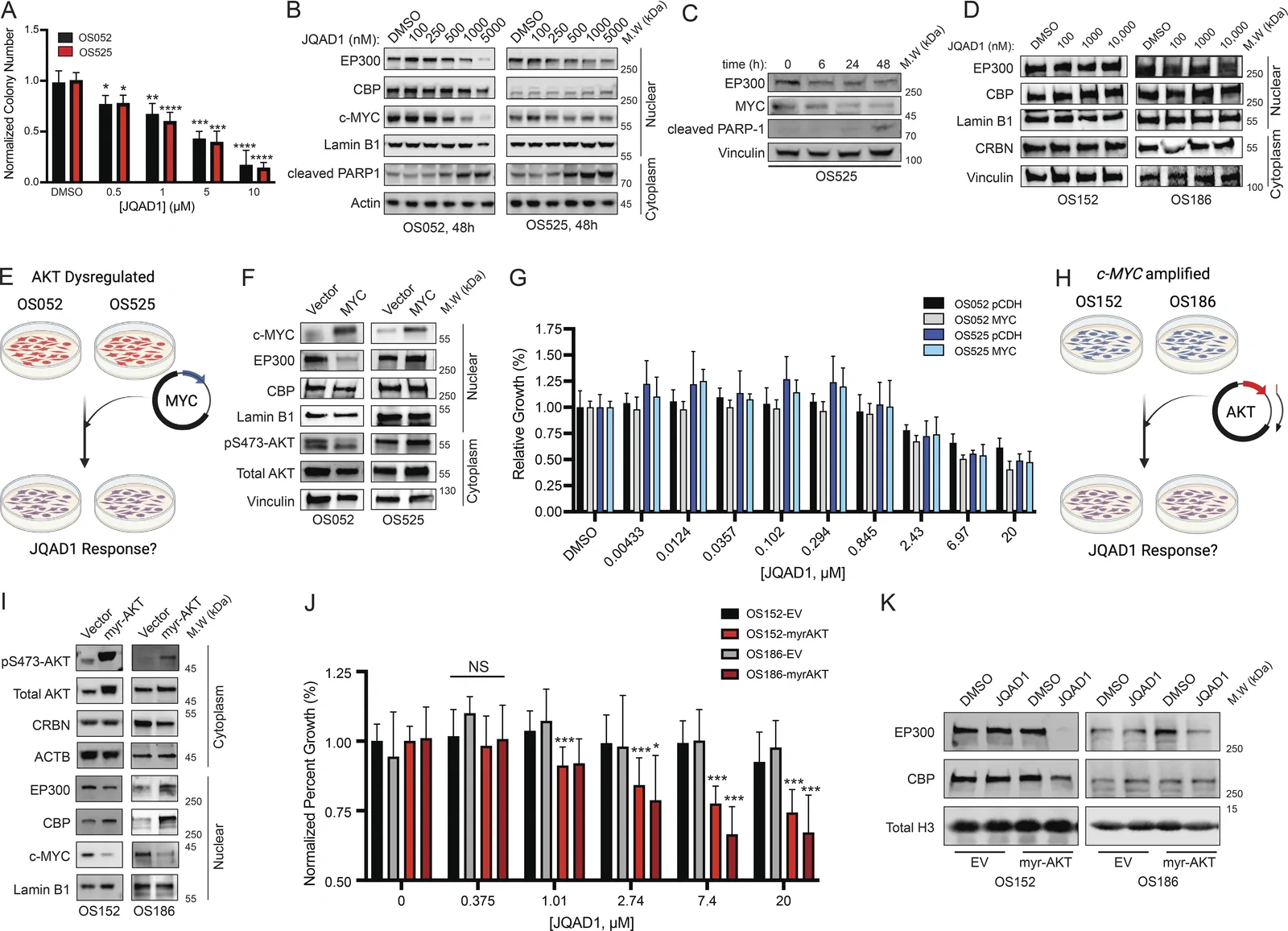
**Activated AKT drives OS cell response to JQAD1. (A)** Colony formation assay of OS052 and OS525 cells treated with a dose response of JQAD1 for 14 days. *n* = 4 independent biological replicates with four technical replicates/cell line and dose. Bars = SD; *P < 0.05, **P < 0.01, ***P < 0.001, ****P < 0.00001 by two-way ANOVA with post hoc Tukey tests, relative to DMSO. **(B)** Western blots of JQAD1-sensitive OS052 and OS525 cells treated with a dose response of JQAD1 for 48 h, followed by nuclear and cytoplasmic lysis. Data are representative of three independent lysates and blots. **(C)** Western blots of lysates from OS525 cells treated with 5 μM JQAD1 for 0, 6, 24, and 48 h. Data are representative of three independent lysates and blots. M.W, molecular weight. **(D)** Western blots of JQAD1-insensitive OS152 and OS186 cells treated with a dose response of JQAD1 for 48 h, followed by nuclear and cytoplasmic lysis. Data are representative of three independent lysates and blots. M.W, molecular weight. **(E)** Schema for c-MYC overexpression in JQAD1-sensitive OS052 and OS525 cell lines. Created in BioRender. Durbin, A. (2026) https://BioRender.com/s4mdyz6. **(F)** Western blots of OS052 and OS525 cells stably infected with lentiviruses encoding vector control or c-MYC. Data are representative of three independent lysates and blots. M.W, molecular weight. **(G)** Cells from F treated with a dose-response of JQAD1, with growth resolved by CellTiter-Glo analysis. Bars = SD, *n* = 3 independent experiments per sample, per dose. **(H)** Schema for myristoylated-AKT1 overexpression in JQAD1-insensitive OS152 or OS186 cells. Created in BioRender. Durbin, A. (2026) https://BioRender.com/s4mdyz6. **(I)** Western blots of OS152 and OS186 cells stably infected with lentiviruses encoding vector control or myr-AKT1 (myr-AKT). Data are representative of three independent lysates and blots. M.W, molecular weight. **(J)** Cells from I were treated with a dose-response of JQAD1, with data resolved by CellTiter-Glo analysis. Bars = SD, *n* = 3 independent experiments per sample, per dose. *P < 0.05, ***P < 0.001 by two-way ANOVA with post hoc Tukey test, relative to EV cells treated with the same dose. **(K)** Western blot of OS152 and OS186-EV and myr-AKT cells treated with DMSO or JQAD1 (5 μM) for 48 h. Data are representative of three independent lysates and blots. M.W, molecular weight. Source data are available for this figure: SourceData F2.

**Figure S2.**
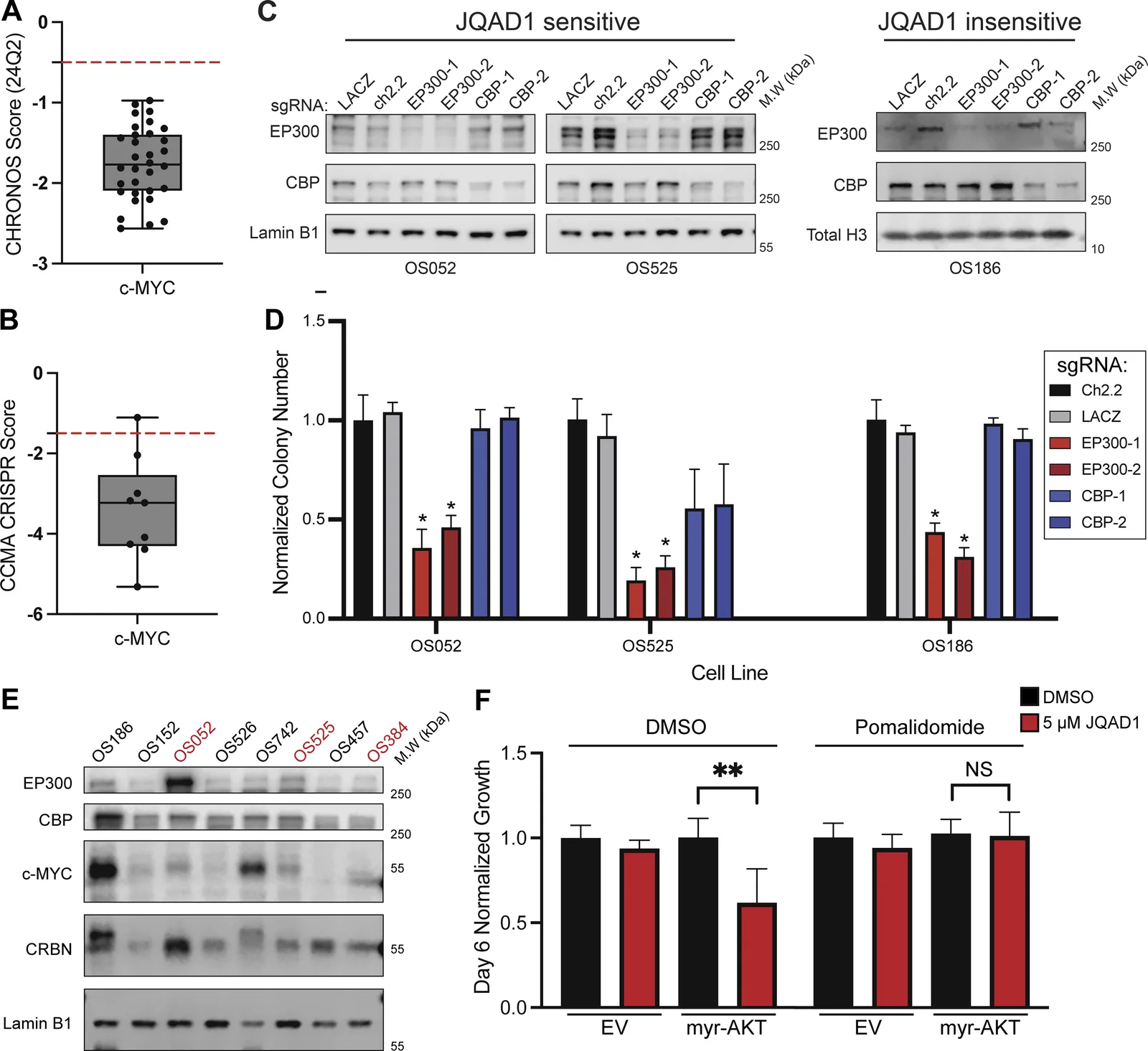
**EP300 genetic knockout reduces OS cell growth. (A and B)** CHRONOS or B CCMA CRISPR Score of c-MYC in OS (*n* = 27 [A], *n* = 9 [B]). Red line denotes dependency cutoff for relevant screen. **(C)** Western blotting of lysates prepared from OS052, OS525, or OS186 cells expressing Cas9 and infected with sgRNAs targeting EP300 (EP300-1, 2), CBP (CBP-1, 2), LACZ, or a gene desert on chromosome 2 (ch2.2). Data are representative of three independent infections, lysates, and blots. M.W, molecular weight. **(D)** Quantitation of colony formation assays of cells treated as in C. *n* = 4 independent biological experiments with four technical replicates per cell line and treatment. *P < 0.05 by two-way ANOVA with post hoc Tukey test relative to controls and CBP knockouts. Bars = SD. **(E)** Western blot of OS PDX cell lines for noted proteins for comparison. Data are representative of three independent lysates and blots. AKT-dysregulated PDX cell lines are highlighted in red. M.W, molecular weight. **(F)** Normalized Cell-Titer Glo assay results of OS152-EV and OS152-myr-AKT cells treated with 5 μM JQAD1 or DMSO for 6 days, alone or in the presence of 100 nM pomalidomide. Bars = SD, NS = not significant, **P < 0.01. Source data are available for this figure: SourceData FS2.

Our data suggested a correlation between AKT dysregulation and activity of JQAD1. To examine this correlation, we focused on two JQAD1-sensitive, AKT-dysregulated OS cell lines, OS052 (*PTEN*-deleted) and OS525 (*AKT1*-amplified). Consistent with growth inhibition driven by JQAD1 (Fig. 1 F), we observed that JQAD1 caused profound dose-dependent suppression of colony formation (Fig. 2 A). This was not associated with changes in cell cycle measurements (Fig. S1 I) nor with induction of osteoblastic differentiation when adjusting for loss of cell number (Fig. S1 J). Next, we treated these cells with a range of doses of JQAD1 for 48 h, and performed western blotting to assess changes in protein expression. JQAD1 caused dose-responsive degradation of EP300, without effects on CBP, in both cell lines (Fig. 2 B). Consistent with a growth slowing/induction of apoptosis phenotype, we observed that JQAD1 caused increased expression of the marker of apoptosis, cleaved PARP1 (Fig. 2 B). Despite these cell lines not having genomic *c-MYC* amplifications, all tested OS cell lines are dependent on c-*MYC* (Fig. S2, A and B). So, we also performed western blotting of c-MYC proteins, which demonstrated that loss of EP300 correlated with loss of c-MYC. To understand if c-MYC proteins were reduced by loss of EP300 or due to apoptotic induction, we treated OS525 cells with JQAD1 in a time course. JQAD1 caused loss of EP300 and c-MYC proteins ∼24 h prior to activation of apoptosis, measured by cleaved PARP1 (Fig. 2 C), indicating that loss of c-MYC is temporally separated from apoptosis. As a control, we treated two c-*MYC–*amplified, JQAD1-resistant cell lines, OS152 and OS186, with JQAD1, which failed to demonstrate effects on EP300 or CBP (Fig. 2 D). To confirm that these cells remained sensitive to genetic loss of *EP300* as predicted by DepMap analyses, we established Cas9-expressing OS052, OS525, and OS186 cells and infected these with sgRNAs targeting *EP300* (EP300-1, EP300-2), *CBP* (CBP-1, CBP-2), no expressed gene (*LACZ*), or a gene desert (*ch2.2*). Western blotting confirmed loss of the relevant target protein (Fig. S2 C). In colony formation growth assays, as predicted, we observed that loss of *EP300*, but not *CBP*, resulted in loss of cell growth in all three cell lines (Fig. S2 D). Thus, despite both types of cells being sensitive to genetic loss of *EP300*, JQAD1 causes loss of EP300, c-MYC, and activation of apoptosis in AKT-dysregulated, but not c-*MYC–*amplified, OS PDX cells.

Next, we sought to determine if the growth-inhibiting activity of JQAD1 was caused by the loss of c-MYC proteins in AKT-dysregulated cells. We established OS052 and OS525 AKT-dysregulated cells stably overexpressing either vector control or c-*MYC* and validated expression by western blotting (Fig. 2, E and F). While JQAD1 suppressed the growth of AKT-dysregulated cells, its effects were no different in vector control or c-MYC–overexpressing cells (Fig. 2 G). Thus, the pro-apoptotic effects of JQAD1 could not be rescued by overexpression of c-MYC in AKT-dysregulated cells.

A primary controller of response to JQAD1 is expression of the E3 receptor protein CRBN, which binds the heterobifunctional JQAD1 molecule to form a ternary complex (Barreto-Galvez et al., 2025; Durbin et al., 2022; Lee et al., 2023). We hypothesized that AKT-dysregulated cells may display higher CRBN expression. However, western blotting of OS PDX cell lines displayed no correlation between AKT-dysregulated status and CRBN expression, suggesting an alternate mechanism promoting JQAD1 activity in these cells (Fig. S2 E, red text denotes AKT-dysregulated lines). If c-MYC expression is independent of JQAD1 activity, and CRBN expression does not correlate with JQAD1 sensitivity, we reasoned that AKT activity may promote sensitivity to JQAD1. To examine this hypothesis, we used c-*MYC–*amplified, JQAD1-resistant OS152 and OS186 cells. We established stable cells expressing myristoylated, constitutively active AKT1 (myr-AKT) or vector control (Fig. 2 H). Western blotting confirmed that these cells express higher total and phosphorylated (pSer-473) AKT, without changes in EP300, CBP, or CRBN levels (Fig. 2 I). Intriguingly, myr-AKT reduced c-MYC expression (Fig. 2 I). Treatment of myr-AKT cells with JQAD1 caused a reduction in growth at high doses (>2 μM), which was not seen in JQAD1-insensitive vector control cells (Fig. 2 J). To test if these effects were driven by targeted protein degradation, we co-incubated OS152-empty vector (EV) and OS152-myr-AKT cells with combinations of JQAD1 and/or pomalidomide. Excess pomalidomide rescued the JQAD1-induced suppression of growth in OS152-myr-AKT cells (Fig. S2 F). To confirm effects on EP300, we next treated myr-AKT and EV cells with JQAD1 or vehicle control for 48 h. Western blotting demonstrated JQAD1-induced loss of EP300 proteins with minimal effect on CBP in myr-AKT, but not EV, cells (Fig. 2 K). Together, these data indicate that AKT enforces sensitivity to JQAD1-driven degradation of EP300 in OS cells in a c-MYC and CRBN-independent manner.

### AKT reprograms the transcriptome of OS cells by driving relocalization of EP300 and CBP

**Figure 3.**
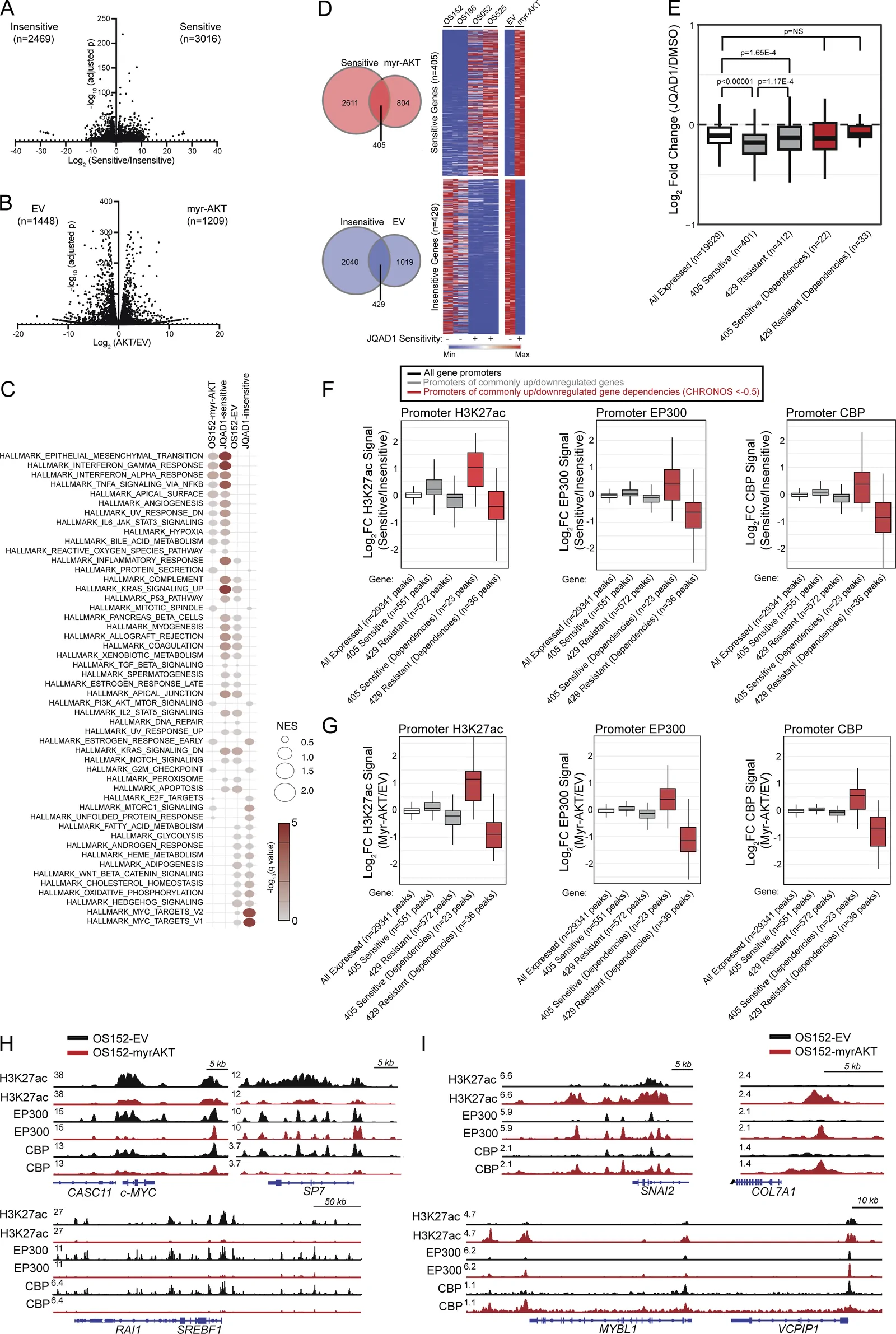
**Dysregulated AKT promotes transcriptional changes via relocalization of EP300, CBP, and H3K27ac to dependency genes. (A and B)** Volcano plot of differentially expressed genes identified by DESeq2 analysis of RNA-seq from (A) OS052, OS525 (sensitive) versus OS152 and OS186 (insensitive”) cell lines or (B) OS152-empty vector (EV) versus OS152-myr-AKT (myr-AKT) cell lines. Cutoffs: |Log_2_FC|<1 and adjusted P value < 0.05. *n* = 2 independent RNA extractions and sequencing reactions/sample (OS152-EV, OS152-myr-AKT), OS152), *n* = 3 (OS052, OS525, and OS186). **(C)** Bubble plot of significantly enriched Hallmark gene sets performed by GSEA of DESeq2 results, as in A and B. Color scale indicates −log_10_ (q-value). **(D)** Venn diagram and heatmap analyses of overlapping genes from sensitive/myr-AKT upregulated genes, and insensitive/EV upregulated genes. **(E)** Box-and-whisker plots showing Log_2_FCs in spike-in-normalized RNA-seq expression from OS525 cells treated for 24 h with DMSO or 1 μM JQAD1. Gene subsets are all expressed transcripts (white), 405 genes with elevated expression in JQAD1-sensitive cells (sensitive, gray), 429 genes with elevated expression in JQAD1-insensitive cells (insensitive, gray), and the genetic dependencies found within these subsets of genes (red). *n* = 3 independent biological replicates sequenced. Box-and-whisker plots illustrate the median (wide line), quartiles (box), and 1.5 * interquartile range (whiskers). **(F and G)** Box-and-whisker plots showing Log_2_FCs in H3K27ac, EP300, and CBP coverage quantified from ChIP-seq assays at promoters of specified gene sets, in JQAD1-sensitive/insensitive cells (F) or OS152-AKT/OS152-EV (G) samples. Gene sets are as specified in E. All boxplot comparisons are statistically significant by two-sided Welch’s *t* tests without multiple-testing correction, relative to all promoters and other samples, save for those noted in Table S8. Box-and-whisker plots illustrate the median (wide line), quartiles (box), and 1.5 * interquartile range (whiskers). **(H)** Representative IGV gene tracks of ChIP-seq signal of H3K27ac, EP300, and CBP at gene dependency loci downregulated in AKT-dysregulated cells, in OS152-EV (black) and OS152-myr-AKT (red) cells. Loci shown are c-*MYC*, *SP7*, and *SREBF1*. **(I)** Representative IGV gene tracks of ChIP-seq signal of H3K27ac, EP300, and CBP at gene-dependency loci upregulated in AKT-dysregulated cells in OS152-EV (black) and OS152-myr-AKT (red) cells. Loci shown are *SNAI2*, *COL7A1*, and *MYBL1-VCPIP1*.

EP300 and CBP control gene enhancer and promoter elements partly by catalyzing acetylation of H3K27ac. This recruits additional transcriptional complex members to drive transcriptional output of associated genes. Thus, we next sought to determine if AKT promotes transcriptional reprogramming, resulting in sensitivity to targeted degradation of EP300. We performed RNA-seq analysis in two JQAD1-sensitive, AKT dysregulated (OS052, OS525), and two JQAD1-insensitive, c-*MYC–*amplified OS cell lines (OS152, OS186). Comparison demonstrated relative overexpression of 2469 genes in c-*MYC–*amplified JQAD1-insensitive cells and 3,016 genes in AKT-dysregulated, JQAD1-sensitive cells (Fig. 3 A). To identify which changes were a direct consequence of AKT activity, we performed RNA-seq in OS152-EV and OS152-myr-AKT cells. Similar comparisons identified 1,448 genes with relative overexpression in EV cells, and 1,209 genes in myr-AKT cells (Fig. 3 B). Comparing the differentially expressed genes between sensitive and insensitive cells, and myr-AKT and EV cells, and then examining these results using Gene Set Enrichment Analysis (GSEA), we identified enrichment of several Molecular Signatures Database (MSigDB) Hallmark gene sets. In both myr-AKT and JQAD1-sensitive cells, we observed relative enrichment of genes curated in Epithelial_to_Mesenchymal_Transition, IFNA and IFNG_Responses, and TNFA_Signaling_by_NFKB, among others (Fig. 3 C). These data align with prior studies demonstrating that EP300 promotes OS cell mesenchymal properties, such as metastasis (Morrow et al., 2018). Moreover, we also observed relatively elevated expression of several gene sets in *c-MYC–*amplified, JQAD1-insensitive cells and OS152-EV cells, including both MYC_Targets_V1 and V2 (Fig. 3 C). These data indicated transcriptional pathway signature similarities between JQAD1-sensitive and OS152-myr-AKT cells, as well as between JQAD1-insensitive and OS152-EV cells.

**Figure S3.**
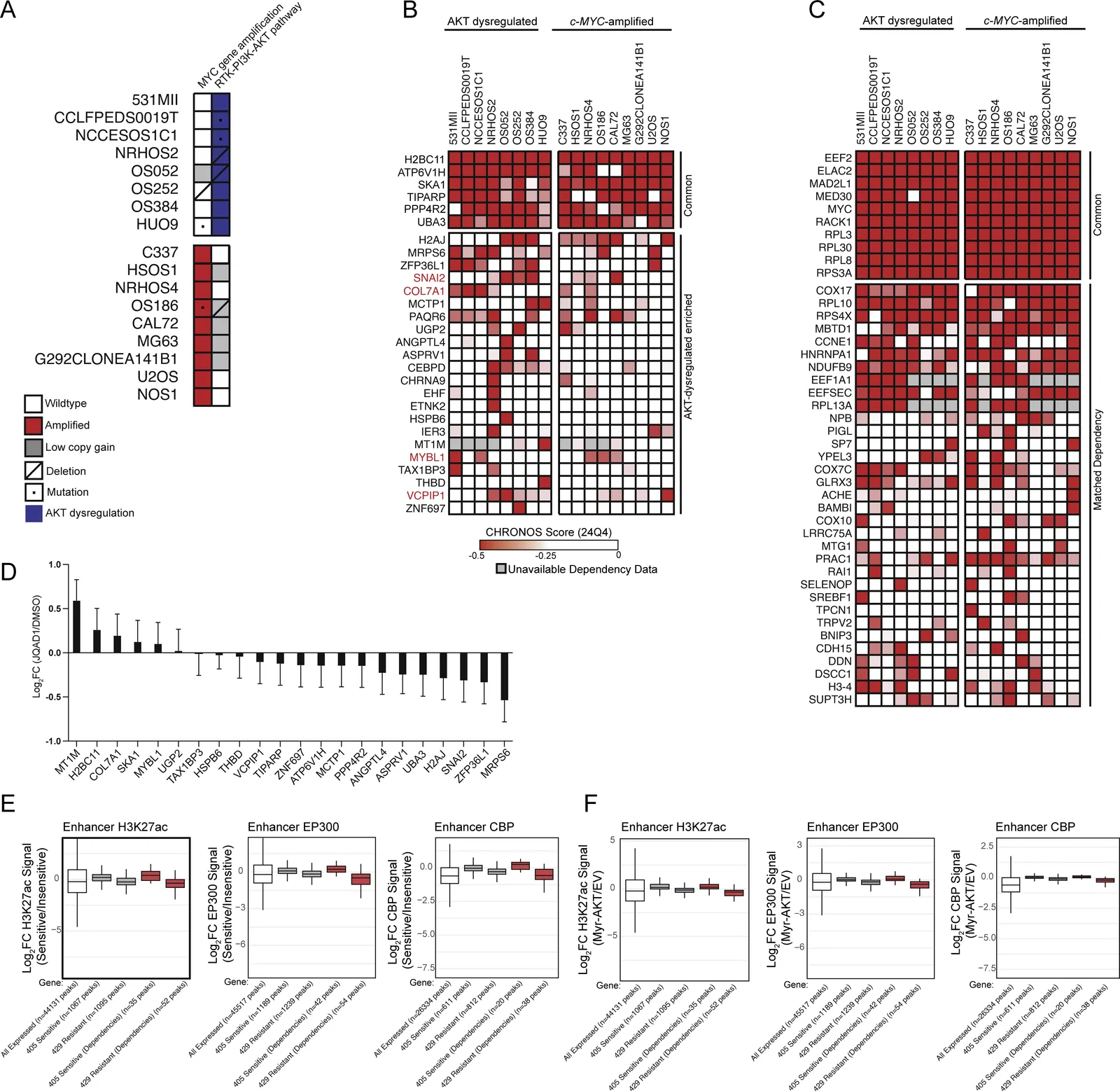
**Myristoylated-AKT1 promotes the expression of dependencies found in AKT-dysregulated cells. (A)** OS cell lines from DepMap (24Q2 release) were identified as being predominantly c-*MYC–*amplified or AKT-dysregulated, as described in the Materials and methods. **(B)** CHRONOS dependency score (24Q2 release) plotted for *n* = 405 genes commonly upregulated in JQAD1-sensitive, AKT-dysregulated OS052, OS525, and OS152-myr-AKT cells, compared with c-*MYC–*amplified OS152, OS186 cells, and OS152-EV cells. Demonstrated are those genes with dependency (CHRONOS Score < −0.5) in at least one cell line, separated into common essential genes and those enriched in AKT-dysregulated cells versus *c-MYC*–amplified cells. **(C)** Analysis as in B, though for genes enriched in c-*MYC–*amplified, JQAD1-insensitive cells, compared with AKT-dysregulated, JQAD1-sensitive cells (*n* = 429). Genes are separated into common essentials and those with matched dependency between c-*MYC–*amplified and AKT-dysregulated cell lines. **(D)** Dependencies (*n* = 22) within the 405 genes upregulated in JQAD1-sensitive cells that are found downregulated in OS525 cells after 24 h of treatment with JQAD1 by RNA-seq. Demonstrated is the Log_2_FC of JQAD1-treated samples compared with DMSO. Bars = SD, *n* = 3 independent biological replicates. **(E and F)** Box-and-whisker plots showing Log_2_FCs in H3K27ac, EP300, and CBP coverage quantified from ChIP-seq results at enhancers associated with specified gene sets, in JQAD1-sensitive/insensitive cells (E) or OS152-AKT/OS152-EV (F) samples. Gene subsets are all expressed transcripts (white), 405 genes with elevated expression in JQAD1-sensitive cells (sensitive, gray), 429 genes with elevated expression in JQAD1-insensitive cells (insensitive, gray), and the genetic dependencies found within these subsets of genes (red). All boxplot comparisons are statistically significant by two-sided Welch’s *t* tests without multiple-testing correction, relative to all associated enhancers and other samples, save for those noted in Table S8. Box-and-whisker plots illustrate the median (wide line), quartiles (box), and 1.5 * interquartile range (whiskers).

Next, we sought to identify genes responsive to differential AKT activity. To do so, we integrated differentially expressed genes elevated in sensitive and OS152-myr-AKT cells, or insensitive and OS152-EV cells. This identified 405 genes more highly expressed in both endogenous JQAD1-sensitive cells (OS052, OS525) and OS152-myr-AKT cells, and 429 genes more highly expressed in JQAD1-insensitive cells and OS152-EV controls (Fig. 3 D and Table S4). To define which of these 405 or 429 gene sets may be OS dependencies subject to regulation by AKT, we identified cell lines in DepMap with mutations indicating either AKT dysregulation (*n* = 8) or c-*MYC* amplification (*n* = 9), and excluded those with evidence of both AKT dysregulation and alterations in c-*MYC*. We then compared these two groups. (Fig. S3 A; and Tables S4, S5, S6, and S7). Integration of dependency scores with these 405 “JQAD1-sensitive/myr-AKT” and 429 “JQAD1-insensitive/EV” genes revealed, as expected, that JQAD1 sensitive/myr-AKT upregulated genes were enriched for genetic dependency in AKT-dysregulated cells (Fig. S3 B). As expected, due to the comparison involving myr-AKT overexpression in c-*MYC–*amplified cells, the JQAD1 insensitive/EV upregulated gene group was not enriched for dependency in c-*MYC*–amplified cells (Fig. S3 C). Next, we tested the effect of JQAD1 on expression of these gene subsets in OS525 AKT-dysregulated cells. JQAD1 treatment for 24 h led to a general trend toward downregulation of gene expression (Fig. 3 E, median log_2_ fold-change [Log_2_FC] = −0.11). This effect was significantly more pronounced at the 405 genes enriched in JQAD1-sensitive/myr-AKT cells, as compared with the 429 genes enriched in JQAD1-insensitive/EV cells (Fig. 3 E, P = 1.17e-4). These findings were similar, though did not achieve statistical significance, when examining the genetic dependencies in this subset, though the dependency groups were marked by few genes (Fig. 3 E and Fig. S3 D). These findings indicate that myr-AKT expression is sufficient to transcriptionally reprogram OS cells and that JQAD1 has global effects on transcription with enhanced effects on this program.

Since EP300 and CBP catalyze the active enhancer and promoter H3K27ac mark, we hypothesized that AKT expression may promote transcriptional reprogramming through relocalization of EP300 and/or CBP. We performed chromatin immunoprecipitation coupled to massively parallel, high-throughput sequencing (ChIP-seq) using antibodies against EP300 or CBP or their catalyzed mark H3K27ac, in each of these cell lines. Comparison of H3K27ac signal and EP300 or CBP binding genome-wide at all gene promoters and enhancers demonstrated that most are consistently marked between JQAD1-sensitive and -insensitive cells (Fig. 3 F and Fig. S3 E, white boxplots) and OS152-AKT and OS152-EV cells (Fig. 3 G and Fig. S3 F). Thus, we next examined the presence of these marks and coactivator binding at the promoters and enhancers of the 405 and 429 genes upregulated in JQAD1-sensitive or -insensitive cells (Fig. 3, F and G; and Fig. S3, E and F; gray boxplots). We observed differential marking of H3K27ac and EP300 and CBP binding at these regions in genes differentially expressed in either JQAD1-sensitive or -insensitive cells (statistics for comparisons with promoters and enhancers are found in Table S8). These differences were exacerbated when examining the promoters or enhancers of dependency genes found within these signatures in JQAD1-sensitive or -insensitive cells (Fig. 3, F and G; and Fig. S3, E and F; red boxplots). These data indicate that in both endogenously AKT-dysregulated cells and c-*MYC*–amplified cells, EP300, CBP, and their catalyzed mark H3K27ac are found at the gene promoters and enhancers of expressed genes, and more prominently at the gene promoters and enhancers of genetic dependencies. Further, ectopic expression of AKT is sufficient to alter gene expression through relocalization of EP300, CBP, and H3K27ac to new gene promoters and enhancers, including context-specific genetic dependencies. Examples of critical gene dependency loci at which expression of AKT enforces either reduced or gained H3K27ac, EP300, and CBP binding are shown in Fig. 3, H and I. Expression of myr-AKT in OS152 cells causes loss of H3K27ac, EP300, and CBP binding at the loci of highly expressed transcription factor gene dependencies in c-*MYC–*amplified cells *SP7*, *SREBF1*, and c-*MYC* (Fig. 3 H). These data also provide an explanation for the loss of c-MYC expression with expression of myr-AKT (Fig. 2 I). In contrast, we observed induced H3K27ac, EP300, and CBP binding at the *MYBL1*, *VCPIP1*, *SNAI2*, and *COL7A1* loci, which are highly expressed and dependent in AKT-dysregulated cells (Fig. 3 I). Consistent with the observed enrichment for epithelial-to-mesenchymal (EMT) signatures in our RNA-seq datasets (Fig. 3 C), we also observed gain of enhancer and promoter marking and EP300 and CBP binding at the EMT-associated *SNAI2* and *COL7A1* loci (Fig. 3 I). Together, these data indicate that AKT reprograms OS cells by enforcing relocalization of EP300 and CBP, which alters promoter and enhancer acetylation (H3K27ac) genome-wide, to drive changes in transcription, including that of context-specific genetic dependencies.

### AKT inhibition and EP300 degradation synergistically suppress OS cell growth

The observation that multiple members of the PI3K–AKT–mTOR signaling pathway are dysregulated in OS has led to studies nominating this pathway for potential therapeutic intervention (Perry et al., 2014; Vasan and Cantley, 2022). However, despite AKT-dysregulated OS cells demonstrating mutational activation of the AKT pathway, treatment with AKT inhibitors, including the small-molecule MK-2206, had modest activity on tumor growth in vivo (Sayles et al., 2019). Since JQAD1 was selectively active in AKT-dysregulated OS cells, we next explored whether combinations of the allosteric AKT inhibitor MK-2206 with EP300-targeted degraders (JQAD1) would combine positively.

**Figure S4.**
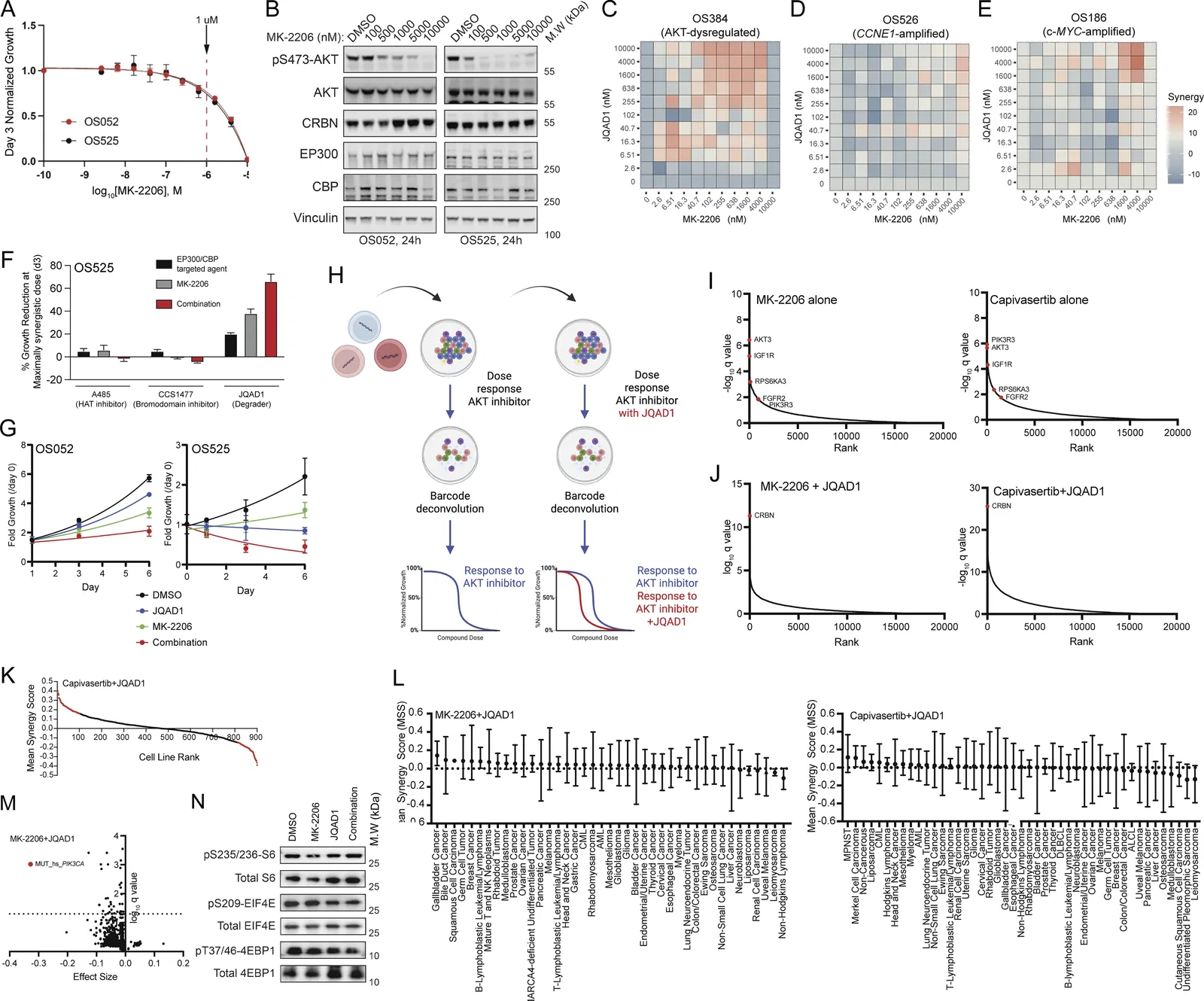
**JQAD1 synergizes with AKT inhibitors in vitro in OS and other cancer cells. (A)** Dose-response testing of MK-2206 in AKT-dysregulated cells (OS052, OS525) after 3 days, by CellTiter-Glo. Bars = SD, *n* = 3 independent biological replicates. **(B)** Western blotting of noted proteins after 24 h treatment with dose-response of MK-2206 in OS052 and OS525 cells. Data are representative of three independent lysates and blots. M.W, molecular weight. **(C–E)** OS384 (C), OS526 (D), and OS186 (E) cells were treated with MK-2206 or JQAD1 alone or in combination at the noted doses for 3-day periods, and cell growth was resolved by CellTiter-Glo analysis. Data shown are the average of *n* = 3 independent replicates per test, with synergy modeled by ZIP score. **(F)** OS525 AKT-dysregulated cells were treated as in C–E, with either A485+MK-2206, CCS1477+MK-2206, or JQAD1+MK-2206. The point of maximal synergy was determined by ZIP score, and the growth response at these doses is shown either individually or in combination. Data shown are the average of *n* = 3 independent replicates per test. Bars = SD. **(G)** CellTiter-Glo growth curves for OS052 or OS525 cells treated with DMSO, JQAD1, MK-2206, or MK-2206+JQAD1, all at 1.6 mM. Bars = SD, *n* = 3 independent biological replicates per dose, per time point. **(H)** Schema of PRISM combination screening, using dose-response of AKT inhibitors (MK-2206 or capivasertib) alone and in combination with 1 μM anchor dose of JQAD1. Created in BioRender. Durbin, A. (2026) https://BioRender.com/r7tu4zz. **(I)** Gene expression analysis across all cell lines treated with a dose-response of MK-2206 (*n* = 871), or capivasertib (*n* = 912), demonstrating enrichment of higher-expressed PI3K–AKT pathway members. **(J)** Gene expression analysis across all cell lines treated with a dose-response of MK-2206 with anchor dosing of JQAD1 (*n* = 871), or with a dose-response of capivasertib with anchor dosing of JQAD1 (*n* = 912), demonstrating enrichment of higher-expressed *CRBN*. **(K)** MSS, calculated as detailed in the Materials and methods across 912 cell lines tested with capivasertib + JQAD1. Top 100 and bottom 100 most and least synergistic cell lines noted in red. Additive/synergistic cells with MSS>0 (466/912 = 51.09%), and antagonistic cells with MSS<0 (446/912 = 48.9%). **(L)** MSS for all tumor types, ranked by mean MSS, in cells treated with MK-2206+JQAD1 (left) or capivasertib + JQAD1 (right). Bars represent data spread. **(M)** Mutational analysis of all cell lines examining for correlation between the effect of JQAD1+MK-2206 compared with MK-2206 alone demonstrates enrichment for hotspot mutations in *PIK3CA*. Mutational data retrieved from the CCLE, DepMap 24Q2 release. **(N)** Western blot of OS052 cells treated with either DMSO, MK-2206 (1 µM), JQAD1 (5 µM), or the combination for 48 h followed by nuclear and cytoplasmic lysis. Data are representative of three independent lysates and blots. M.W, molecular weight. ZIP, zero interaction potency. Source data are available for this figure: SourceData FS4.

**Figure 4.**
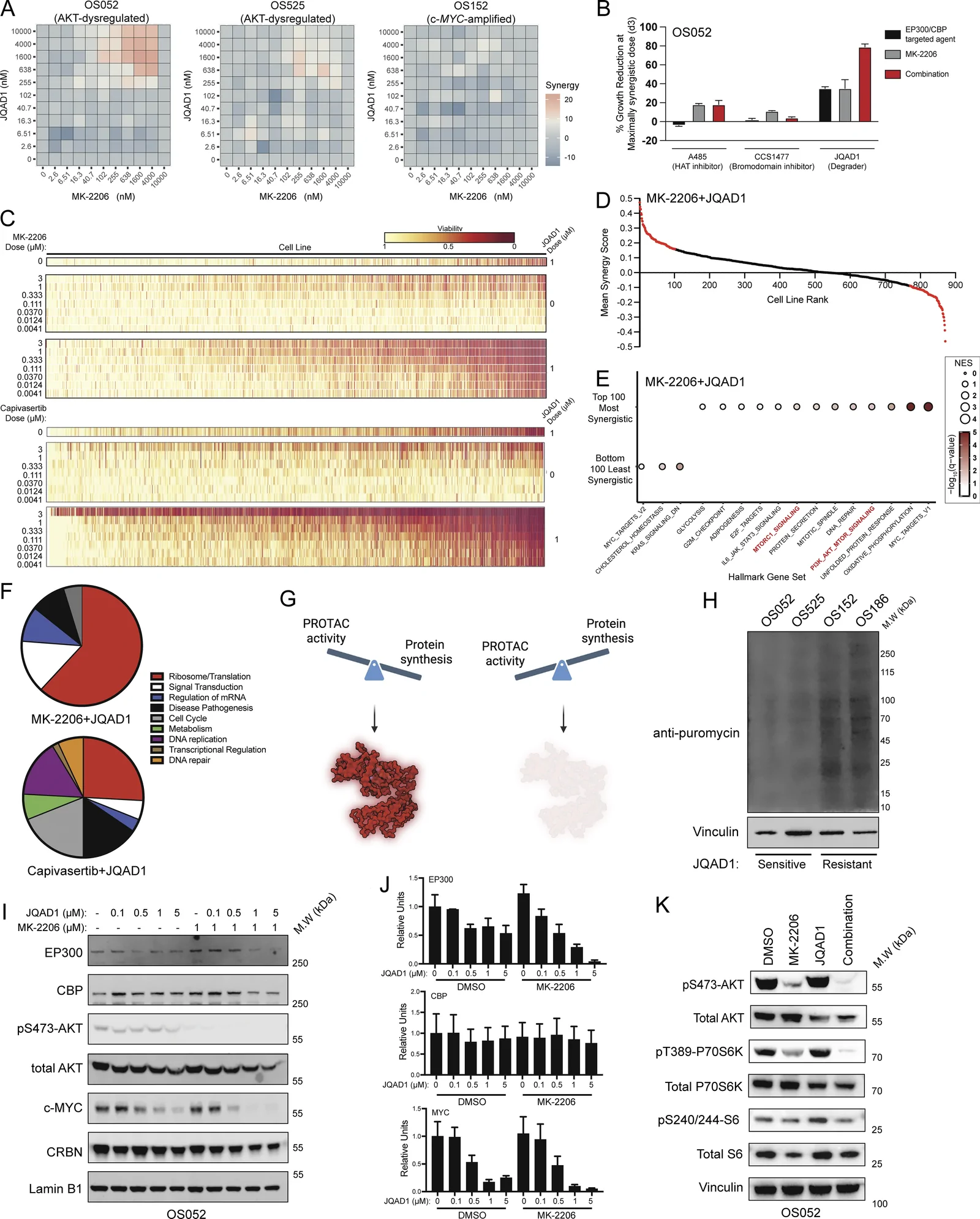
**AKT inhibition synergizes with EP300 degradation in AKT-dysregulated OS cells. (A)** OS052, OS525, and OS152 cells were treated with MK-2206 and JQAD1 in combination at the noted doses for 3 days, and cell growth was assessed by CellTiter-Glo analysis. Data shown are the average of *n* = 3 independent replicates per test, with synergy modeled by the zero interaction potency (ZIP) algorithm. **(B)** OS052 AKT-dysregulated cells were treated as in A, with either A485+MK-2206, CCS1477+MK-2206, or JQAD1+MK-2206. The point of maximal synergy was determined by ZIP score, and the growth response at these doses is shown either individually or in combination. Data shown are the average of *n* = 3 independent replicates per test. Bars = SD. **(C)** Heatmap of response to JQAD1 alone (1 μM), dose response of MK-2206, or the combination of these, across 871 cancer cell lines (top) or JQAD1 alone (1 μM), dose response of capivasertib, or the combination of these, across 912 cancer cell lines (bottom) by PRISM analysis. *n* = 3 replicates per dose, per cell line. **(D)** MSS, calculated as detailed in the Materials and methods across 871 cell lines tested with MK-2206+JQAD1. Top 100 and bottom 100 most and least synergistic cell lines noted in red. Additive/synergistic cells with MSS>=0 (537/871 = 61.7%), and antagonistic cells with MSS<0 (334/871 = 38.3%). **(E)** Bubble plot of GSEA results comparing the 100 most and least synergistic cell lines, using analysis of the Hallmark gene sets from MSigDB in the MK-2206+JQAD1 dataset. Cutoff of FDR q <0.25. **(F)** Enriched MSIGDB C2 gene sets (WikiPathways, REACTOME, KEGG ontologies) with FDR q <10^−9^ in synergistic cells are shown, comparing GSEA of the 100 most and least synergistic cell lines for each dataset. **(G)** Model of interactions between protein synthesis rate and PROTAC function. Created in BioRender. Durbin, A. (2026) https://BioRender.com/1mtm3gm. **(H)** Nascent protein synthesis western blot of OS052, OS525 (JQAD1-sensitive) and OS152, OS186 (JQAD1-insensitive) cells labelled with puromycin for 20 min prior to whole-cell lysis. Data are representative of three independent lysates and blots. M.W, molecular weight. **(I)** Western blot of OS052 cells treated with JQAD1 in dose-response, ± MK-2206 at 0 or 1 μM, for 48 h. Data are representative of three independent lysates and blots. M.W, molecular weight. **(J)** Quantitation of western blots from I for EP300, CBP, and c-MYC protein levels. Bars = SD. **(K)** Western blot of OS052 cells treated with either DMSO, MK-2206 (1 µM), JQAD1 (5 µM), or the combination for 48 h followed by nuclear and cytoplasmic lysis. Data are representative of three independent lysates and blots. M.W, molecular weight. Source data are available for this figure: SourceData F4.

First, we established the day-three IC_50_ of MK-2206 in vitro (Fig. S4 A). Then, we tested if these doses would potentiate JQAD1 function. We treated OS052 and OS525 cells with a range of concentrations of JQAD1 (100 nM−10 μM), and performed western blotting to assess whether MK-2206 would affect expression of CRBN, EP300, or CBP. Despite potent effects on phospho-Ser-473-AKT, MK-2206 did not affect the expression of CRBN, EP300, or CBP (Fig. S4 B), suggesting that MK-2206 would not likely disrupt the degrader activity of JQAD1. Next, we tested JQAD1, alone and in combination with MK-2206, in a combination dose-response format, and resolved effects on cell growth in six OS PDX cell lines by CellTiter-Glo analysis. Combination treatment demonstrated synergistic responses in all three AKT-dysregulated cell lines (Fig. 4 A and Fig. S4 C). This combination was neutral in OS152 c-*MYC–*amplified and OS526 *CCNE1-*amplified cells. Intriguingly, however, this combination was synergistic in OS186 c-*MYC–*amplified cells, which also contain loss of *PTEN* (Fig. 1 G). Thus, while the combination of AKT inhibition and EP300 degradation is additive/synergistic in AKT-dysregulated cells, it is less likely to be so in OS cells with only c-*MYC* amplification. (Fig. 4 A and Fig. S4, C–E). EP300 and CBP are multidomain-containing proteins that may be targeted by inhibition of their HAT or BRDs, using high-potency molecules that bind to the HAT (A485) or BRD (CCS1477) (Lasko et al., 2017; Michaelides et al., 2018; Welti et al., 2021). Since the mechanism of EP300 and CBP targeting may yield distinct transcriptomic and phenotypic effects (Shendy et al., 2024; Zucconi et al., 2019), we performed similar testing of MK-2206 in combination with either A485 or CCS1477 in OS052 and OS525 cells and compared these with our results with JQAD1. We determined the doses of A485+MK-2206, CCS1477+MK-2206, and JQAD1+MK-2206 at which we observed maximal synergy between compound pairs and compared the effect of individual or combination compound treatments in both cell lines at these doses. We observed minimal inhibition of growth with A485+MK-2206 or CCS1477+MK-2206 in either cell line at the maximally synergistic doses (Fig. 4 B and Fig. S4 F). In contrast, at the doses of JQAD1 or MK-2206 eliciting maximal synergy, each compound reduced growth, though synergistically more so when combined (Fig. 4 B and Fig. S4 F). Correspondingly, CellTiter-Glo growth assays using vehicle control or 1.6 μM of JQAD1 or MK-2206, alone or together in both AKT-dysregulated cell lines, demonstrated enhanced suppression of growth with combination treatments (Fig. S4 G). Together, these data indicate that combinations of MK-2206 and JQAD1 synergistically suppress AKT-dysregulated OS cell growth in vitro.

### JQAD1 broadly synergizes with AKT inhibitors across cancer types

AKT dysregulation, by mutation/gain of *PIK3CA*, *AKT*, or loss of *PTEN*, is a common event across many different human cancers (Millis et al., 2016; Zhang et al., 2017). So, we sought to understand if this observed synergistic interaction between JQAD1 and MK-2206 was restricted to OS cells or occurred more broadly. We performed a high-throughput, PRISM, barcoded cell line–based screen using 871 cancer cell lines (Fig. S4 H) from the CCLE (Corsello et al., 2020; Ghandi et al., 2019; Yu et al., 2016) to test the effects of escalating MK-2206 dosage, either alone or in combination with 1 μM anchor dosing of JQAD1. While 1 μM dosing of JQAD1 alone or MK-2206 in a dose-response series reduced the area-under-the-curve (AUC) measurement, indicating suppression of growth, for a fraction of cell lines (Fig. 4 C), the combination of these two demonstrated enhanced inhibition of growth (Fig. 4 C). To ensure this finding was not restricted to MK-2206, we performed a similar analysis of 912 cell lines, using a structurally-distinct AKT inhibitor, capivasertib (Dilawari et al., 2024), which demonstrated similar results (Fig. 4 C). Consistent with compound mechanisms of action, cells with enhanced responses to MK-2206 and capivasertib displayed elevated gene expression of AKT pathway members, including *IGF1R*, *FGFR2*, *PIK3R3*, *AKT3*, and *RPS6KA3* (Fig. S4 I), while cells with enhanced responses to the combination of either MK-2206 or capivasertib with JQAD1 had higher expression of the JQAD1 E3 ligase receptor, *CRBN* (Fig. S4 J). Thus, known compound mechanisms of action were reflected in these experiments.

Next, using the individual responses to dose-specific MK-2206 or 1 μM JQAD1, we calculated a mean synergy score (MSS) by an excess-above-Bliss metric (Chou, 2010). 537 (61.65%) or 466 (51.09%) of tumor cell lines had MSS ≥ 0, indicating additivity or synergy with JQAD1 and MK-2206, or capivasertib, combinations. In contrast, 334 (38.35%) or 446 (48.9%) of tumor cell lines displayed MSS<0, indicating antagonism (Fig. 4 D, Fig. S4 K, and Table S9). Organization of these results by tumor lineage, limited to those with >3 cell lines per lineage, demonstrated substantial data spread across lineages, suggesting tumor type was not a major driver of synergy (Fig. S4 L). Further, integrating MSS results with damaging or hotspot point mutation data from the CCLE identified that the largest effect size associated with synergy was hotspot mutations in *PIK3CA* (Fig. S4 M). However, other genes of interest were not enriched for mutation, including copy number alterations or mutations in genes such as *AKT1*, *AKT2*, *AKT3*, *PTEN*, *c-MYC*, *MYCN*, or *LMYC*. Since gene expression of known pathway members, such as *CRBN* for JQAD1 and *AKT3* for MK-2206 and capivasertib, was associated with compound mechanism of action (Fig. S4, I and J), we next sought to determine if we could use transcriptomic profiling to identify known pathways whose members were more highly expressed in synergistic or antagonistic cell lines. We identified the top 100 most synergistic and bottom 100 least synergistic (i.e., most antagonistic) cell lines and extracted their whole transcriptomic RNA-seq–based expression profiles from the CCLE (Fig. 4 D and Fig. S4 K, red-marked data points). GSEA showed that genes in the Hallmark PI3K–AKT–mTOR_Signaling and mTORC1_Signaling gene sets were upregulated in the most synergistic cell lines (Fig. 4 E). These suggested that cells sensitive to JQAD1 and MK-2206 may have enhanced reliance on AKT signaling through mTORC1.

To expand upon this analysis and nominate cellular processes that may be involved in molecular synergy, we again performed a GSEA comparing the top and bottom 100 cell lines, but examining the molecular signatures database (MSigDB) C2 Ontologies, focusing on Kyoto Encyclopedia of Genes and Genomes (KEGG), WikiPathways, and REACTOME pathways. Narrowing to the highest enriched ontologies (FDR < 10^−9^), we identified 21 showing significant upregulation in MK-2206+JQAD1 synergistic cells, of which 13 (61.9%) were involved in control of the ribosome/protein translation, a known downstream target of the AKT pathway through mTORC1 (Fabbri et al., 2021) (Fig. 4 F and Table S10). Similar observations, though to a lesser extent, were identified with capivasertib + JQAD1 synergistic cells (Fig. 4 F and Table S10). There were fewer ontologies with an FDR = 0 in antagonistic cells, and so by relaxing criteria to an FDR < 0.0001, we identified those ontologies showing elevated expression in antagonistic models, including DNA damage repair and cholesterol biosynthesis (Fig. 4 E and Table S10). Thus, cells demonstrating JQAD1+AKT inhibitor synergy displayed transcriptional signatures suggestive of elevated protein synthesis.

### Low protein synthesis rates correlate with degrader sensitivity

One model for PROTAC function implies competition between protein synthesis and degradation rates (Mares et al., 2020) (Fig. 4 G). By this model, the PROTAC effect is enhanced in cells with reduced protein synthesis rates due to a failure to resynthesize protein species that have been depleted by proteasomal degradation. Since cells displaying synergy between MK-2206 and JQAD1 have transcriptional signatures of elevated protein synthesis, we hypothesized that AKT inhibitor-mediated suppression of protein synthesis may be a mechanism of potentiation of JQAD1 activity. To test this, we examined nascent protein synthesis rates in JQAD1-sensitive and insensitive cell lines using puromycin labeling assays. Western blotting of lysates prepared from OS cells pulsed with puromycin showed increased labeling in JQAD1-insensitive OS152 and OS186 cells, compared with OS052 and OS525 JQAD1-sensitive cells, suggesting a slower rate of nascent protein synthesis in JQAD1-sensitive cells (Fig. 4 H).

Next, we tested the ability of AKT inhibition to potentiate the JQAD1 effect on EP300 by treating JQAD1-sensitive OS052 cells with a range of doses of JQAD1, along with either DMSO or MK-2206 at 1 μM. High-dose JQAD1 depleted EP300, resulting in loss of c-MYC proteins. However, when co-incubated with MK-2206, JQAD1 reduced both EP300 and c-MYC proteins at lower doses (Fig. 4, I and J). AKT control of protein synthesis is mediated via mTORC1 through distinct pathways, including phosphorylation-dependent activation of p70S6K or repression of 4EBP1 (He et al., 2025). To understand which pathway may be driving these effects, we treated OS052 cells with vehicle, JQAD1 (5 μM), MK-2206 (1 μM), or their combination for 48 h and analyzed the phosphorylation status of multiple pathway members. Western blotting confirmed loss of phospho-Ser473-AKT, but also demonstrated reductions in phospho-Thr389-p70S6K and the p70S6K target phospho-Ser-240/244 of ribosomal protein S6 with MK-2206 and combination treatments (Fig. 4 K). In contrast, other mechanisms controlling protein synthesis, such as the AKT-mTOR–directed phospho-Thr37/46-4EBP1 and phospho-Ser209-EIF4E, or the independent p90RSK-directed phosphorylation sites phospho-Ser235/236 of ribosomal protein S6, were unaffected (Fig. S4 N). These data indicate that one mechanism by which JQAD1 and MK-2206 function synergistically is through combined suppression of protein synthesis, facilitating enhanced EP300-specific degradation and subsequent transcriptional downregulation of essential genes.

### JQAD1 and MK-2206 synergize in AKT-dysregulated OS in vivo

Finally, we sought to determine if JQAD1 and MK-2206 displayed synergy in vivo. We orthotopically xenografted 2 × 10^6^ OS052 or 1.5 × 10^6^ OS525 cells into the right paratibial site of NMRI-*nu/nu* mice and followed for tumor engraftment by serial micro-CT analyses. The experimentally determined maximally tolerated dose of JQAD1 is 40 mg/kg twice daily by i.p. injection (Durbin et al., 2022), and MK-2206 is 180 mg/kg, three times weekly by oral gavage (Gorlick et al., 2012). To draw out potential synergistic interactions with MK-2206, we reduced the dosages of both compounds. Thus, when tumors started to grow by micro-CT and caliper measurement (tumors palpable and increasing on serial measurements), animals were randomized to receive either daily vehicle control, JQAD1 (20 mg/kg/day, i.p. injection), MK-2206 (120 mg/kg, three times weekly by oral gavage), or combination.

**Figure 5.**
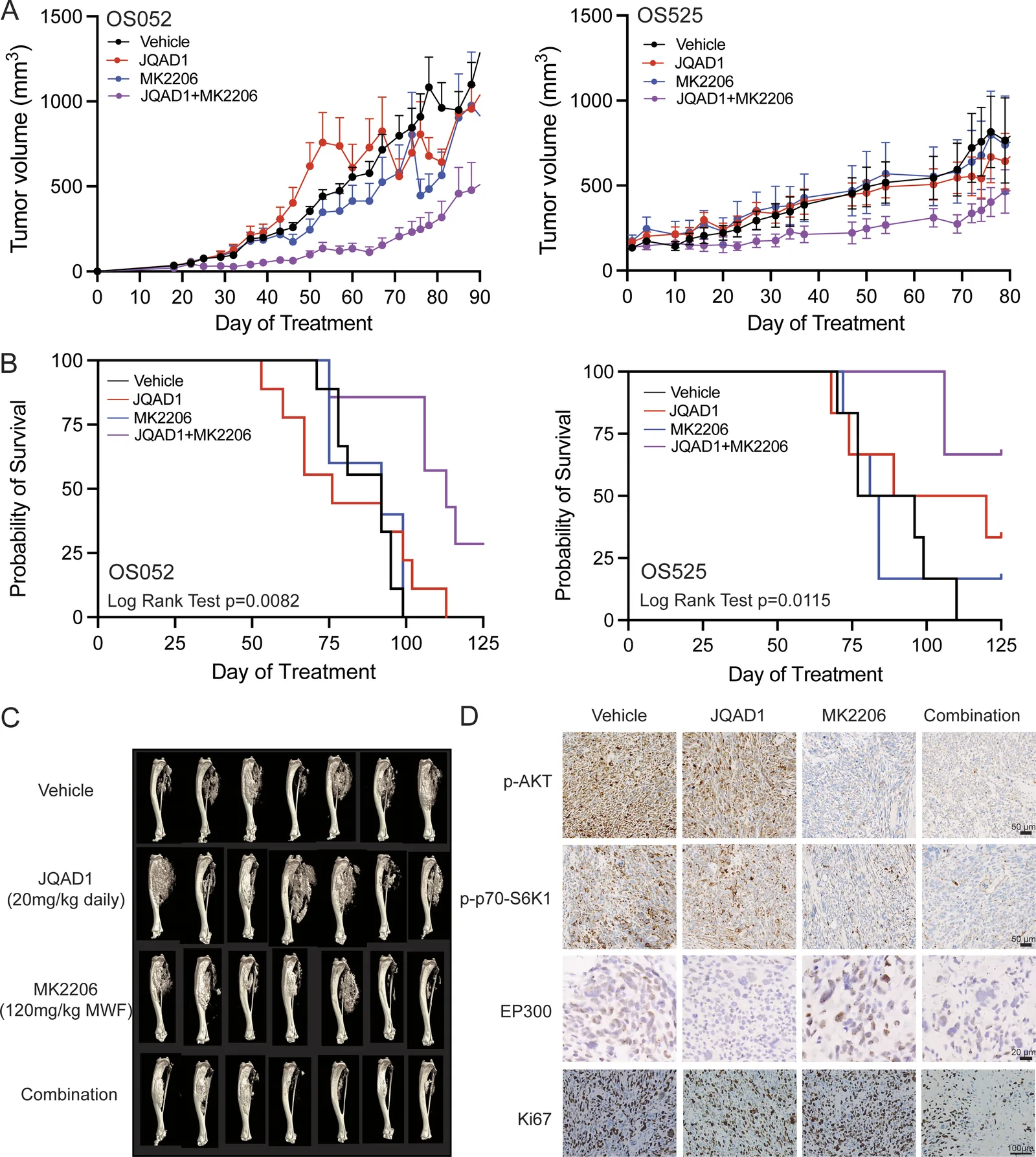
**AKT inhibitors synergize with EP300 degraders in vivo*.* (A)** Orthotopic xenografts of OS052 (left) or OS525 (right) cells were performed, and tumor growth was monitored by caliper measurements. Treatments were vehicle control, JQAD1 (20 mg/kg i.p. daily), MK-2206 (120 mg/kg p.o., three times weekly), or the combination of JQAD1 and MK-2206. OS052 *n* = 9 (vehicle), 9 (JQAD1), 7 (MK-2206), 7 (combination); OS525 *n* = 7 animals/group except for *n* = 6 animals (vehicle). Bars = SD. Combination (JQAD1+MK-2206) grows slower than all other treatments by logistic regression with P < 10^−16^ (OS052) and P < 10^−6^ (OS525). **(B)** Probability of survival to d = 125 in OS052 (left) and OS525 (right) tumor-bearing animals treated with vehicle control, JQAD1, MK-2206, or JQAD1+MK-2206. Combination treatment group displays enhanced survival by the log-rank test (Mantel–Cox test), P = 0.0081 (OS052) and P = 0.0115 (OS525). OS052 *n* = 9 (vehicle), 9 (JQAD1), 9 (MK-2206), 8 (combination); OS525 *n* = 7 animals/group. **(C)** Micro-CT performed at day 27 of treatment in the OS052 tumor model displays reduced tumor-associated ectopic bone volume with combination treatment, compared with vehicle, JQAD1, or MK-2206 alone. Data are representative of all tumor-bearing animals per treatment group. **(D)** Individual representative images showing immunohistochemical staining of OS052 tumor xenografts at day 10 of treatment for phospho-Ser-473-AKT, phospho-Thr-389-p70S6K, EP300, and Ki67. Bar = 50 μm (phospho-Ser-473-AKT, phospho-Thr-389-p70S6K), 20 μm (EP300), 100 μm (Ki67). Images are representative of *n* = 5 tumors subjected to immunohistochemical staining with each antibody, per group.

**Figure S5.**
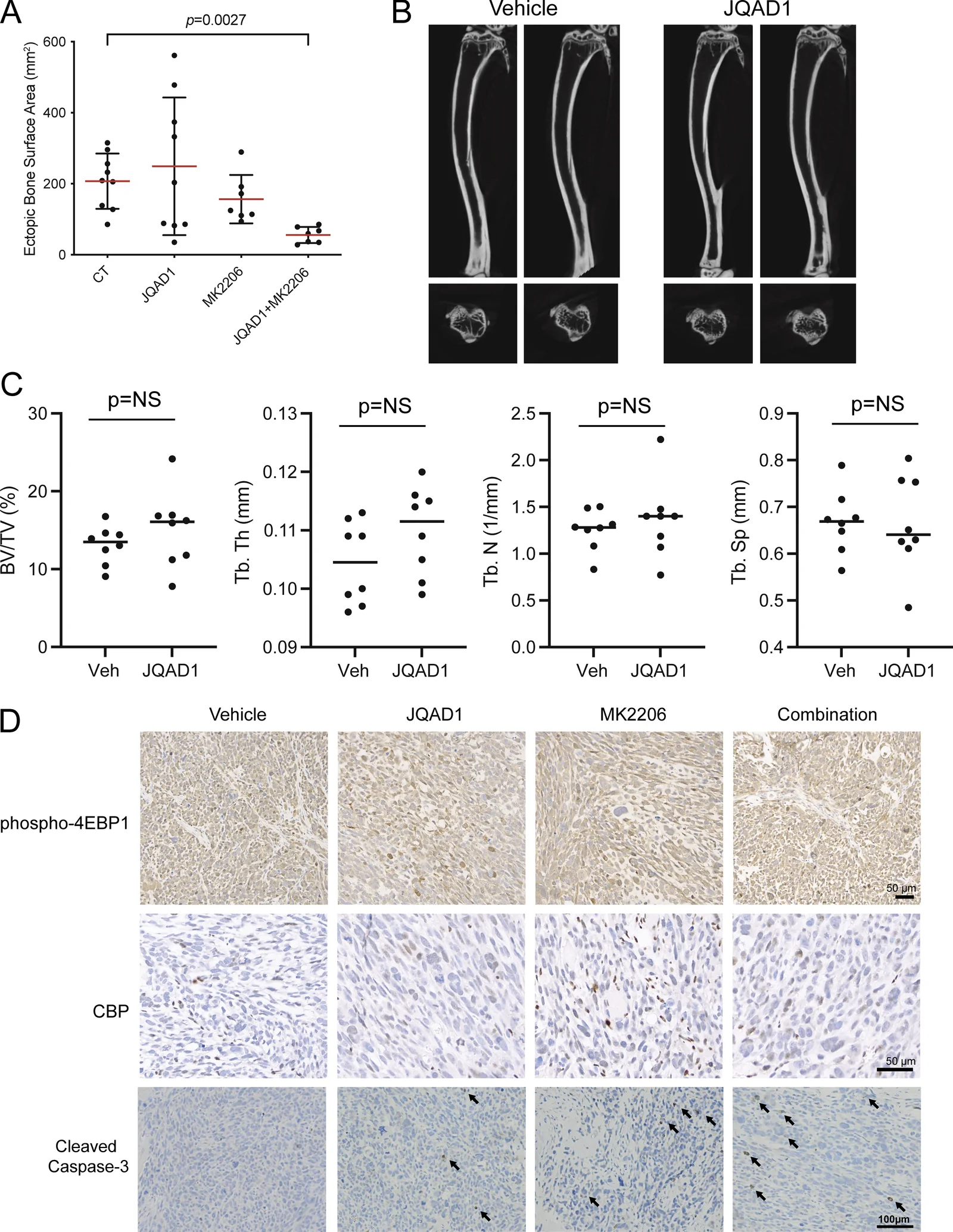
**JQAD1 has limited effects on bone properties and expression of CBP or phosphorylated 4EBP1 in vivo*.* (A) **Micro-CT quantification of ectopic bone surface area of OS052 tumor xenografts at day 27 of treatment with vehicle (*n* = 9), JQAD1 (*n* = 9), MK-2206 (*n* = 7), or JQAD1+MK-2206 (*n* = 7). P < 0.01 by two-way ANOVA with post hoc Tukey test. Bars represent data spread, and red indicates the median. **(B)** Representative microCT images of non-tumor-bearing tibia from OS052 xenograft study mice. Images taken ex vivo at the end of the study, in animals treated with either vehicle or JQAD1. Images representative of *n* = 8 animals per treatment. **(C)** MicroCT analysis and quantitation of bone parameters in the contralateral, non-tumor–bearing tibia of mice treated with vehicle (veh) or JQAD1 at the end of therapy. Demonstrated are BV/TV (%), trabecular number (Tb.N [per mm]), trabecular thickness (Tb.Th [mm]), and trabecular separation (Tb.Sp [mm]) of the tibia. NS = not significant by two-tailed Students *t* test. All data points shown with bar = median. **(D)** Individual representative images showing immunohistochemical staining of OS052 tumor xenografts at day 10 of treatment for CBP, phosphorylated-Thr37/46-4EBP1, and cleaved caspase-3. Bar = 50 µm (CBP, phospho-4EBP1), 100 µm (cleaved caspase-3). Arrows identify foci of cleaved caspase-3 signal. Images are representative of *n* = 5 tumors subjected to immunohistochemical staining with each antibody, per group.

Treatment of OS052 and OS525 tumor xenograft-bearing mice with low-dose MK-2206 or JQAD1 alone had limited effects on tumor growth rates (Fig. 5 A). However, combined low-dose MK-2206 and JQAD1 reduced tumor volumes, caused slower growth rates (Fig. 5 A), and led to a survival benefit (Fig. 5 B). To more precisely measure changes in tumor growth and examine morphologic properties, we performed micro-CT imaging on OS052 tumor–bearing animals after 27 days of treatment. Comparison demonstrated a range of ectopic bone formation, consistent with tumor growth in vehicle control, MK-2206, and JQAD1 monotherapy groups, with significantly reduced ectopic bone formation in combination treatment groups (Fig. 5 C). This was concurrent with a reduction in ectopic bone surface area (Fig. S5 A). Though not statistically significant, since some animals treated with JQAD1 appeared to have elevated ectopic bone formation (Fig. 5 C), we examined the effect of JQAD1 on bone parameters in the contralateral, non-tumor–bearing leg. This revealed no changes in appearance, bone volume/trabecular (BV/TV) volume ratio, trabecular number (Tb.N.), trabecular thickness (Tb.Th.), or trabecular separation (Tb.Sp.) of the contralateral legs in vehicle- or JQAD1-treated animals, indicating a lack of JQAD1 effect on normal bone (Fig. S5, B and C). Separately, 5 NMRI-*nu/nu* mice per group were xenografted with OS052 cells as above, but sacrificed after 10 days of treatment for IHC. Across five tumors per group, we observed that JQAD1 treatment reduced EP300, but not CBP expression (Fig. 5 D, Fig. S5 D). Further, MK-2206 reduced phosphorylated AKT expression and, consistent with our in vitro findings, phospho-Thr389-p70-S6K1, but not phospho-Thr37/46-4EBP1 (Fig. 5 D and Fig. S5 D). Finally, combined JQAD1 and MK-2206 treatments led to suppression of the proliferation marker Ki67, with minor induction of the apoptotic marker cleaved caspase-3 (Fig. 5 D and Fig. S5 D). Thus, even at reduced dosing of JQAD1 with conventional dosing of MK-2206, AKT-dysregulated OS PDX tumors are synergistically inhibited by combination treatment in vivo.

Together, these data demonstrate that combinations of AKT inhibitors with EP300 degraders are broadly additive to synergistic across a range of human cancer cell lines, in a manner associated with enhanced signatures of EMT, PI3K–AKT–mTOR activity, and protein translation. These observations are specifically observed in AKT-dysregulated OS PDX cells in vitro and in vivo.

## Discussion

Despite their high sequence homology in functional domains, the paralogous HATs EP300 and CBP have distinct roles in the control of gene expression in a cell type–specific manner (Kasper et al., 2006; Kung et al., 2000; Ramos et al., 2010; Rebel et al., 2002; Shendy et al., 2024). These observations are not evaluable using available small-molecule inhibitors, since these are nonspecific between EP300 and CBP (Zhou and Cole, 2025). Despite this dual specificity for EP300 and CBP, small-molecule inhibition of EP300 and CBP has been extremely informative, yielding a higher layer of understanding of the functions of distinct domains of these proteins, which, when inhibited, can elicit disparate cellular phenotypes (Shendy et al., 2024; Zucconi et al., 2019). These tools have been useful to elucidate disparate roles of BRD-based scaffolding and catalytic activities of EP300 and CBP (Narita et al., 2021; Weinert et al., 2018). With the development of selective and nonselective degraders of EP300 (Chen et al., 2024; Durbin et al., 2022; Marsh et al., 2025; Vannam et al., 2021), temporally based and specific control of either EP300 or CBP is possible. These studies have demonstrated prominent and cell type–specific effects on control of oncogenic transcription by EP300 in specific contexts, as compared with CBP (Durbin et al., 2022). Importantly, EP300 degradation causes loss of both its catalytic activity that is required for assembly of the transcriptional preinitiation complex and pause release (Narita et al., 2021), and BRD-based scaffolding activities, required for docking of EP300 to acetylated proteins (Welti et al., 2021), among others. The effects of degradation are, in some cases, greater than small-molecule inhibition (Durbin et al., 2022; Mares et al., 2020), and with the clinical advancement of targeted protein degradation agents (Chirnomas et al., 2023), these observations make JQAD1 and other molecules like it with attractive toxicity profiles an intriguing modality for investigation, especially in high-risk, aggressive cancers.

Here, we demonstrate that cell lines representing specific cancer subtypes display enhanced genetic dependency on EP300, as compared with CBP, and vice versa. These data reflect prior data demonstrating synthetic lethality on EP300 in CBP-mutant cancers (Ogiwara et al., 2016), though mutation in EP300 or CBP is not required for these effects, as in high-risk neuroblastoma cell lines which are wild-type for EP300 and CBP, and yet display selective dependency on EP300 (Durbin et al., 2022). Here, we extend these observations to cell lines representing multiple other cancer types, uncovering a predictable range of tumor models with enhanced dependency on EP300 or CBP for study. We identified cell lines from OS as a subtype that is highly sensitive to EP300 genetic knockout. Coupled with cell line degrader testing, we identified PDX-derived OS cell lines with enhanced dependency on EP300, linked by common dysregulation of the AKT signaling axis. OS is a challenging-to-manage bone tumor with no currently approved targeted therapies (Marina et al., 2016; Meltzer and Helman, 2021). Patients with metastatic OS have exceptionally poor survival (Meltzer and Helman, 2021) and are often characterized by either amplifications of c-*MYC* or dysregulation of AKT signaling, driven by loss of *PTEN*, mutation of *PIK3CA*, or amplification of upstream growth factor receptors or *AKT1-3* (Feng et al., 2020; Gong et al., 2017; Marinoff et al., 2023; Perry et al., 2014; Zhou et al., 2019). This selective efficacy of the EP300 degrader JQAD1 in suppressing the growth of AKT-dysregulated OS cells mechanistically requires AKT-driven relocalization of EP300 and CBP, resulting in rewiring of transcription to drive genetic dependency expression.

Our studies provide two new key insights. First, AKT-dysregulated OS respond to EP300 degradation in vitro and in vivo, but non-AKT–dysregulated cell lines do not. These data provide a foundation for exploring the role of EP300 degraders in AKT–dysregulated OS, which is both genetically defined and high-risk, and represents up to an estimated 25% of OS tumors (Fig. 1 L) (Gong et al., 2017; Zhou et al., 2019). Potential caveats to this are our observation of variable sizes of JQAD1-only treated tumors in vivo, though these findings were not statistically significant*.* We observed three mechanisms of AKT dysregulation: (1) amplification of *AKT* genes; (2) deletions of *PTEN*; and (3) co-amplifications of *PDGFRA*, *KIT*, and *KDR* with or without amplification of *VEGFA.* Whether each is similarly responsive to JQAD1 is an area of active exploration. Second, combinations of the AKT inhibitor MK-2206 with JQAD1 are synergistic in AKT-dysregulated but not in c-*MYC*–amplified, OS cells. These agents demonstrate positive interactions widely across cell lines from different cancer types, connected by their dysregulation of AKT pathways. Many tumors display mutational activation of the AKT axis, commonly through hotspot mutations in *PIK3CA*, deletion or mutations of *PTEN*, or mutation/amplification of *AKT* genes (Millis et al., 2016; Zhang et al., 2017). While some OS PDX cell lines and tumors display genetic evidence of both *c-MYC* amplification and AKT dysregulation (Fig. 1, E, G, and L; and Fig. S1 L), in general, there is limited overlap between c-*MYC* gene amplifications or activation of the c-MYC proximal network and AKT pathway dysregulation across cancers (Schaub et al., 2018). These data point to important differences and additional layers of complexity when considering the role of these mutations in context-specific tumors, such as OS, compared with landscapes of tumor genetics. Despite these differences, these data indicate that AKT dysregulation may be a state in which combinations of AKT inhibitors and EP300 degraders demonstrate positive interactions. These data echo prior analyses in neuroblastoma demonstrating that genetic loss of *PIK3CA* enhances the effects of JQAD1 (Lee et al., 2023) and further draw distinctions between c-MYC–associated and AKT-driven pathways, both of which are associated with enhanced OS tumor growth and poor patient survival (Feng et al., 2020; Gong et al., 2017; Marinoff et al., 2023; Zhou et al., 2019). With the recent clinical approval of AKT inhibitors (Dilawari et al., 2024), these observations may suggest a more general strategy to enhance the effects of either EP300 degraders or AKT inhibitors in specific genetic contexts.

The activity of JQAD1 is dependent on expression of the E3 ubiquitin ligase receptor *CRBN* (Durbin et al., 2022). Here, we confirm these results, but now extend these to identify a critical role, across cancer cell lines, of protein synthesis in the efficiency of JQAD1. This goes to explain the prior observation of *PIK3CA* knockout causing sensitization to JQAD1 in neuroblastoma cell lines (Lee et al., 2023), but now extends this finding across cells from a myriad number of cancer types and suggests a potentially generalizable mechanism for enhancing the activity of PROTACs by slowing protein resynthesis rates. These data also highlight a seeming paradox, however: in OS cells, AKT-dysregulated activity is required for JQAD1 function, while across a variety of other cancer cell lines, suppression of AKT using inhibitors positively interacts with JQAD1. These findings may be partially harmonized by our observation of relatively reduced protein synthetic rates in AKT-dysregulated OS cells, compared with *c-MYC–*amplified cells. AKT is a central signaling node in human cancer cells that drives a myriad of pathways downstream (Vasan and Cantley, 2022), including direct phosphorylation of EP300 to promote transcriptional coactivation (Huang and Chen, 2005). Our analysis implicates roles for AKT-directed EP300, CBP, and H3K27ac localization, in addition to protein synthesis downstream of AKT, as being critical for these interactions. Whether additional pathways downstream of AKT are implicated in these responses represents the target of ongoing work in OS cells.

In summary, we identify that OS models harboring dysregulation of the AKT pathway are genetically and pharmacologically sensitive to loss of the EP300 HAT, mediated by AKT-driven relocalization of EP300 proteins to drive the transcription of genetic dependencies in these cells. These findings suggest that AKT signaling promotes a downstream transcriptional program that may be shaped by epigenetic enzymes to drive genes responsible for lineage-specific cancer cell growth. We extend these observations to demonstrate synergy between AKT inhibitors and EP300 degraders across human cancer cell lines, identifying that transcriptional signatures of elevated protein translation are associated with enhanced combinatorial effects. These findings support the continued investigation of EP300 degraders for use in AKT-dysregulated OS, and suggest that combinations of EP300 degraders and AKT inhibitors may be of interest in other challenging-to-manage cancer settings.

## Materials and methods

### Cell lines and reagents

OS052, OS525, OS152, OS186, OS526, OS742, OS384, and OS457 OS PDX cell lines were obtained from Dr. Alejandro Sweet-Cordero (University of California, San Francisco, San Francisco, CA, USA). S2 cells were a gift from Dr. Karen Adelman (Harvard Medical School, Boston, MA, USA). OS252 cells were provided by Dr. Lily Guenther (St. Jude Children’s Research Hospital, Memphis, TN, USA). U2OS, SJSA1, and 143B cells were obtained from ATCC, and CAL72 was obtained from DSMZ. All cell lines were short tandem repeat (STR) tested for identity. Most human cell lines were cultured in DMEM medium containing 10% heat-inactivated fetal calf serum and 1% penicillin-streptomycin-glutamine. CAL72 was cultured in DMEM containing 10% heat-inactivated FBS, 2 mM L-glutamine, and 1× insulin-transferrin-sodium selenite. 143B cells were cultured in EMEM+10% heat-inactivated FBS + 0.015 mg/ml 5-bromo-2′-deoxyuridine. SJSA1 was cultured in RPMI+10% heat-inactivated FBS. U2OS was cultured in McCoy’s 5A media+10% heat-inactivated FBS. S2 cells were cultured in Schneider’s medium containing 10% heat-inactivated FBS and 1% penicillin-streptomycin. All media and components were obtained from Gibco. All cell lines were validated to be free of *Mycoplasma* species by routine testing. All cells were cultured in a humidified environment at 37°C, 5% CO_2_. JQAD1, A485, and CCS1477 were all synthesized in the Qi laboratory (Dana-Farber Cancer Institute). Pomalidomide was sourced from Combi-Blocks Inc. MK-2206 for in vitro use was obtained from Selleck Chemicals, NC0709830, and for in vivo use was synthesized by the Qi Laboratory (Dana-Farber Cancer Institute).

### Stable cell line preparation

Myr-AKT, c-MYC, and control vectors were purchased from https://www.addgene.org/ (pCDH-puro-cMYC, plasmid #46970; pCDH-puro-myr-HA-AKT1, plasmid #46969; control vector pCDH-CMV #72265). The packaging plasmid (psPAX2), envelope plasmid (pMD2.G), and LentiCas9-blast were obtained from https://www.addgene.org/. Plasmid IDs were: psPAX2 (#12260), pMD2.G (#12259), and LentiCas9 (#52962). Lentiviral particles were produced in HEK293T cells using TransIT-293 (Mirus Bio) according to the manufacturer’s protocol. Viral supernatants were harvested after 48 and 72 h of transfection and concentrated using Lenti-X Concentrator reagent (Takara Bio Inc.), according to the manufacturer’s instructions. Cell lines were infected with the individual lentivirus particles using polybrene at a final concentration of 1 µg/ml. All cells were at 80% confluency at the time of transfection. Cells were selected using 5 µg/ml puromycin or 1 μg/ml blasticidin and validated for expression of the target by western blotting.

### CRISPR-Cas9–based knockout

SgRNAs sequences targeting *EP300* (EP300-1, 2), *CBP* (CBP-1, 2), *LACZ*, or a gene desert on chromosome 2 (ch2.2) were previously described (Durbin et al., 2022). These sequences were cloned into pLKO.5-puromycin (#57825; Addgene). Lentiviruses containing these sgRNAs were prepared and used to infect OS052, OS525, and OS186-Cas9–containing cells. Cells were selected with 1 µg/ml puromycin for 2 days prior to replating for colony formation assays or western blotting, as appropriate.

### Cell cycle analysis

OS052 and OS525 cells were harvested and washed in PBS. Single-cell suspensions were obtained. One million cells per sample were resuspended in 1 ml of hypotonic PI solution containing RNase A added immediately before use (0.05 mg/ml propidium iodide, 0.1% sodium citrate, 0.1% Triton X-100, and 0.2 mg/ml ribonuclease A in PBS). Cells were stained for 30 min at room temperature in the dark. Samples were stored on ice, in the dark, and were filtered through a 40-μm mesh strainer immediately before analysis. Cells were analyzed on a BD LSRFortessa Cell Analyzer (BD Biosciences). Flow cytometry analysis was collected in BD FACSDIVA version 9.0. Data analysis was performed in ModFit LT (version 6.0).

### Nascent protein synthesis analysis

OS052, OS525, OS186, and OS152 cells were labelled with 1ug/ml puromycin in growth media for 20 min in a humidified environment at 37 °C, 5% CO_2_. Cells were washed twice with 1× PBS prior to lysis using radioimmunoprecipitation assay buffer (Pierce Biosciences) containing 1× phosphatase and protease inhibitors (Invitrogen). Lysates were processed for western blotting as below.

### Western blotting

Cells growing in culture were lysed for whole-cell lysates as previously described (Durbin et al., 2022; Shendy et al., 2024). Nuclear and cytoplasmic lysates were prepared using the NE-PER nuclear lysate kit (Thermo Fisher Scientific), according to the manufacturer’s protocol. Briefly, equivalent amounts of protein were resolved by western blotting using 4–12% Bis-Tris NuPAGE gels (Thermo Fisher Scientific), or 4–20% TGX stain-free Bio-Rad gels prior to transfer and immunoblotting using primary antibodies to puromycin (1:1,000, Ab315887; Abcam), c-MYC (1:1,000, 5605S; Cell Signaling Technology), total H3 (1:1,000, 04928MI; Millipore), EP300 (1:1,000, 70088S; Cell Signaling Technology), CBP (1:500, 7389S; Cell Signaling Technology), AKT1 (1:1,000, 9272S; Cell Signaling Technology), cleaved-PARP1 (1:1,000, 9542S; Cell Signaling Technology), p(Ser473)AKT (1:1,000, 4060T; Cell Signaling Technology), p(T389)P70S6K (1:1,000, 9234T; Cell Signaling Technology), Total P-70S6K (1:1,000, 2708T; Cell Signaling Technology), p-S6 (s235/236) (1:1,000, 4858T; Cell Signaling Technology), p-S6 (s240/244) (1:1,000, 5364T; Cell Signaling Technology), Total S6 (1:1,000, 2217S; Cell Signaling Technology ), p(Ser209)EIF4E (1:1,000, 9741T; Cell Signaling Technology), Total EIF4E (1:1,000, 9742S; Cell Signaling Technology), p(T37/46)-4E-BP1 (1:1,000, 2855T; Cell Signaling Technology), Total 4E-BP1 (1:1,000, 9452S; Cell Signaling Technology) PTEN (1:1,000, 9552; Cell Signaling Technology), β-actin (1:1,000, 4967S; Cell Signaling Technology), vinculin (1:1,000, MAB3574; Millipore), Lamin B1 (1:1,000, ab16048; Abcam), and CRBN (1:1,000, 71810S; Cell Signaling Technology). Secondary antibodies were HRP-conjugated anti-rabbit or anti-mouse (1:2,500, Thermo Fisher Scientific), incubated prior to exposure to enhanced chemiluminescence reagents (Bio-Rad Clarity Max Western ECL Substrate), or anti-rabbit IR-680 or anti-mouse IR-800 (1:5,000), and imaged on a LiCOR Odyssey XF imager (LiCOR Biosciences). Densitometry was performed on western blots using either LiCOR Odyssey or ImageJ (NIH).

### Cell growth and differentiation assays

CellTiter-Glo assay was performed as per the manufacturer’s instructions (Promega). Briefly, 1,000 cells/well were plated into 384-well plates and treated with a range of compound doses using a TECAN D300e robotic-assisted compound dispenser (TECAN Ltd). Cell viability was measured at the noted time points, based on luminescence by the CellTiter-Glo assay (Promega) and read on an Envision 2104 (PerkinElmer), according to the manufacturer’s protocol. For colony formation assays, cells were treated with sgRNAs as detailed. Cells were trypsinized, resuspended, and counted, with 1,000 cells/well plated in quadruplicate in 24-W plates. For compound treatment assays, cells were treated at the start of replating. Cells were grown for 14 days prior to fixation in ice-cold 100% methanol and staining with crystal violet solution. For assessment of differentiation, cells were cultured for 18 days in the presence of osteoblastic differentiation medium (10 nM vitamin D3, 100 nM dexamethasone, 50 µg/ml sodium L-ascorbate, and 10 mM β-glycerophosphate) and treated with DMSO or JQAD1 at indicated concentrations. Mineralization was revealed by Alizarin red (40 mM, pH 4.2) staining after fixation with 70% ethanol. For cell viability, cells were grown for 10 days in the presence of JQAD1 or DMSO treatment, prior to fixation in 1% glutaraldehyde and staining with crystal violet solution.

### PRISM combination analysis

#### PRISM assay overview

PRISM assay has been described previously (Durbin et al., 2022; Shendy et al., 2024). For PRISM combination testing, in brief, the drug combination was screened in triplicate across ∼900 PRISM barcoded cancer cell lines for 5 days (Corsello et al., 2020) at the doses specified in Fig. 4 C. Cell lines found in each screen are detailed in Table S9. All cell lines were cultured in RPMI 1640 with 10% FBS. Cell lines were lysed with Qiagen TCL buffer to isolate mRNA. mRNA was then reverse-transcribed into cDNA, and then the sequence containing the unique PRISM barcode was amplified using PCR. Finally, Luminex beads that recognize the reverse complement of the specific barcode sequences in the cell set were hybridized to the PCR products. The magnetic beads attached to the PCR products were then detected using a Luminex FLEXMAP 3D scanner, which reports signal as a median fluorescent intensity (MFI). The MFI signal was then normalized using the MFI of spiked-in control barcodes, and this normalized MFI was used to calculate the fold-change of a cell line between treatment and negative control (DMSO) as a measure of its viability (V). This viability was then processed to calculate AUC values. Details of these data processing steps are at: https://github.com/cmap/dockerized_mts.

#### Measuring synergy in PRISM data: MSS

To assess drug synergy, we compared the observed viability of the drug combination ($V_{\text{AB}}$) with two commonly used null models based on the single-agent viabilities ($V_{A}$, $V_{B}$). The Bliss model (Bliss) assumes the two drugs act independently, with the model expectation being

$$V_{\text{BLISS}}=V_{a}\times V_{b}.$$

The highest single agent model (HSA) (Mathews Griner et al., 2014) follows the expectation that the combination viability is the smallest of the two single agents:

$$V_{\text{HSA}}=\min\;\left(V_{a},V_{b}\right).$$

Because measured viabilities are capped at 1, the Bliss expectation is always less than or equal to the HSA expectation. The synergy score is computed as the difference between the model expectation and the observed viability—a positive value indicates synergy while a negative value indicates antagonism. For robustness, we combine both model expectations into a single synergy score, which effectively considers the lower viability of the two model expectations when looking for synergy and the higher viability of the two model expectations when looking for antagonism:

$$S=\left\{ \begin{array}{c} V_{\text{HSA}}-V_{ab},\text{if}{\;V}_{\text{HSA}}<V_{ab}\\ 0,\text{if}\;V_{\text{BLISS}}\leq V_{ab}\leq V_{\text{HSA}}\\ V_{\text{BLISS}}-V_{ab},\text{if}{\;V}_{ab}<V_{\text{BLISS}} \end{array}.\right.$$

This synergy score, averaged across combination doses, is the MSS used in analyses. Details on computation of synergy scores are available at https://www.theprismlab.org/publications.

### GSEA of PRISM data

For comparison of the top 100 most synergistic and bottom 100 least synergistic cell lines, cell lines were first ranked by MSS. The top and bottom 100 cell lines were identified, and whole transcriptomes were retrieved from the CCLE at https://www.depmap.org (24Q2 release). Any overlapping cell lines due to duplicate and conflicting measurements were removed. Gene expression values were used as input for two-class comparison GSEA using either the Hallmark or C2 gene sets (H.all.v2025.1.Hs.symbols.gmt or C2.all.v2025.1.Hs.symbols.gmt), using GSEA_4.4.0 software (Broad Institute), using classic enrichment statistics and gene ranking by *t* test settings. For Hallmark analyses of PRISM data, gene sets with FDR q < 0.25 were identified. For C2 gene sets, those with FDR q < 0.0001 and/or FDR q = 0 (as detailed in the text) were used, and limited to gene sets derived from WikiPathways, REACTOME, or KEGG ontologies.

### DepMap and CCMA dependency analysis

Analysis of dependency data was retrieved from the DepMap portal (https://www.depmap.org) using the 24Q2 dataset. Dependency data were extracted as CHRONOS scores for all cell lines (*n* = 1,372) for the two genes EP300 and CBP. Cell lines were annotated to lineages as described at https://www.depmap.org. Specific dependency in OS cell lines was identified by extracting the CHRONOS Score for EP300 or CBP across 27 OS cell lines (NOS1, G292CLONE141B1, OSA1777, OS186, MG63, U2OS, CAL72, SJSA1, NCCOS1X2C1, OS457, OS526, SAOS2, OS384, NCCESOS1C1, OS052, 143B, CCLFPEDS0019T, OS252, HUO3N1, HSOS1, NRHOS1, NRHOS4, NRHOS2, HUO9, NRHGCT1, C337, and 531MII), with a CHRONOS Score of < −0.5 considered genetic dependency. From the 1,372 cell lines in the 24Q2 release, 1,368 cell lines were used after removal of untransformed cells and cell lines found in lineages containing fewer than three cell lines. Lineages (*n* = 53) and number of cell lines (*n* = 1,368) in the 24Q2 DepMap release are Ewing sarcoma (*n* = 32), OS (*n* = 28), esophageal squamous cell carcinoma (*n* = 25), mixed germ cell tumors (*n* = 11), Burkitt lymphoma (*n* = 7), melanoma (*n* = 67), neuroblastoma (*n* = 42), T cell leukemia/lymphoma (*n* = 20), B lymphoblastic leukemia/lymphoma (*n* = 15), diffuse large B cell lymphoma (*n* = 17), small cell lung carcinoma (*n* = 27), prostate adenocarcinoma (*n* = 15), rhabdoid tumor (*n* = 10), high-grade serous ovarian cancer (*n* = 65), non-Hodgkin lymphoma (*n* = 5), non-small cell lung carcinoma (*n* = 100), uveal melanoma (*n* = 9), renal cell carcinoma (*n* = 31), multiple myeloma (*n* = 20), acute myeloid leukemia (*n* = 33), atypical teratoid-rhabdoid tumor (*n* = 11), head and neck squamous cell carcinoma (*n* = 78), diffuse glioma, (*n* = 117), esophagogastric adenocarcinoma (*n* = 68), endometrial carcinoma (*n* = 30), bile duct cancer (*n* = 37), breast carcinoma (*n* = 66), chondrosarcoma (*n* = 4), medulloblastoma (*n* = 12), primitive neuroectodermal tumor (*n* = 4), hepatocellular carcinoma/hepatoblastoma (*n* = 26), Hodgkin lymphoma (*n* = 4), leiomyosarcoma (*n* = 6), chronic myeloid leukemia (*n* = 13), uterine sarcoma (*n* = 4), well-differentiated thyroid carcinoma (*n* = 4), colorectal adenocarcinoma (*n* = 79), synovial sarcoma (*n* = 6), rhabdomyosarcoma (*n* = 15), ampullary carcinoma (*n* = 9), bladder urothelial carcinoma (*n* = 31), cervical adenocarcinoma (*n* = 4), cutaneous squamous cell carcinoma (*n* = 6), liposarcoma (*n* = 12), chronic lymphocytic leukemia (*n* = 5), meningioma (*n* = 4), Merkel cell carcinoma (*n* = 4), malignant peripheral nerve sheath tumor (*n* = 9), undifferentiated pleomorphic sarcoma (*n* = 10), pancreatic adenocarcinoma (*n* = 68), cervical squamous cell carcinoma (*n* = 14), pleural mesothelioma (*n* = 22), and anaplastic thyroid carcinoma (*n* = 8).

For ratio dependency calculations, the number of cell lines in each lineage with dependency (CHRONOS Score < −0.5) was identified. A pseudo-count of 1 was added to each lineage to correct for lineages with no dependent cell lines. The ratio of the number of cell lines dependent on EP300 to those dependent on CBP was then determined.

### Assignment of AKT-dysregulated or MYC-amplified status to OS cell lines and dependency analysis

Common copy number variations (CNVs) and mutations found in OS cell lines and primary tumors were identified from Sayles et al. (2019). This included CNVs in the following genes: *VEGFA*, *PRKDC*, *MAP2K4*, *PDGFRA*, *KIT*, *KDR*, *AKT1*, *PTEN*, *NF1*, *PIK3CA*, *PIK3CB*, *PIK3R1*, *c-MYC*, and *MYCN.* To reflect additional related genes, the copy number and mutational status of *AKT2*, *AKT3*, and *MYCL* were also profiled. Copy number and mutational status of these genes were obtained from the 24Q2 release of the CCLE for all available OS cell lines. Point mutations in the same genes were annotated where present, and copy number changes were considered a loss when log_2_Copy number ≤ 0.7 and an increase when log_2_Copy number ≥ 1.5. Homozygous deletion (log_2_Copy number = 0) was identified for a few samples, and a high copy number increase was considered when log_2_Copy number ≥ 3. All data were collated, and cell lines were assigned a status of “AKT dysregulated,” “c-*MYC*–amplified,” or “unknown” based on the criteria:

1. AKT dysregulated: Presence of copy number increases or hotspot mutations in positive regulators of AKT signaling AND/OR loss of *PTEN*, with or without loss of c-*MYC*, *MYCN*, or *MYCL.* For one case only (OS052), c-*MYC* low copy gain was identified, but balanced by amplification of several AKT-positive regulators (*MAP2K4*, *PRKDC*) and loss of *PTEN.*
2. *c-MYC* amplified: Presence of any mutational activation or amplification (high copy) of c-*MYC*, *MYCL*, or *MYCN* OR in the setting of low copy gain of c-*MYC*, *MYCN*, or *MYCL*, no more than two other low copy gains of AKT signaling genes.
3. Unknown: Cell lines that could not be reliably fit into either of the above categorizations.

From this categorization schema, c-*MYC–*amplified cell lines included U2OS, C337, NRHOS4, NOS1, CAL72, G292CLONEA141B1, MG63, OS186, and HSOS1; AKT-dysregulated cell lines included OS384, OS252, 531MII, HUO9, NRHOS2, NCCESOS1C1, CCLFPEDS0019T, and OS052. Others were deemed unknown. From these cell lines, the CHRONOS Scores for the 405 genes upregulated in JQAD1-sensitive/OS152-Myr-AKT and 429 JQAD1-insensitive/OS152-EV were obtained from the 24Q2 release of the Cancer Dependency Map. Genes were filtered for those with a CHRONOS Score < −0.5, found in >1 cell line, and used for subsequent analyses.

### Synergy analysis of cell lines

Raw 11 × 11 drug combination matrices and corresponding single-agents were imported into R in 384-well format and analyzed using the Combocat analysis platform according to documentation (https://combocat.stjude.org). Data were normalized to the DMSO-treated wells as a function of percentage cell death, where DMSO represented no cell death. Normalized cell death values from single-agent and combination doses were used to determine the Bliss expectation values, from which Bliss synergy was calculated. The code to generate dose-response curves, IC_50_ values, and plotted matrices was obtained from https://github.com/wcwr/combocat.

### Spike-in RNA-seq and data analysis

Total RNA was extracted from OS052, OS525, OS186, OS384, and OS152-vector control or myr-AKT cells using TRIzol reagent (Ambion). Similar extraction was performed for OS525 cells treated for 24 h with DMSO or 1 μM JQAD1. Prior to extraction, exogenous spike-in of synthetic External RNA Controls Consortium (ERCC) RNA controls was added based on cell number (ThermoFisher). Samples were treated with RNase-free DNase I and spin-purified using the Qiagen RNeasy Kit (Qiagen). Purified RNA samples were quantified using the Quant-iT RiboGreen RNA assay (Thermo Fisher Scientific) and quality-checked by the 4200 TapeStation High Sensitivity RNA ScreenTape assay (Agilent) prior to library generation. Libraries were prepared from total RNA with the Illumina Stranded Total RNA Library Prep Kit with ribosomal RNA subtraction according to the manufacturer’s instructions (PN 20040529; Illumina). Sequencing was performed using an Illumina NextSeq 2000 (paired-end, 75-bp reads).

### Bulk RNA-seq analysis

Raw FASTQ sequences were aligned using HISAT2 (version 2.1.0) (Kim et al., 2015) in paired-end mode using default parameters to version hg38 of the human genome to which the sequences of the ERCC synthetic spike-in RNAs (catalog no. 4456740; Thermo Fisher Scientific) had been added as extra pseudochromosomes. Expression per gene was quantified using htseq-count (Anders et al., 2015) with parameters “htseq-count -i gene_id --stranded = reverse -f bam -m intersection-strict,” and version 31 of the canonical GRCh38 gene list from RefSeq to which ERCC coordinates were added. Transcripts per million (TPM) expression was then computed for each gene. The standard TPM-normalization strategy is: normterm = sum of (readcount × readlength/exonlength) across all genes. TPM = readcount × readlength/exonlength × 1e6/normterm. Exon lengths per gene were calculated by collapsing all exons from all transcripts with the same gene name into a single set using bedtools merge. We used per-gene read counts as input for differential expression analysis by DESeq2 with default parameters (Love et al., 2014). ERCC spike-in controls defined the normalization factors (DESeq2 estimateSizeFactors [controlGenes = ERCC]), which were applied to all genes; ERCC features were then excluded, and gene differential expression was computed on the resulting ERCC-normalized counts. Significantly differentially expressed genes had absolute Log_2_FCs ≥ |1| and adjusted P value ≤ 0.05.

### GSEA for bulk RNA-seq of OS cell lines

To refine the gene list for GSEAPreranked (Subramanian et al., 2005), only genes in the top half based on their normalized mean expression values across samples (basemean from DESeq2 analysis) were selected. Log_2_FC derived from the above DESeq2 analysis were then used to rank retained genes. GSEAPreranked analysis was conducted using the “fgsea” and “clusterProfiler” packages in R. The analysis utilized the following specific parameters: TERM2GENE: Hallmark gene sets (MSigDB release h.all.v2023.1.Hs.symbols.gmt) were used as the predefined terms for GSEA. eps: Set to zero to ensure computational stability and prevent any potential division-by-zero errors. nPermSimple: 10,000 permutations; pvalueCutoff: reporting P value cutoff of 1. GSEA results were visualized using the “ggplot2” package.

### ChIP-seq and data analysis

ChIP-seq was performed as previously described (Durbin et al., 2022; Hsu et al., 2022). The following antibodies were used for ChIP: EP300 (ab10485; Abcam), CBP (#7389; Cell Signaling Technology), and H3K27ac (ab4729; ). For each ChIP, 10 μg of antibody was added to 3 ml of sonicated nuclear extract. Illumina sequencing, library construction, and ChIP-seq analysis methods were previously described (Hsu et al., 2022). For visualization of ChIP-seq tracks, TDF files were visualized using the Integrative Genomics Viewer (IGV) genomics browser (version 2.19.4, Broad Institute).

### ChIP-seq analysis

Paired-end ChIP-seq reads were initially aligned to the *Drosophila* S2 cells (*Drosophila melanogaster*) reference genome (version *Drosophila*_*melanogaster*_S2_cells) using Bowtie (version 1.2.2) (Langmead et al., 2009) in paired-end mode with the parameters -l -k 1 --best and --un to retain unmapped reads. The remaining non-*Drosophila* reads were then mapped to the human reference genome (hg38) using Bowtie (version 1.2.2) (Langmead and Salzberg, 2012) with the parameters -l -k 2 -m 2 --best. Transformations of the mapped read files, including Samtools sort -n, Bedtools bamToBed -bedpe, a manual conversion from BEDPE to BED3, and Bedtools bedToBam, were employed to generate BAM files containing ChIP-seq fragments from each aligned read-pair. For visualization purposes, the reference genomes were partitioned into 50-bp windows using Bedtools makewindows, and fragment coverage across these windows was calculated using Bedtools intersect -c. The coverage was normalized per million mapped fragments, then converted into bedGraph format using bedGraphToBigWig, further into wiggle format via bigWigToWig, and ultimately visualized in the IGV (version 2.16.2) (Robinson et al., 2023) browser for human samples aligned to hg38.

To investigate the differences in coverage of promoter regions between promoters in OS052, OS525 (“sensitive”) and OS152, OS186 (“insensitive”) cells (Fig. 3 F) or OS152-EV and OS152-myr-AKT cells (Fig. 3 G), we defined putative promoter regions for each transcript in the canonical GRCh38 gene list as ±2 kb from the TSS. Individual read coverage for promoter regions was quantified using bamToGFF.py (https://github.com/BradnerLab/pipeline) with the parameters “-r -m 1” from BAM files. The log_2_FC were computed in two comparison settings: (1) OS052, OS525 (sensitive) versus OS152, OS186 (insensitive) cells; (2) average of two replicates of OS152-myr-AKT versus average of two replicates of OS152-EV cells. In all comparisons, log_2_FC was calculated as log_2_(numerator + 1) − log_2_(denominator + 1) to stabilize low or zero signals.

To investigate the differences in coverage of enhancer regions between enhancers in OS052, OS525 (sensitive) and OS152, OS186 (insensitive) cells (Fig. S3 A) or OS152-EV and OS152-myr-AKT cells (Fig. S3), we identified regions enriched in reads, i.e., peaks. To identify regions of significant ChIP-seq signal enrichment, MACS1.4 (Zhang et al., 2008) was used for peak calling. Prior to this, paired-end reads overlapping the ENCODE-defined Problematic Regions blacklist for the hg38 genome (Amemiya et al., 2019) were removed from each alignment to avoid artifacts. Peak calling was then performed usingMACS1.4 with the parameters --keep-dup = auto and -p 1e-9.

ChIP-seq peaks for H3K27ac, EP300, and CBP were processed independently for each factor. For each factor, peaks identified in individual samples were merged using Bedtools merge, generating a distinct set of collapsed peaks per factor. To focus exclusively on enhancers, peaks overlapping promoter regions (defined as ±2 kb from the TSS) were excluded using Bedtools subtract, again performed separately for each factor-specific merged peak set. Remaining enhancer peaks were annotated with the nearest gene using Bedtools closest -t first based on the TSS from the canonical GRCh38 gene list. Read coverage over each factor-specific enhancer region was quantified from BAM files using bamToGFF.py with the parameters -r -m 1. The same log_2_FC calculation strategy used for promoters was applied to enhancer signal comparisons and conducted individually for each histone mark for the two comparison settings: (1) OS052, OS525 (sensitive) versus OS152, OS186 (insensitive) cells; (2) average of two replicates of OS152-myr-AKT versus average of two replicates of OS152-EV cells.

### Whole genome sequence samples and analysis

Genomic sequences from OS PDXs (*n* = 27) were obtained from the Childhood Solid Tumor Network (CSTN) (https://cstn.stjude.cloud/) (Stewart et al., 2017). WGS reads were aligned to the GRCh37-lite reference genome using BWA (version 0.7.12-r1039; *aln* algorithm) (Li and Durbin, 2009). Single nucleotide variants (SNVs) were detected using SequencErr (Davis et al., 2021). Small insertions and deletions (indels) were identified using an in-house de novo variant calling pipeline (to be published elsewhere). CNVs were detected by CONSERTING (Chen et al., 2015) and structural variations (SVs) were identified using CREST (Wang et al., 2011). Both small indels and SVs underwent post-processing with SVIndelTyper (Shao et al., 2026). Analyses focused on 21 genes (*PTEN*, *AKT1*, *AKT2*, *AKT3*, *PIK3CA*, *PIK3CB*, *PIK3R1*, *MAP2K4*, *NF1*, *PDGFRA*, *KIT*, *KDR*, *VEGFA*, *PRKDC*, *MYCN*, *MYC*, *MYCL*, *RB1*, *TP53*, *ATRX*, and *DAXX*) frequently mutated in OS across 27 PDX samples. All candidate coding SNVs, indels, SVs, and CNVs were manually curated and confirmed using IGV (Thorvaldsdottir et al., 2013) to ensure variant validity, and intersected with RNA-seq data provided from the CSTN to ensure copy number changes resulted in changes in mRNA expression.

### Panel-based sequencing samples and analysis

Panel-based sequencing results from the AACR GENIE version 19.0 public cohort were accessed through http://cbioportal.org/. Samples were filtered to include only those patients with a diagnosis of OS and available data, including mutational, structural variant, and copy number data. This resulted in a cohort of 331 unique patients with all data types. Samples were then stratified into groups (AKT dysregulated, *MYC-*amplified, mixed, and neither) based on mutational, copy number, and structural variant status of *PTEN*, *AKT1*, *AKT2*, *AKT3*, *PIK3CA*, *PIK3CB*, *PIK3R1*, *MAP2K4*, *NF1*, *PDGFRA*, *KIT*, *KDR*, *VEGFA*, *PRKDC*, *MYCN*, *MYC*, and *MYCL*, as detailed above.

### In vivo studies

Animal care and experimental protocols were approved by the French Ministry of Research and were conducted in accordance with the institutional guidelines of the French Ethical Committee protocol agreement number APAFIS 8405 and under the supervision of authorized investigators. Briefly, 5-wk-old female NMRI-nude mice (Janvier Lab) were injected with 2 × 10^6^ OS052 cells or 1.5 × 10^6^ OS525 cells into the paratibial site, in the muscle close to the tibia after periosteum scraping. When tumors were palpable, the mice were randomized in 4 groups and treated with vehicle, JQAD1 alone (20 mg/kg/day in 10% DMSO + 10% Kolliphor + 80% β-cyclodextrin 10% in distilled water; IP injection), MK-2206 alone (120 mg/kg in 5% DMSO + 40% PEG400 + 5% Tween80 + 50% distilled water, three times weekly; oral gavage), or combination of JQAD1 and MK-2206 at their respective dosing strategies and frequencies. Tumor volume was measured using a caliper three times per week and calculated by the formula: length × width × depth × 0.5. Data points were expressed as average tumor volume ± SD. Mice were treated in survival studies, with sacrifice when tumor volume reached 1,600–1,800 mm^3^. Alive mice hindlimbs were scanned one day before the beginning of the treatment, at day 27 of treatment, and at the endpoint using a high-resolution microCT scanner (Skyscan 1,276, Bruker) using the following scanning parameters: source voltage 50 kV, source current 200 µA, exposure 455 ms, and applied 0.5-mm aluminum filter. Image acquisition for alive mice was performed with a pixel size of 35 µm. Image acquisition for ex vivo tibiae was performed with a pixel size of 17 µm. Back projections were reconstructed using NRecon (Bruker), and CTAn Software (Bruker) was used for segmentation and 3D analysis. Reconstructed 3D images of the tibiae were obtained using CTVox software (Bruker). Tumor ectopic bone formation surrounding the tibia was analyzed, and ectopic bone surface area (B.S.) was determined. 3D morphometric analysis of trabecular bone was performed. The parameters were reported as percent bone volume (BV/TV), denoting the occupancy of trabecular bone in the medullary volume of interest. Additional parameters measured included: 1. Tb.Th., 2. Tb.Sp., and 3. Tb.N. Separately, OS052 cells were xenografted, and *n* = 5 animals were randomized to four groups (vehicle, JQAD1, MK-2206, and combination JQAD1 and MK-2206) as described above. These animals were treated for 10 days prior to sacrifice for tumor extraction for IHC.

### IHC

Patient-derived OS xenograft tumors (*n* = 5/group) were dissected, fixed in 10% neutral buffered formalin, and processed using the HistoCore PEGASUS Tissue Processor (Leica Biosystems). Processed tissues were embedded in paraffin and sectioned at a thickness of 4 µm using a HistoCore AUTOCUT fully automated rotary microtome (Leica Biosystems). Tumor sections were stained with H&E using the HistoCore SPECTRA ST Stainer (Leica Biosystems).

Simplex IHC was performed on sequential slides to detect the targets described in Table 1.

**Table 1.** Antibodies and staining conditions used for immunohistochemistry

Target	Primary antibody and catalog number	Dilution	Incubation time (min)	Staining platform	Antigen retrieval buffer [time in min]	Detection system
CBP	7389; Cell Signaling	1:25	32	VDU	CC1 [32]	OM RB
Cleaved Caspase 3	9664; Cell Signaling	1:750	32	VDU	CC1 [32]	OM RB
Ki67	MAB7617; R&D Systems	1:500	32	VDU	CC1 [32]	OM RB
Phosphorylated Ser473-AKT	4060; Cell Signaling	1:25	32	VDU	CC1 [32]	OM RB
p300	IHC-00028; Bethyl Laboratories	1:250	30	LBR	ER2 [30]	RF-DAB
Phosphorylated Thr389-p70S6 K	SAB 4503957; Millipore Sigma	1:100	32	VDU	CC1 [32]	OM RB
Phosphorylated Thr37/46-4EBP1	2855; Cell Signaling	1:1,000	32	VDU	CC1 [32]	OM RB

VDU, Ventana DISCOVERY ULTRA (Roche Diagnostics Corporation), LBR, Leica Bond Rx (Leica Biosystems), CC1, DISCOVERY Cell Conditioning Solution 1 (catalog no. 06414575001; Roche Diagnostics Corporation, proprietary Tris-based [pH 8.0–9.0] antigen retrieval buffer), ER2, BOND Epitope Retrieval Solution 2 (catalog no. AR9640; Leica Biosystems, proprietary EDTA-based [pH 9.0] antigen retrieval buffer), OM RB, Ventana DISCOVERY OmniMap anti-Rb HRP (RUO) (catalog no. 760-4311; Roche Diagnostics Corporation, proprietary HRP-conjugated secondary detection system for rabbit primary antibodies), RF-DAB, BOND Polymer Refine Detection System (catalog no. DS9800; Leica Biosystems, proprietary polymer secondary detection system with diaminobenzidine [DAB] chromogen and hematoxylin counterstain), Tyramide Signal Amplification was applied for phosphorylated-AKT and p70S6K IHC using the DISCOVERY HQ amplification kit (catalog no. 06472320001; Roche Diagnostic Systems).

H&E and IHC slides were reviewed by a board-certified veterinary anatomic pathologist (AMK) using an Olympus BX46 microscope (Evident Scientific). Representative light microscopic images were acquired using a Spot Insight Color Mosaic microscope camera (Spot Imaging).

### H-scoring of IHC

The modified H-score system used in Fig. 1 K was developed by a board-certified veterinary anatomic pathologist (AMK) on the basis of classic frameworks (McCarty et al., 1985; Remmele and Stegner, 1987), using a three-point intensity and four-point distribution schema. By this metric, a final modified H-score was given by the formula H-score = Intensity (0–3) × Distribution (0–4) for a range of 0–12. Criteria for schema are provided in Table 2.

**Table 2.** Immunohistochemical properties scored in a modified H-Score system

Component	Score	Definition
Intensity	0	No staining above background
	1	Weak; perceptible at high power (20–40×), pale or faint signal
	2	Moderate; definite staining visible at intermediate power (10×)
	3	Strong; maximal expected intensity, readily apparent at low power (2–4×)
Distribution	0	0% positive tumor cells
	1	1–10% positive tumor cells
	2	11–50% positive tumor cells
	3	51–80% positive tumor cells
	4	>80% positive tumor cells

### Statistical analysis

Statistical analyses are detailed for each specific experiment in the relevant figure legends. All experiments represent >3 independent biological replicates, and a P value <0.05 was considered significant. Analyses were performed using GraphPad Prism version 10.0 (GraphPad Software Ltd) unless otherwise stated.

### Online supplemental material

Supplementary material includes five supplemental figures and 10 supplemental tables. Fig. S1 contains supporting data for Fig. 1, including immunohistochemical and genomic sequencing data from all PDX models used and clinical panel-based sequencing of OS patients. Fig. S2 contains supporting data for Fig. 2, including targeted knockout of EP300 and CBP and pomalidomide rescue experiments. Fig. S3 contains supporting data for Fig. 3. Fig. S4 contains supporting data for Fig. 4, including data relating to AKT inhibitor and EP300 degrader synergy studies. Fig. S5 contains supporting data for Fig. 5, including bone parameter data in murine EP300-degrader–treated mice and additional immunohistochemical stains. Tables S1 and S2 contain EP300 degrader PRISM treatment data. Table S3 contains mutational data from OS PDX samples. Table S4 contains differentially expressed gene lists in EP300-degrader–sensitive and myr-AKT–expressing OS152 cells, compared with insensitive or EV-expressing OS152 cells. Tables S5, S6, and S7 contain copy number, mutational data, and synthesis of OS cell line genetic subtypes in the CCLE. Table S8 contains statistical significance values for all ChIP-seq comparisons. Table S9 contains MMSs for all cell lines treated with either JQAD1 and MK-2206 or JQAD1 and capivasertib, by PRISM screening. Table S10 contains enriched REACTOME, KEGG, and WikiPathways results from analysis of PRISM combination experiments.

## Supplementary Material

Table S1 shows the effect of JQAD1 in 544 cell lines by PRISM analysis.

Table S2 shows the effect of JQAD1 in the top 6 lineages by PRISM analysis.

Table S3 shows WGS analysis of 27 OS PDXs.

Table S4 shows common up- and downregulated genes in JQAD1-sensitive and -insensitive and myr-AKT/EV–driven OS cells.

Table S5 shows CNVs identified in OS DepMap cell lines.

Table S6 shows mutations identified in 24Q2 OS DepMap cell lines with dependency data.

Table S7 shows OS 24Q2 DepMap cell line annotations for cells with dependency data.

Table S8 shows statistics for genome-wide ChIP-binding comparisons.

Table S9 shows combination JQAD1-AKT inhibitor PRISM screens.

Table S10 shows enriched C2 MSigDB pathways in top and bottom JQAD1+AKT inhibitor synergistic cells.

SourceData F2 is the source file for Fig. 2.

SourceData F4 is the source file for Fig. 4.

SourceData FS1 is the source file for Fig. S1.

SourceData FS2 is the source file for Fig. S2.

SourceData FS4 is the source file for Fig. S4.

## Data Availability

RNA-seq and ChIP-seq data generated in this study have been deposited in the Gene Expression Omnibus database under SuperSeries accession number GSE305938. Whole-genome sequences from PDX samples are available from https://cstn.stjude.cloud/. Panel-based sequencing of human OS samples is available through AACR GENIE, via https://www.cbioportal.org.
