# Supplementary material for: Dysregulated AKT signaling reprograms osteosarcoma to drive selective reliance on EP300
Source: J Exp Med. 2026 Oct 20;223(11):e20251898. doi: 10.1084/jem.20251898 (PMC13682445; doi:10.1084/jem.20251898)

SourceDataF2B.pdf

OS052 EP300

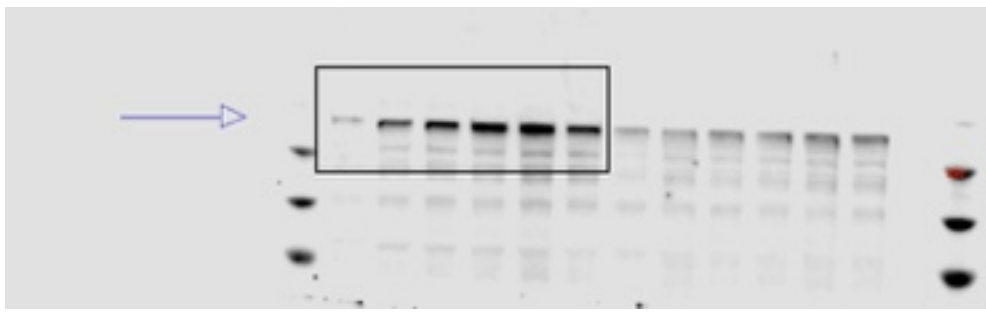

OS052 CBP

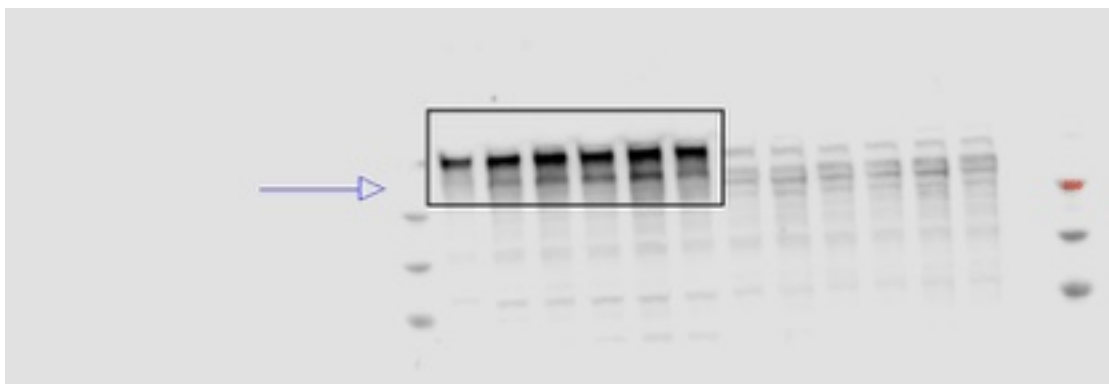

OS052 MYC

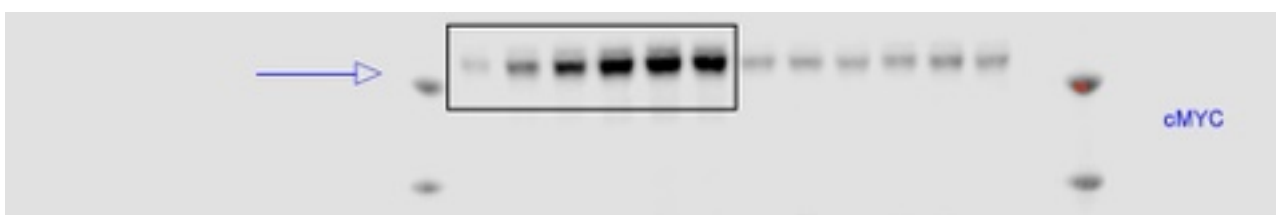

OS525 EP300

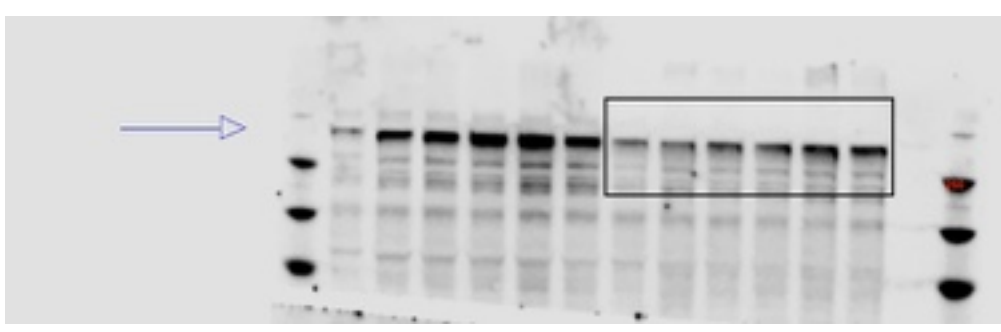

OS525 CBP

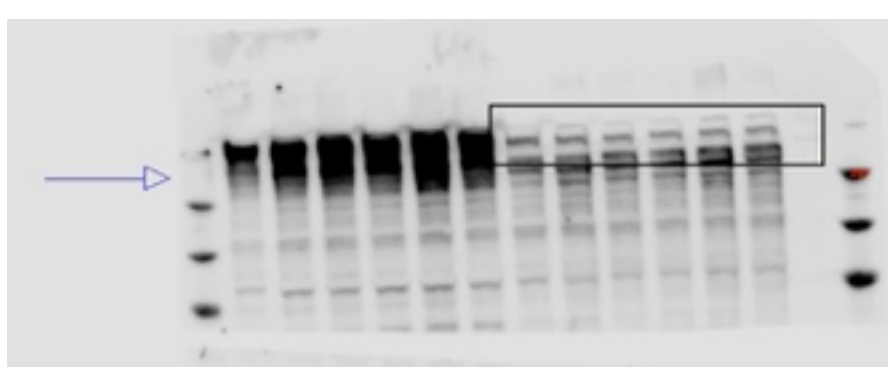

OS525 MYC

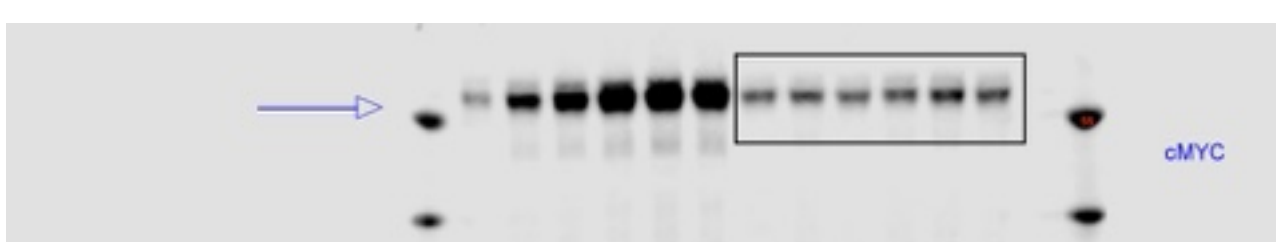

Both lines - PARP1

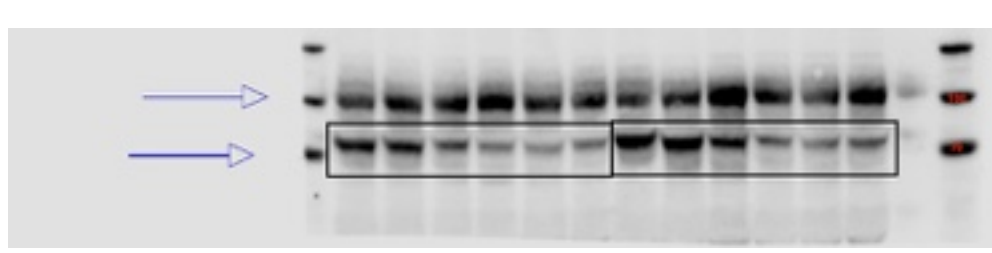

Both lines - lamin

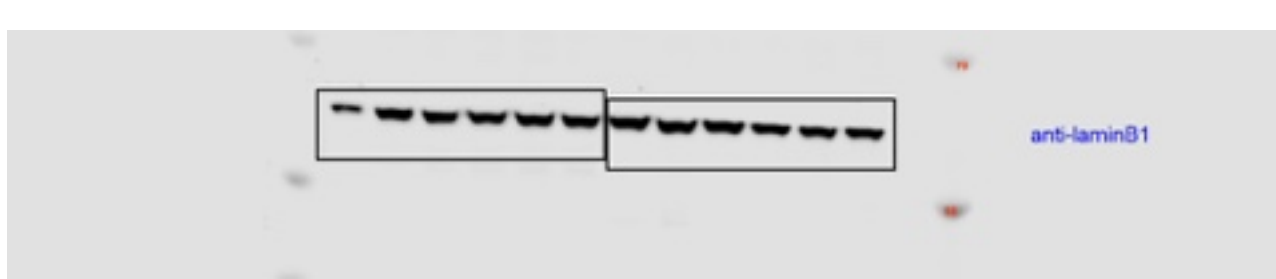

Both lines - Actin

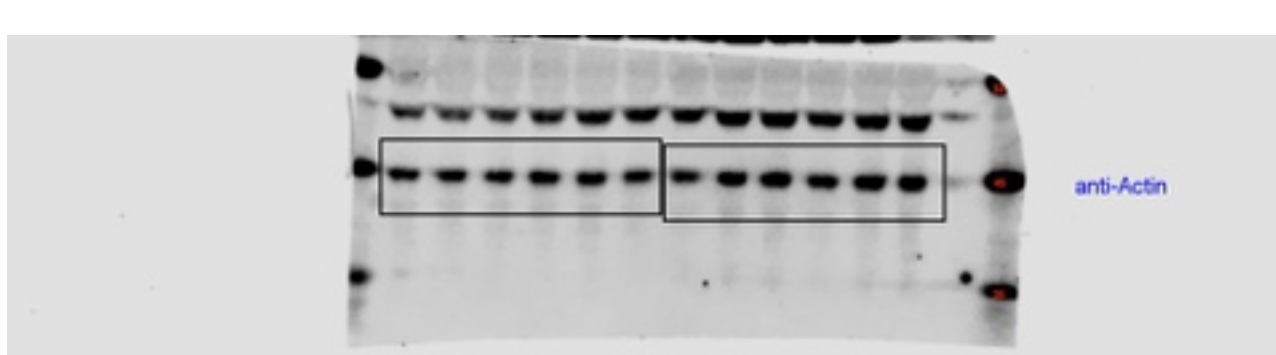

SourceDataF2C.pdf

EP300

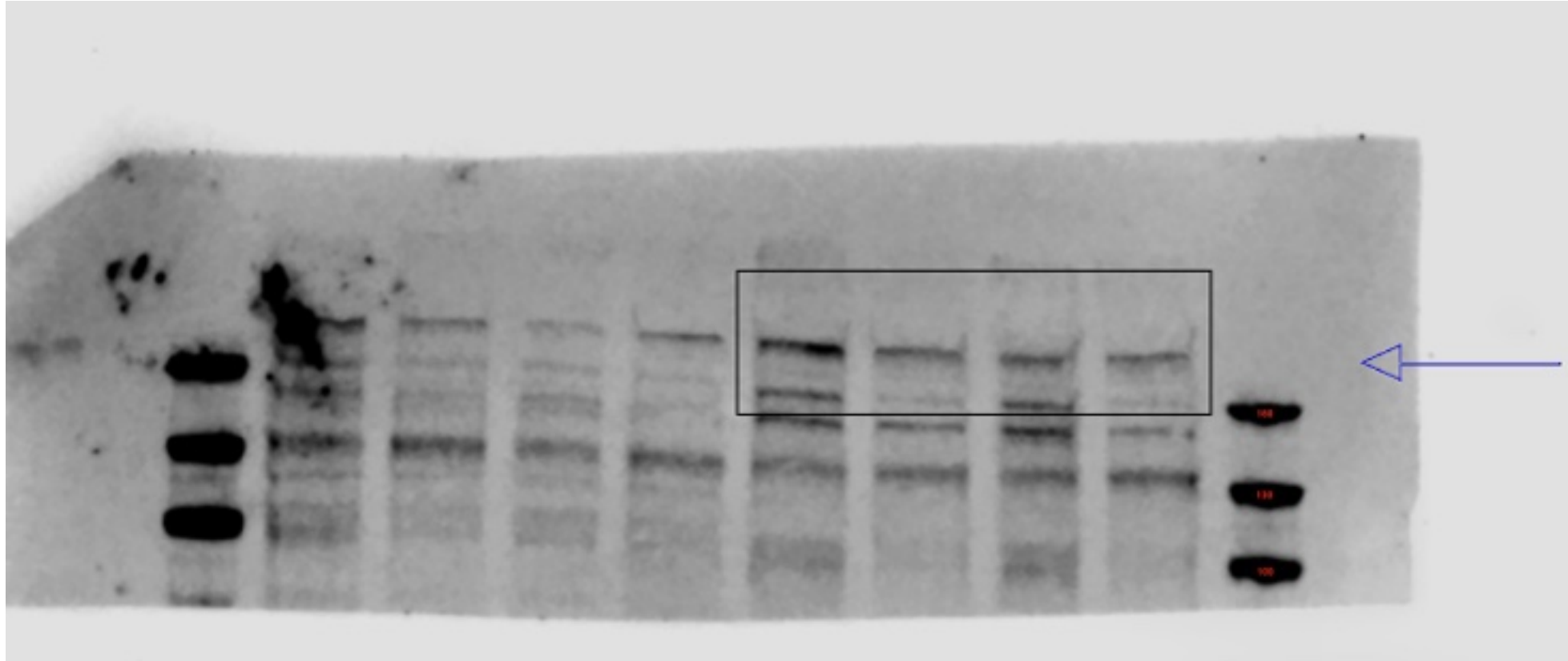

MYC

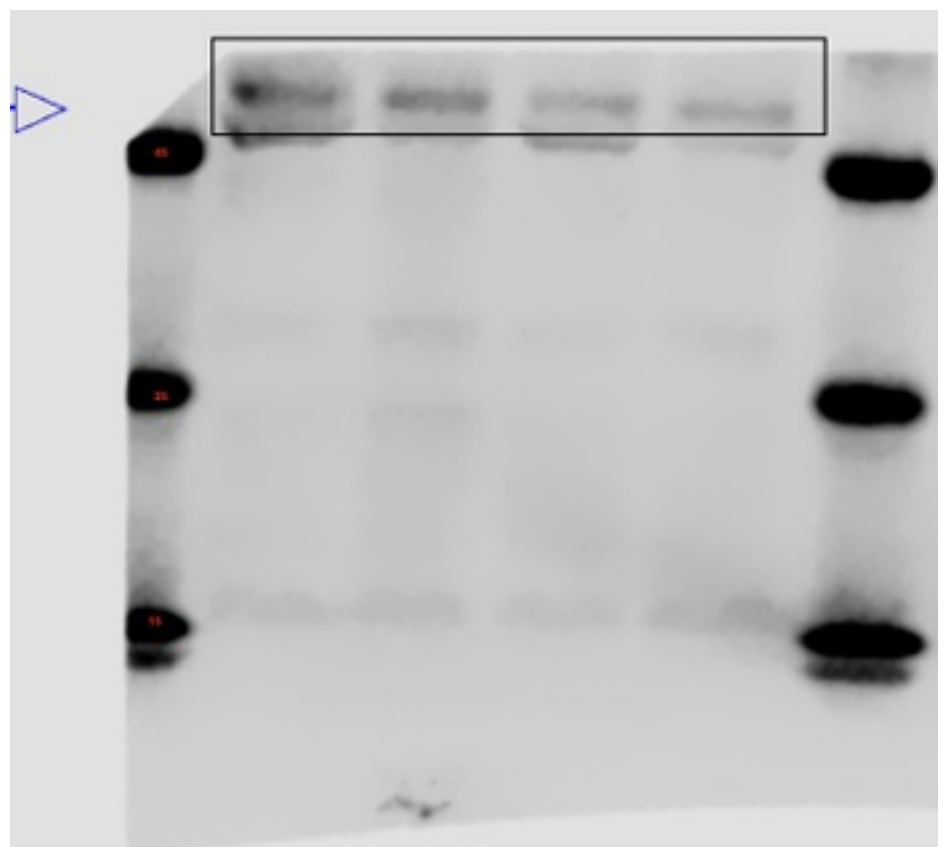

PARP

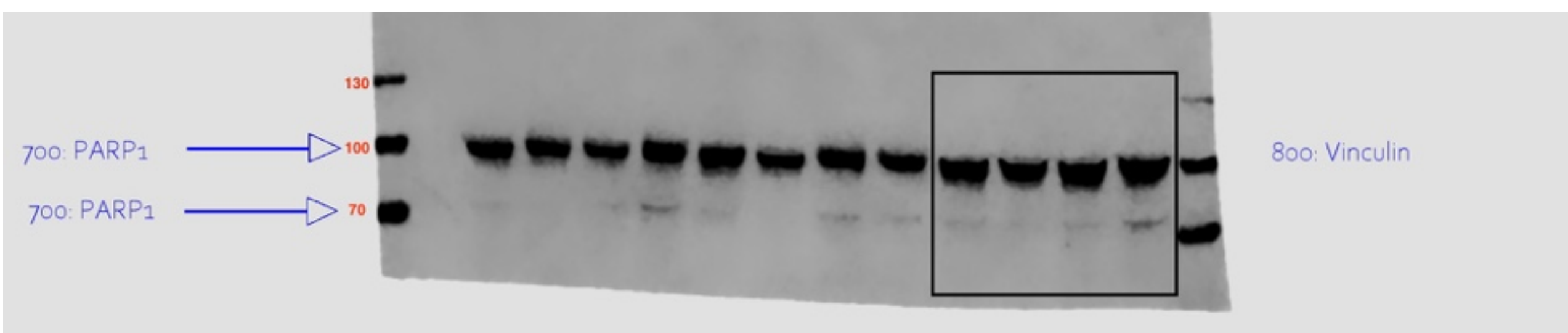

Vinculin

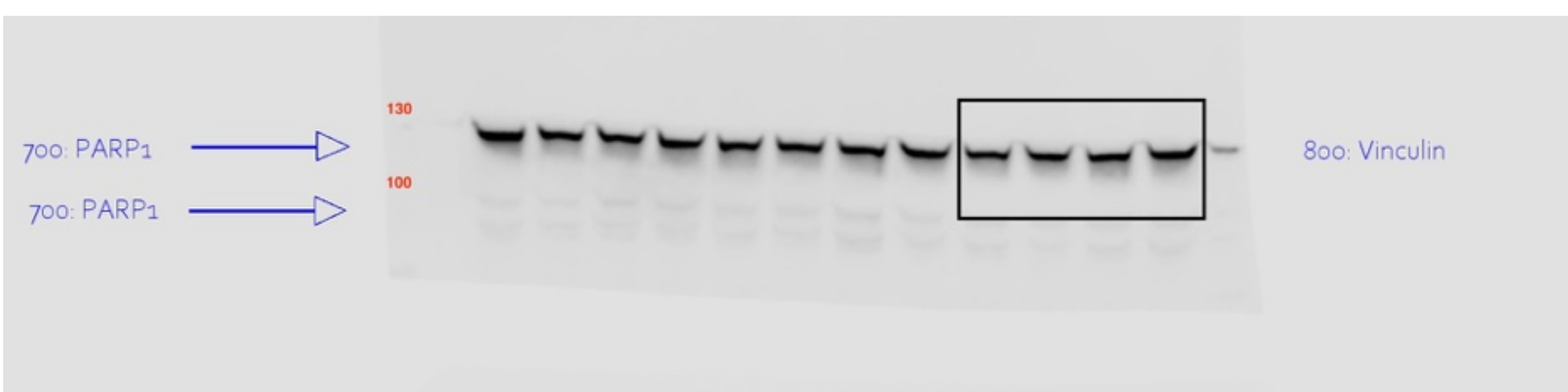

SourceDataF2D.pdf

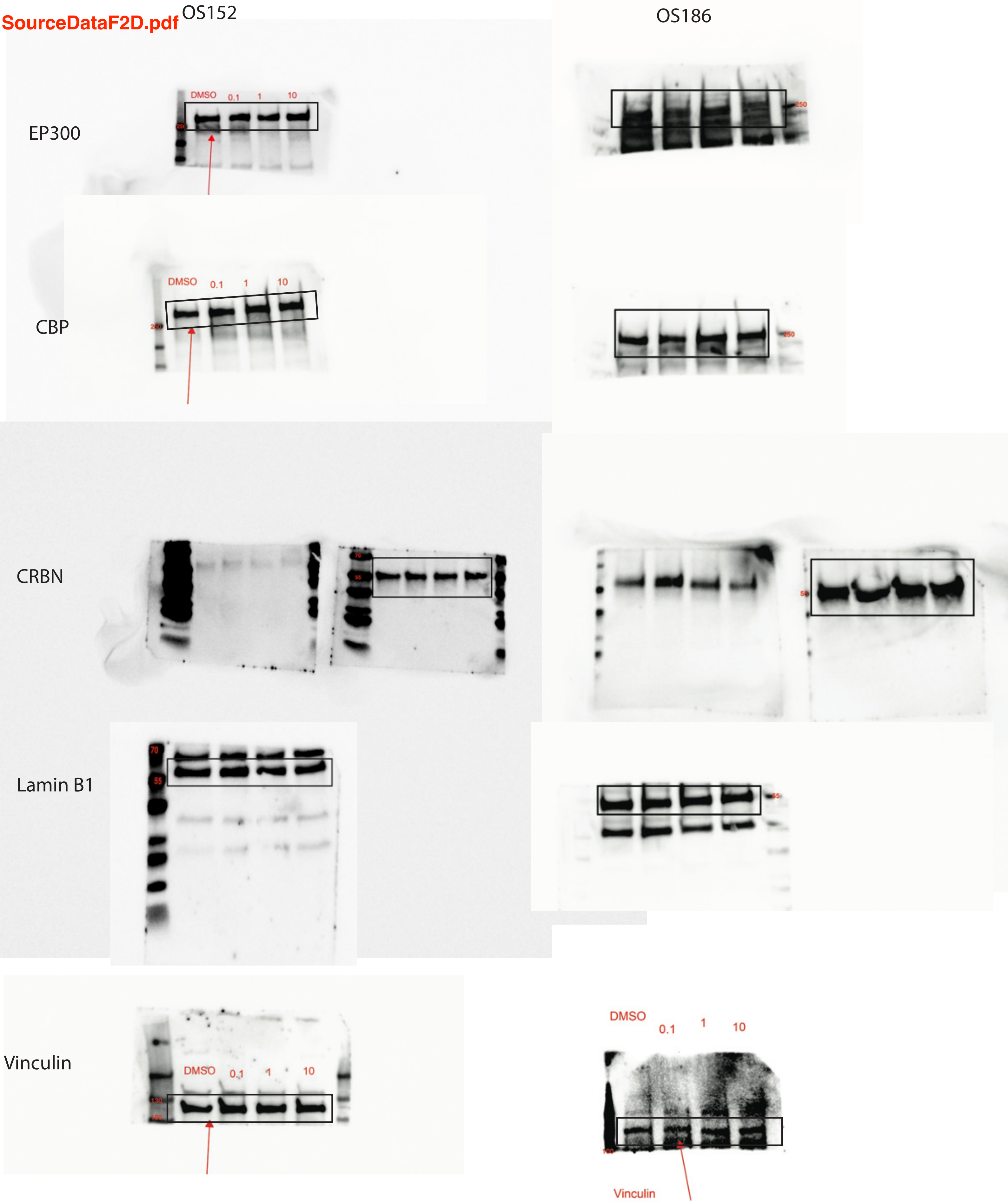

SourceDataF2F.pdf

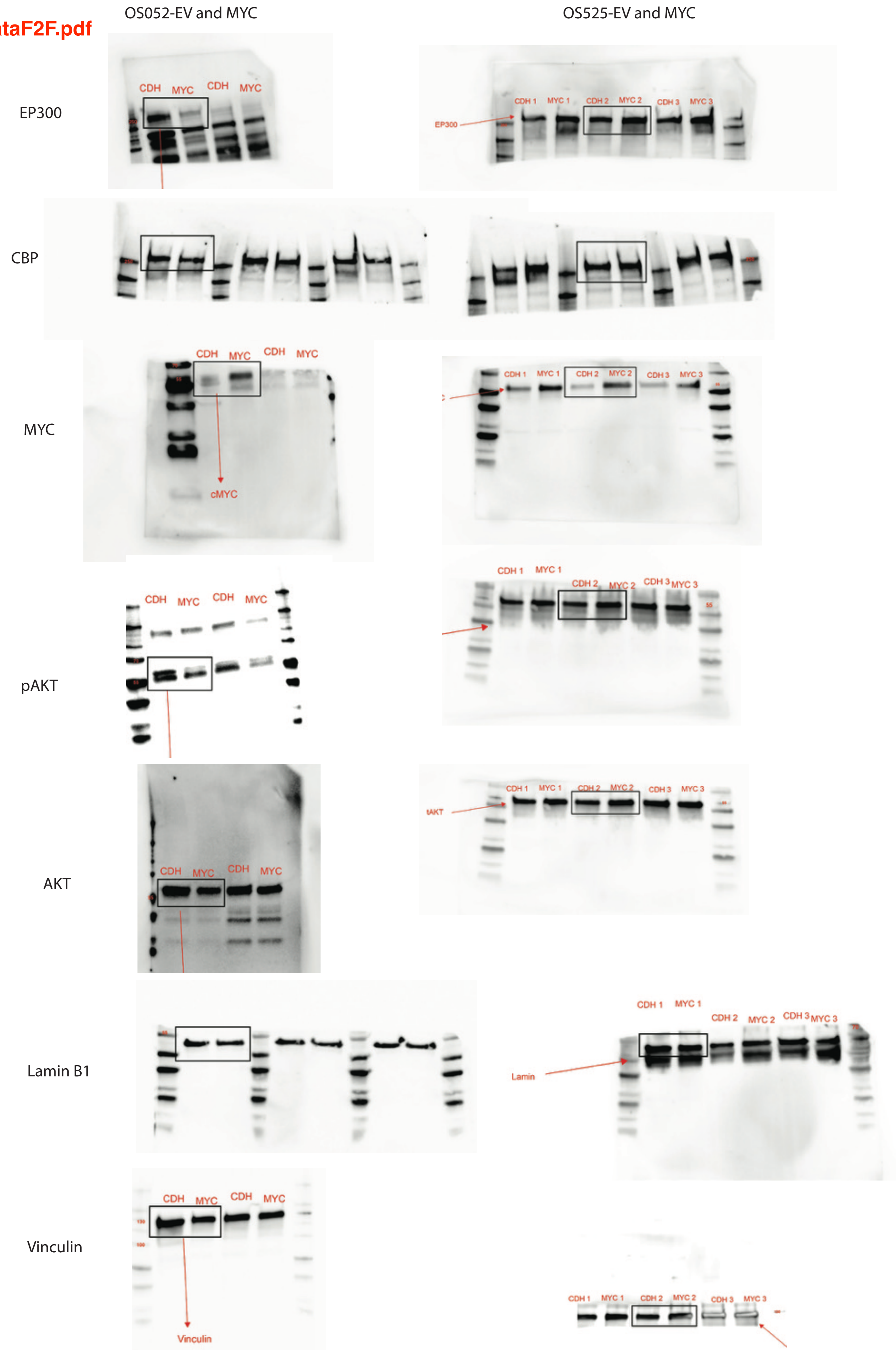

SourceDataF2i.pdf

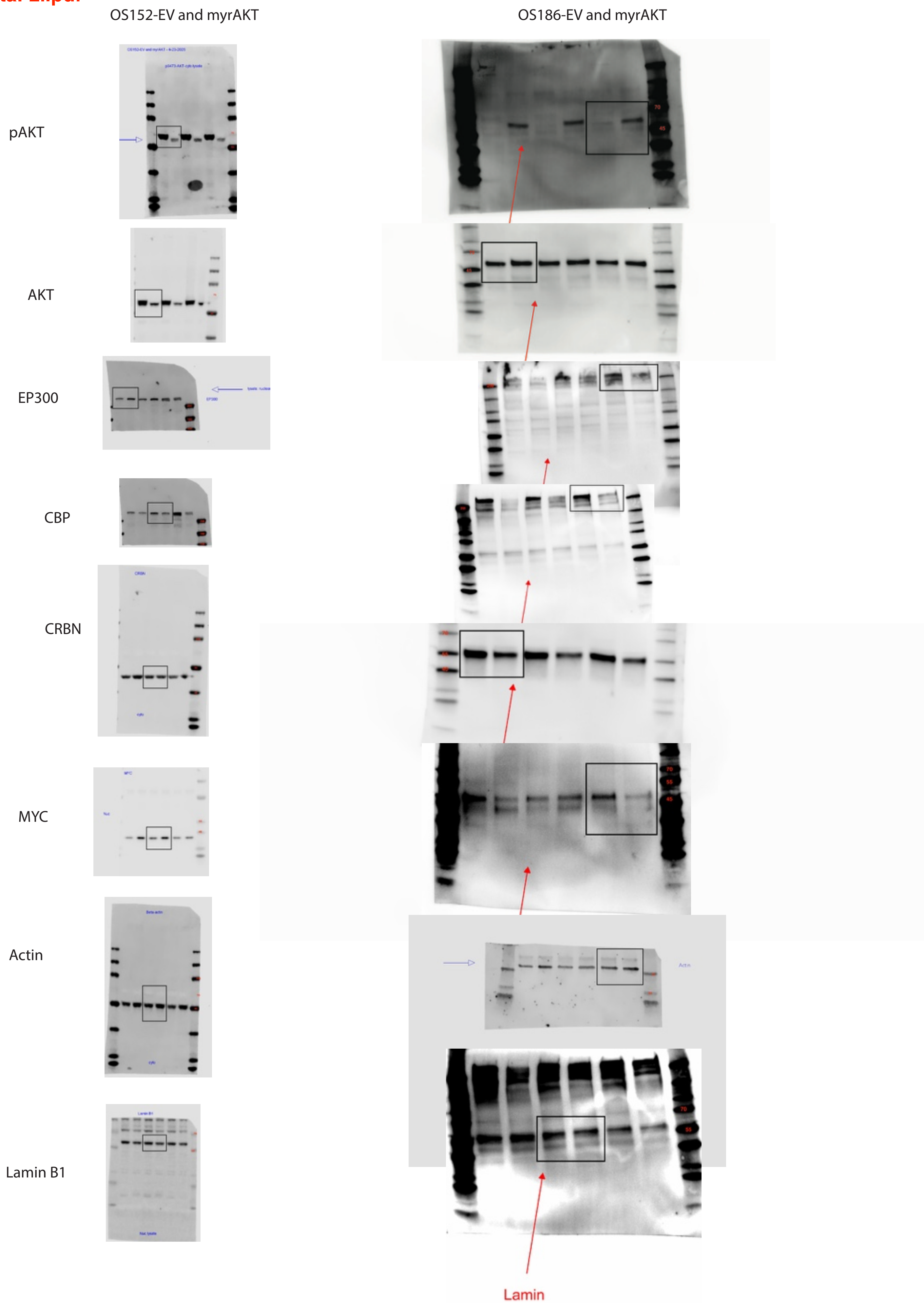

SourceDataF2K.pdf

OS152-EV and myr-AKT

+/- JQAD1

CBP

EP300

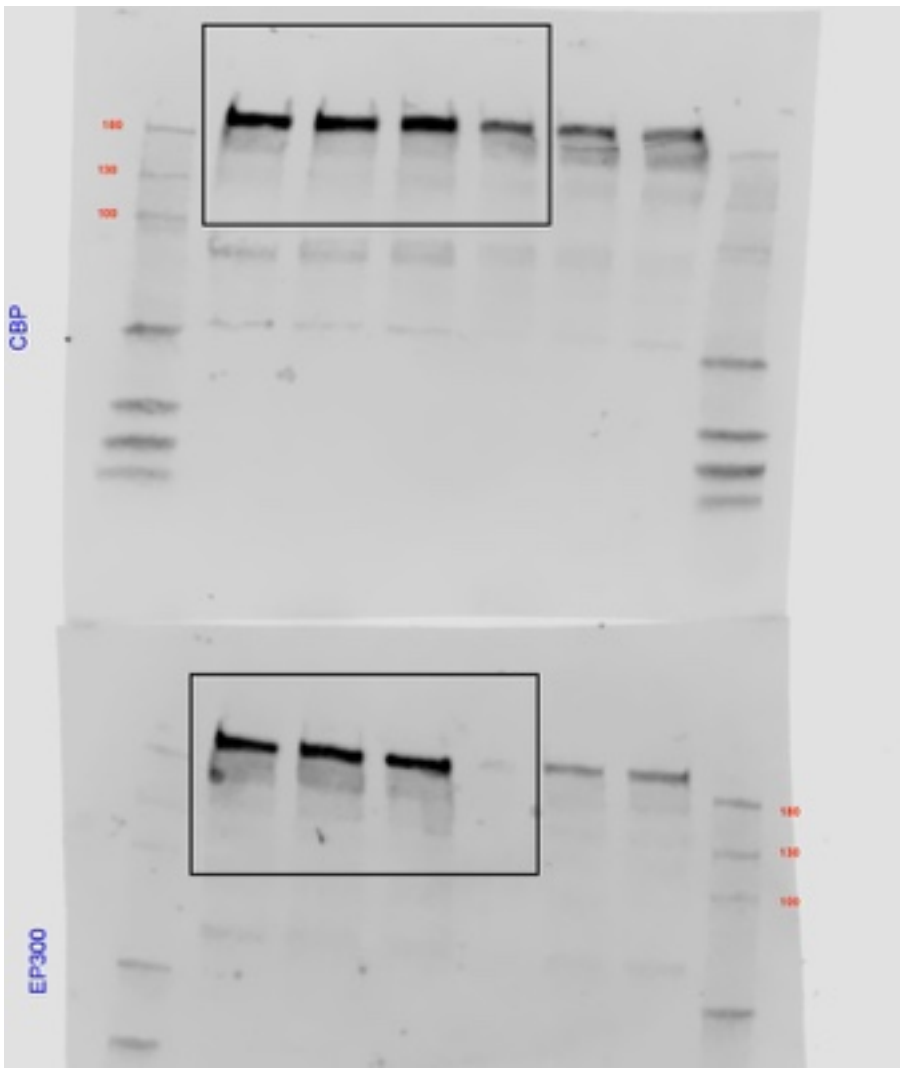

H3

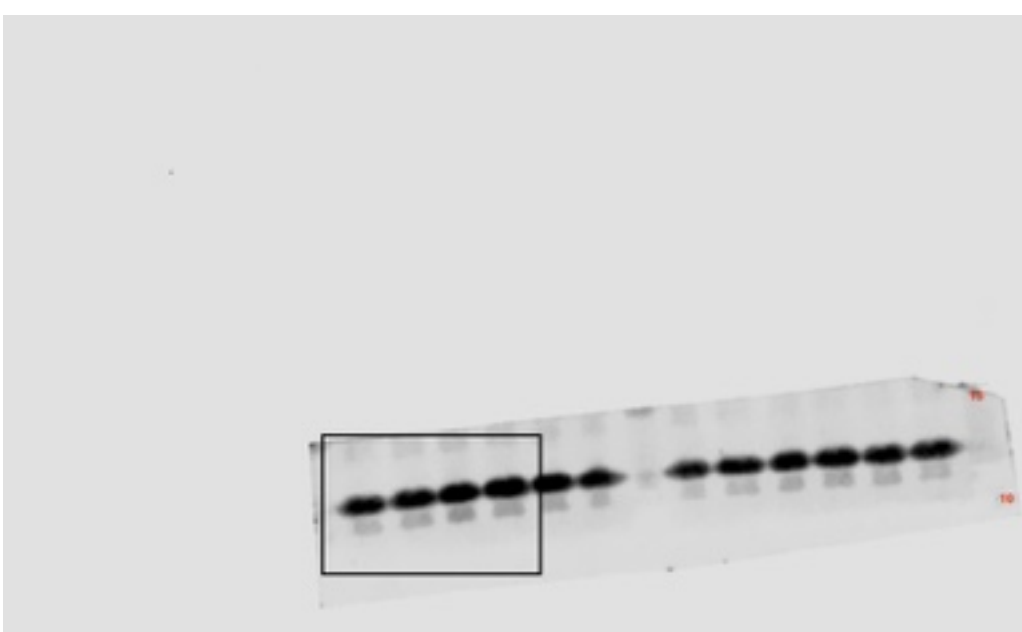

OS186-EV and myr-AKT

+/- JQAD1

EP300

CBP

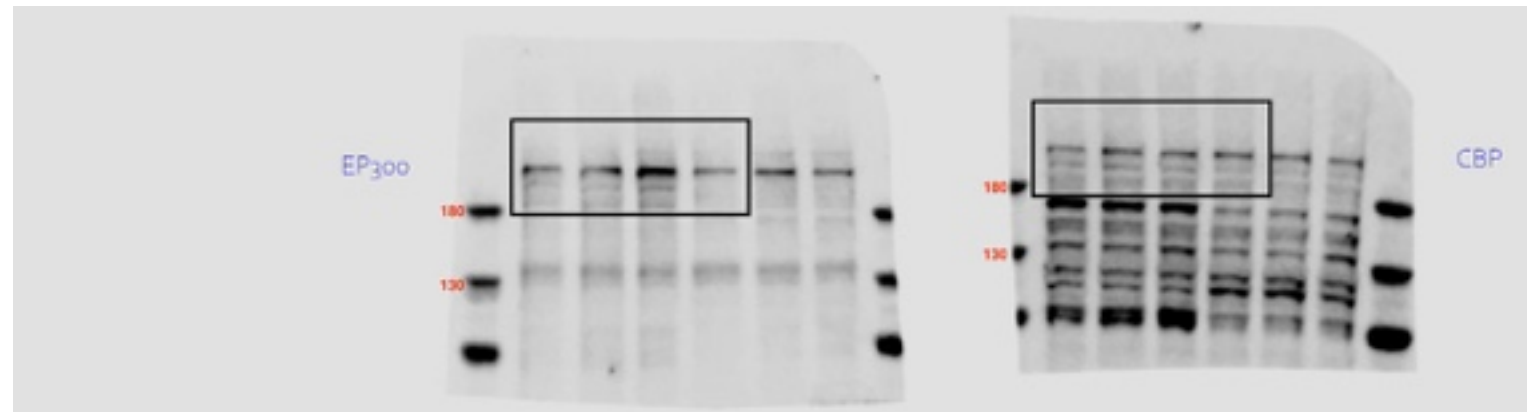

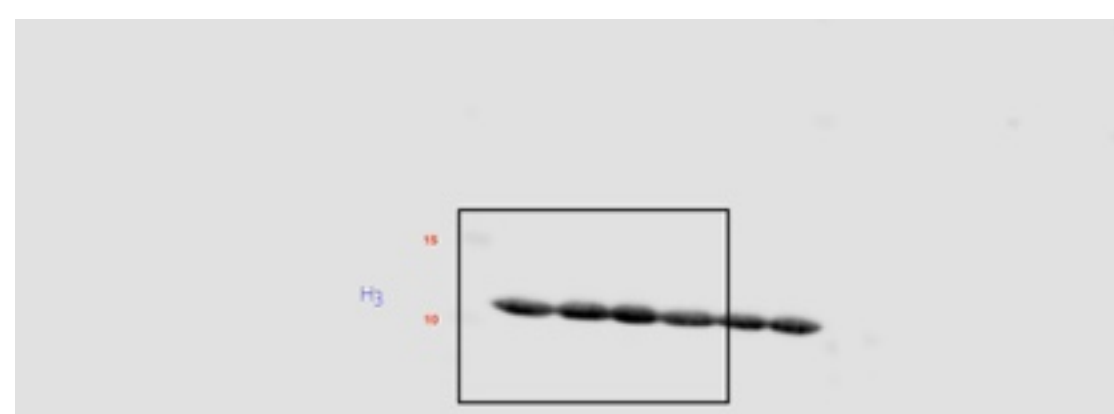

Supplement: Supplementary file 1 [file jem_20251898_sourcedataf2.pdf]
