# Supplementary material for: Dysregulated AKT signaling reprograms osteosarcoma to drive selective reliance on EP300
Source: J Exp Med. 2026 Oct 20;223(11):e20251898. doi: 10.1084/jem.20251898 (PMC13682445; doi:10.1084/jem.20251898)
Supplement: Supplementary file 2 [file jem_20251898_sourcedataf4.pdf]

SourceDataF4H.pdf

puromycin

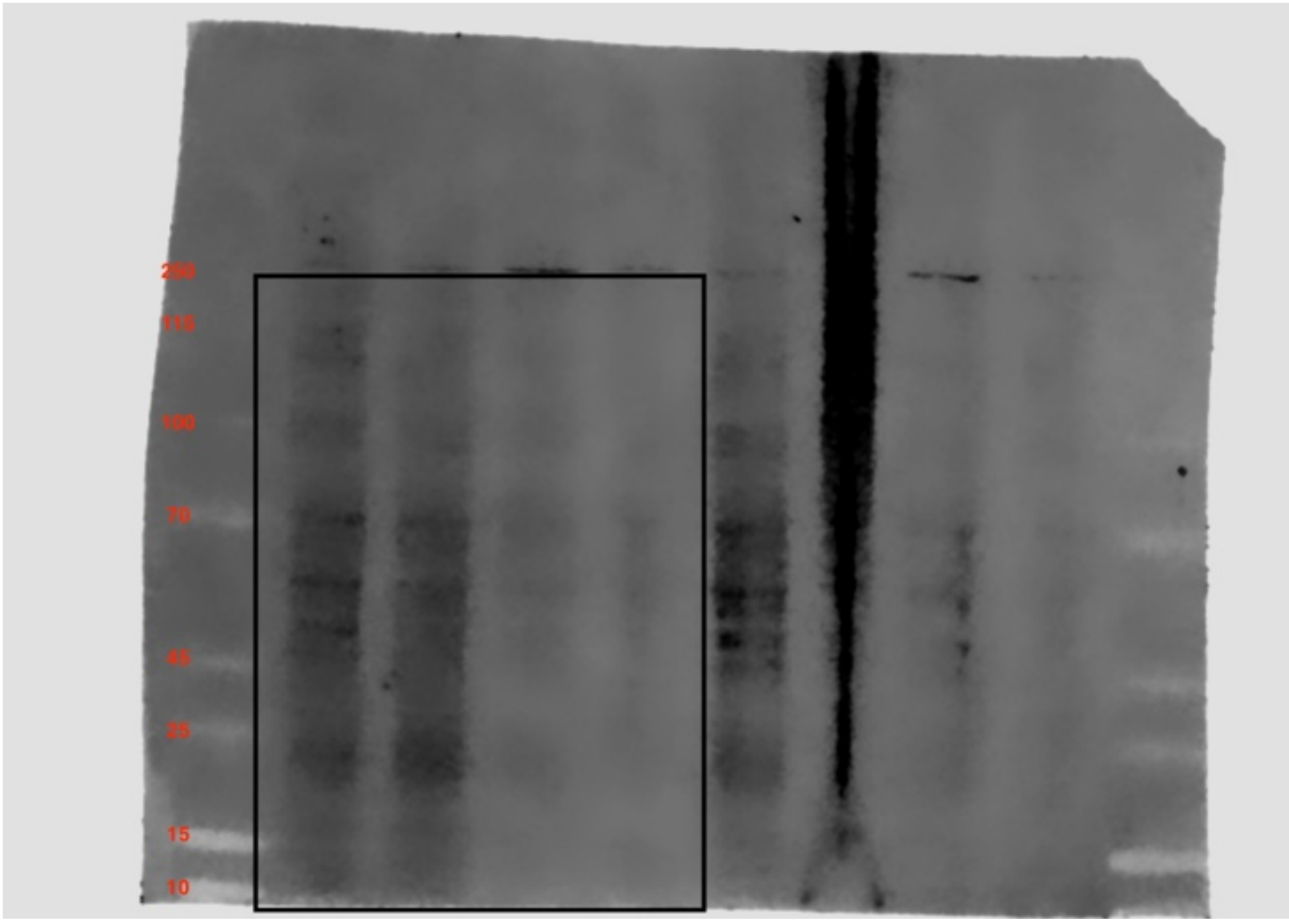

vinculin

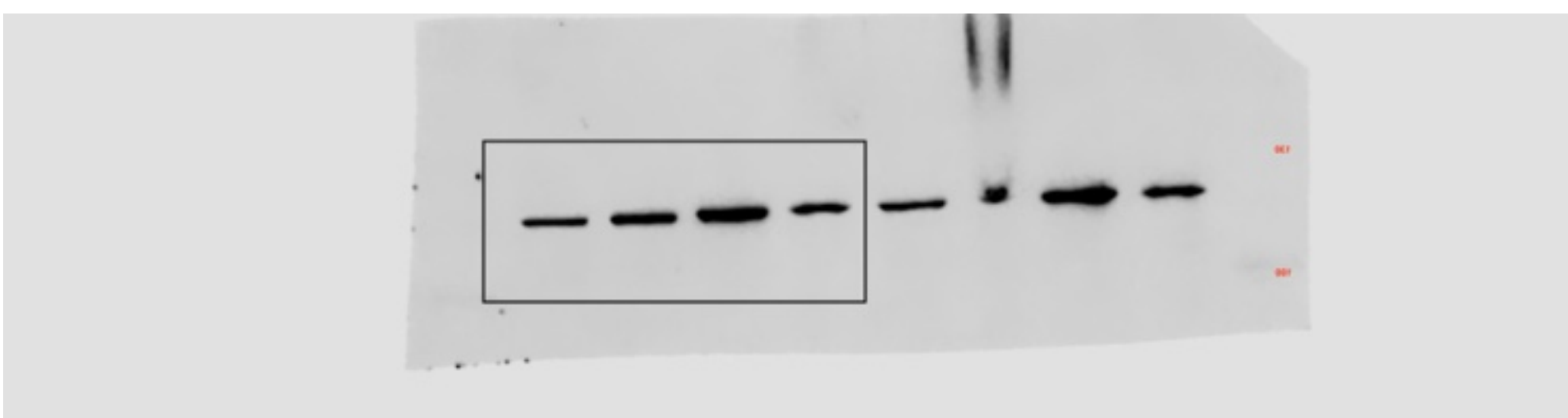

SourceDataF4i.pdf

EP300

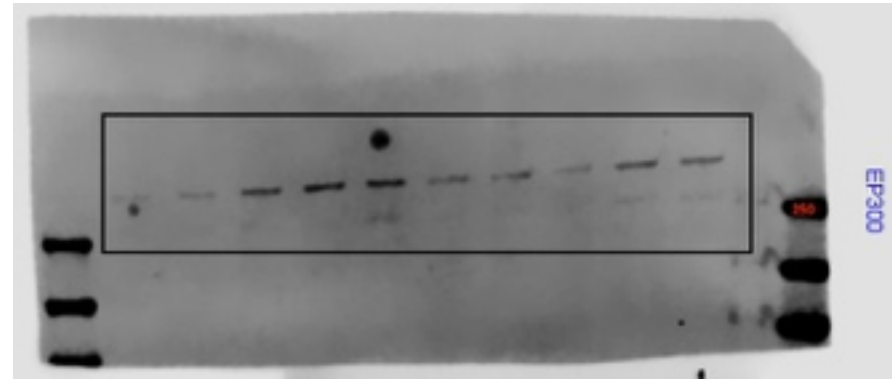

CBP

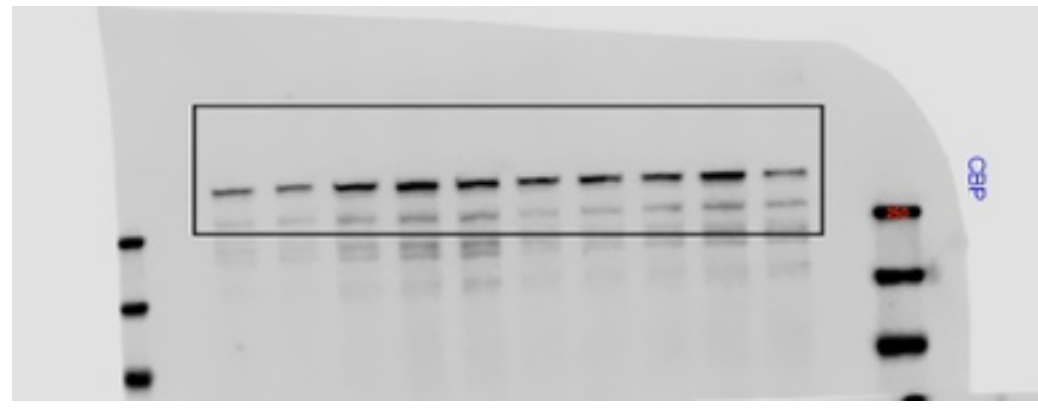

pAKT

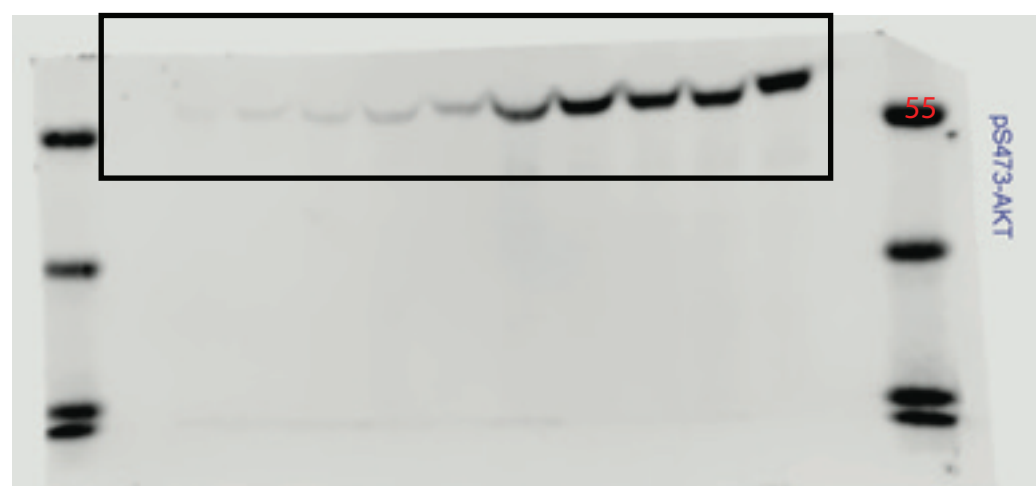

Total AKT

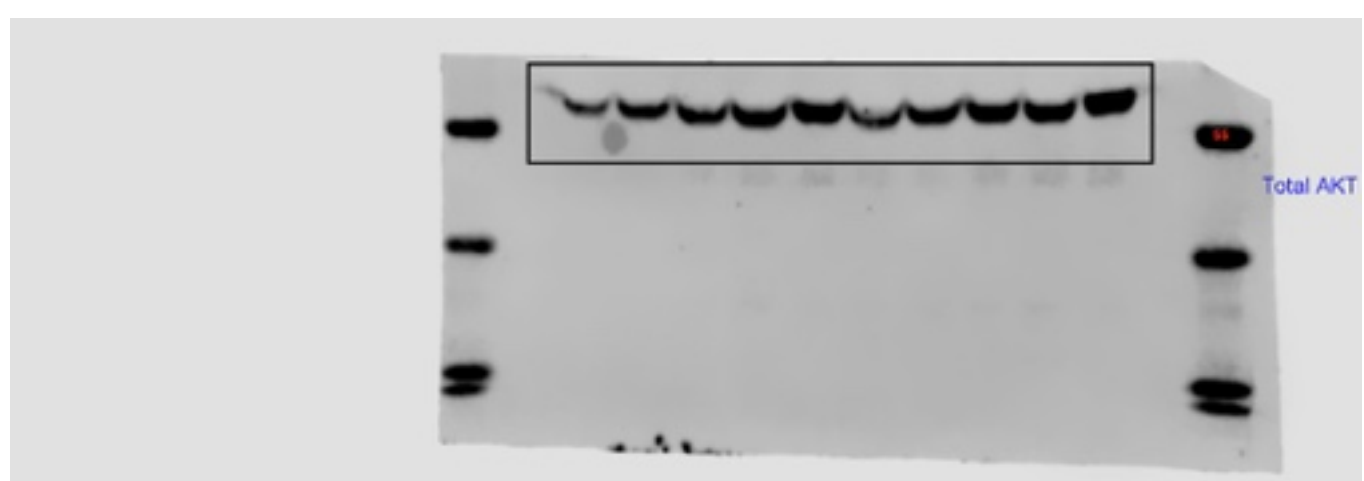

CRBN

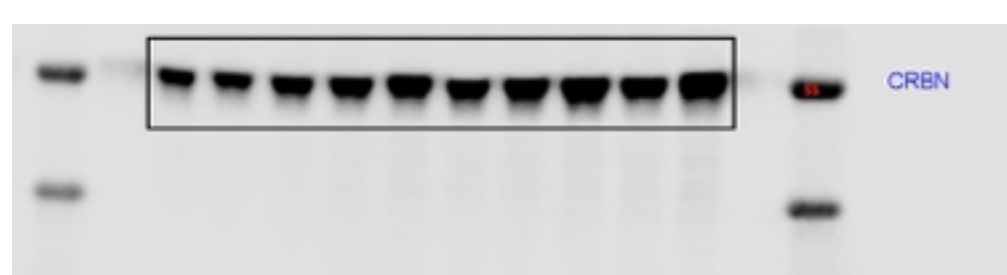

MYC

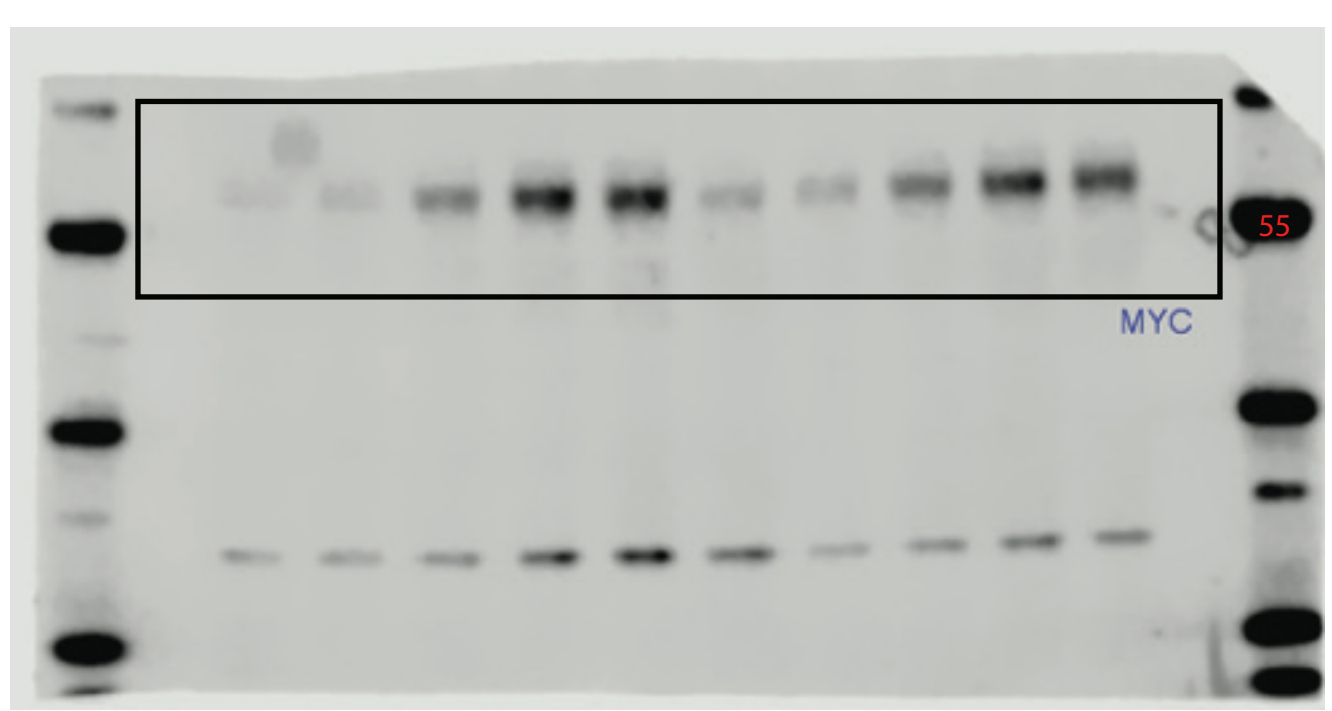

Lamin B1

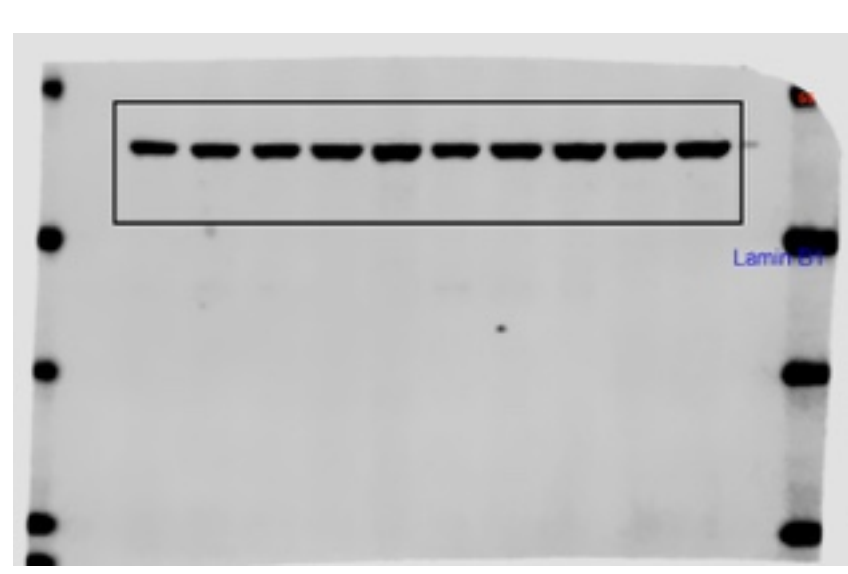

SourceDataF4k.pdf

pAKT

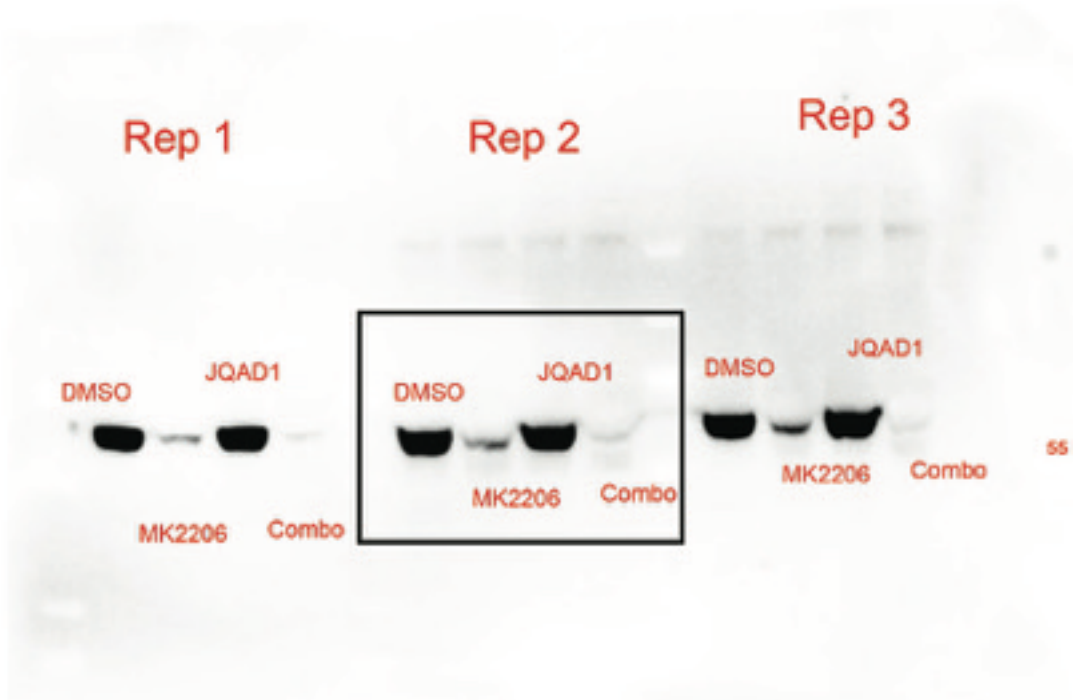

AKT

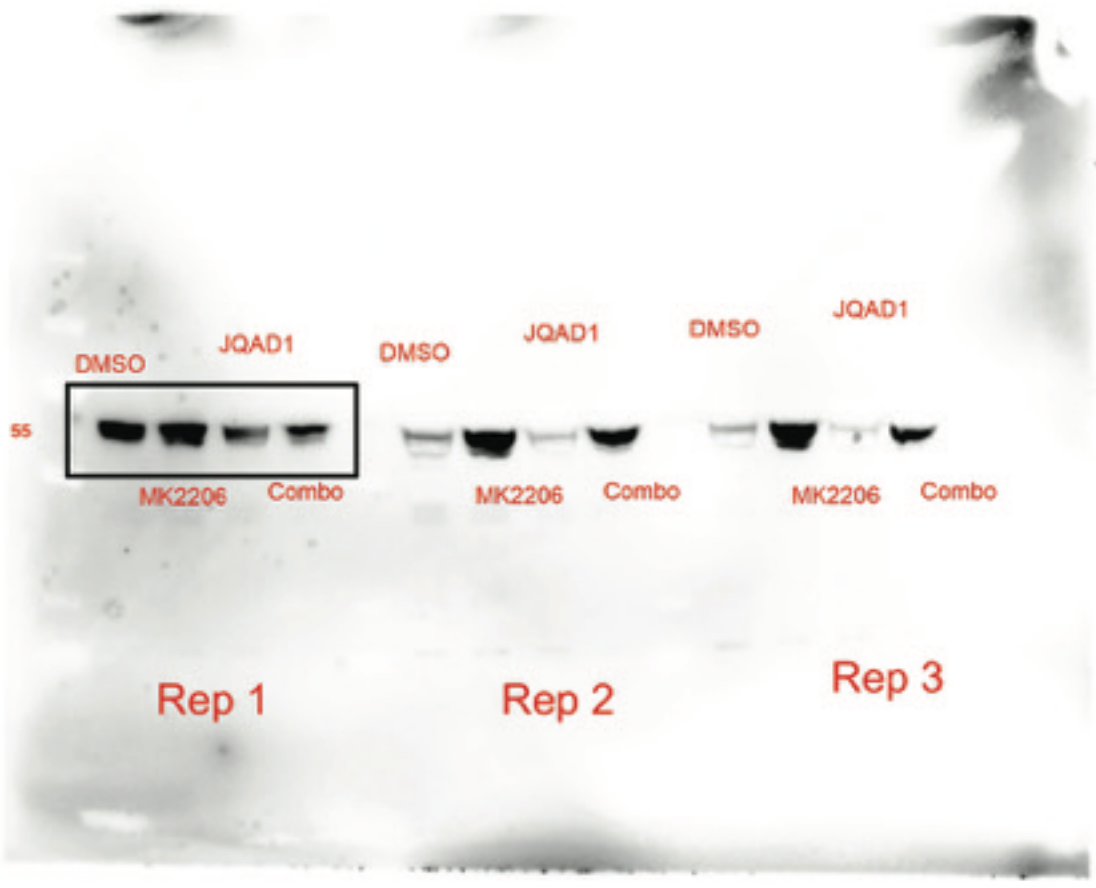

pp70S6K1

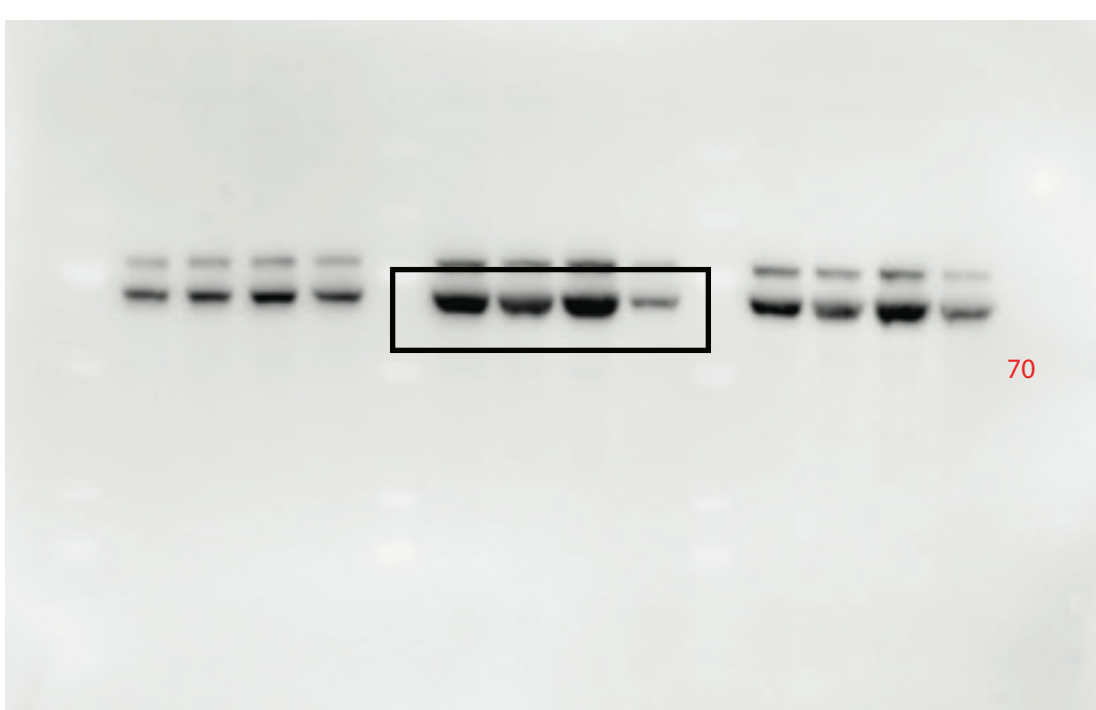

p70S6K1

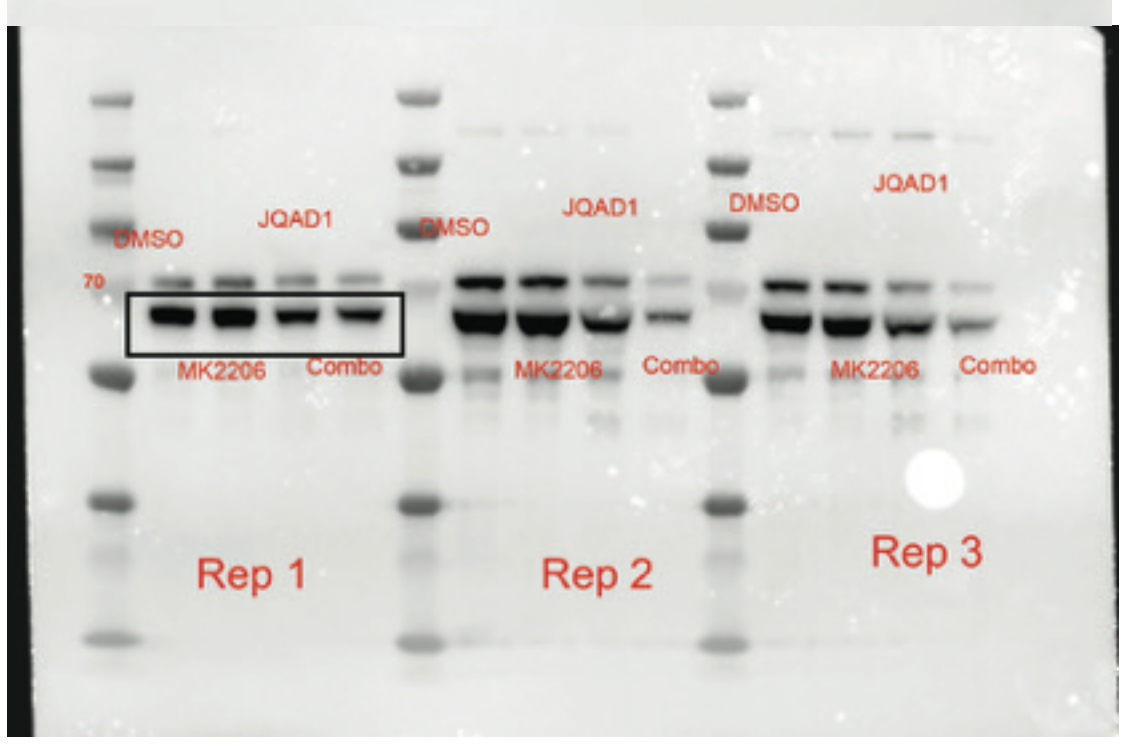

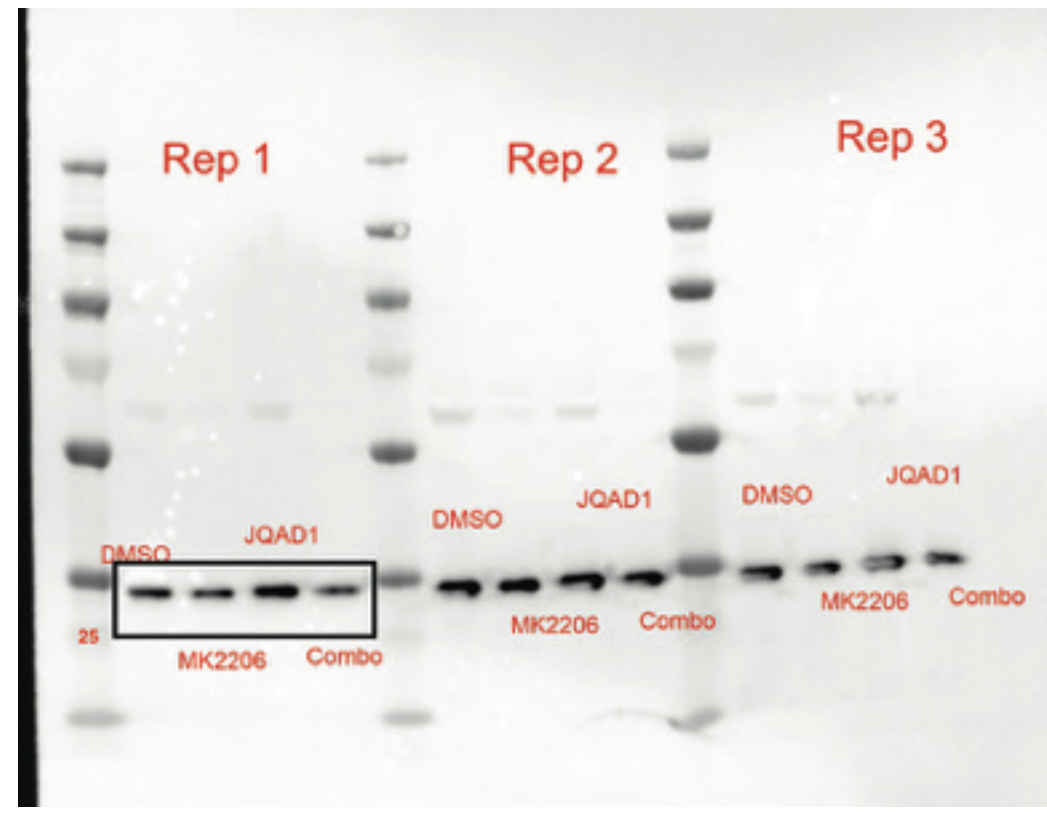

pS6

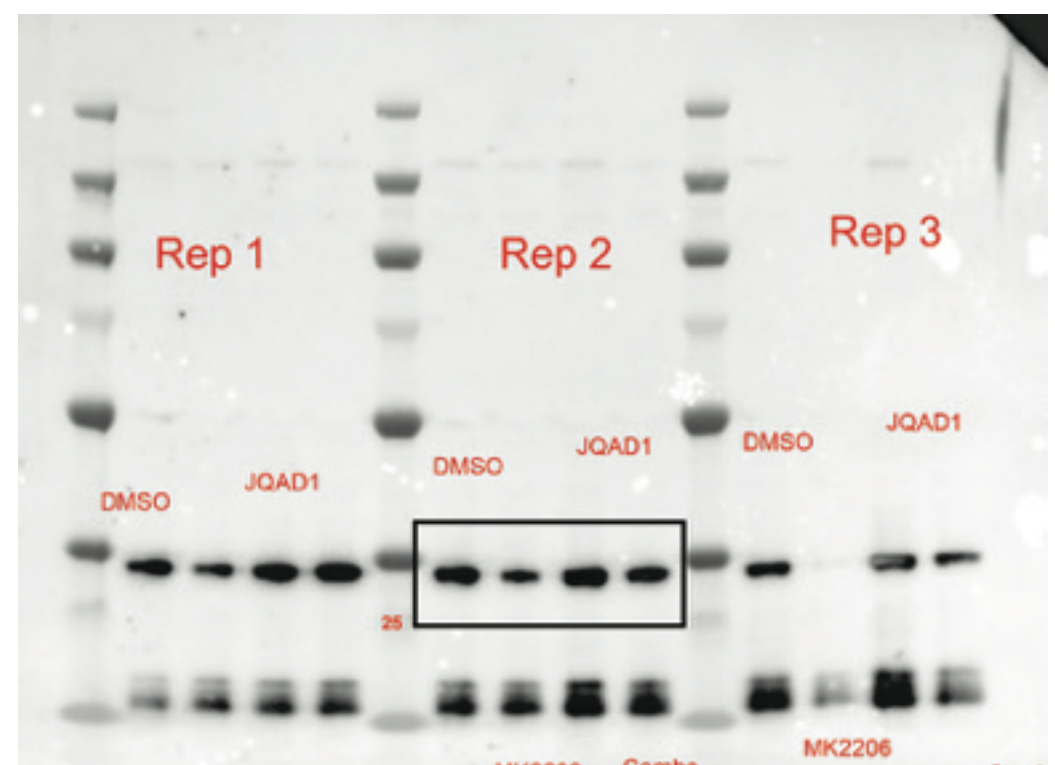

S6

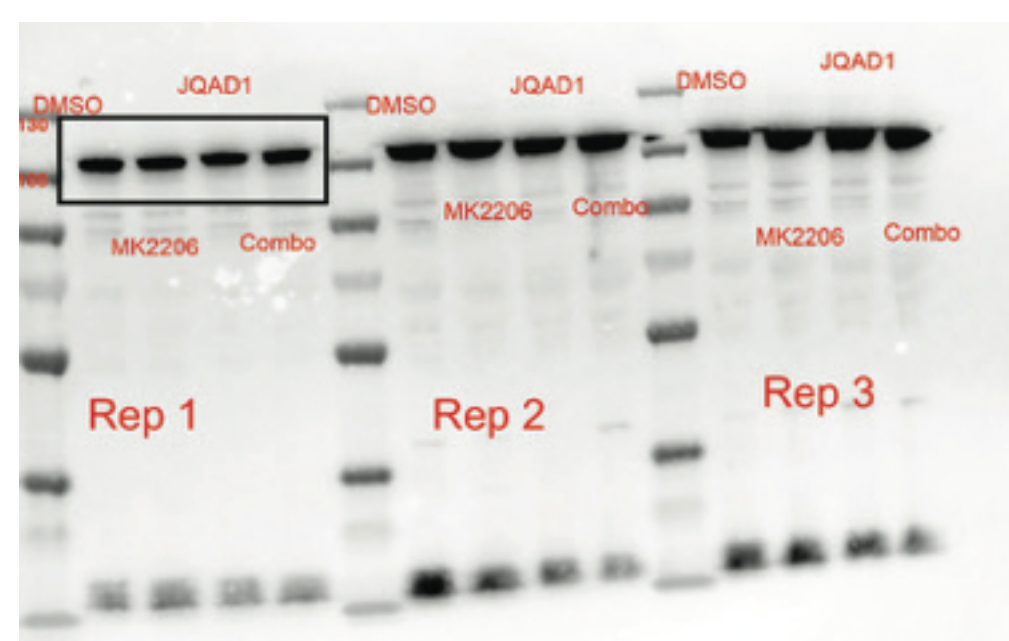

vinculin
