# Supplementary material for: Dysregulated AKT signaling reprograms osteosarcoma to drive selective reliance on EP300
Source: J Exp Med. 2026 Oct 20;223(11):e20251898. doi: 10.1084/jem.20251898 (PMC13682445; doi:10.1084/jem.20251898)

SourceDataFS1F.pdf

pAKT

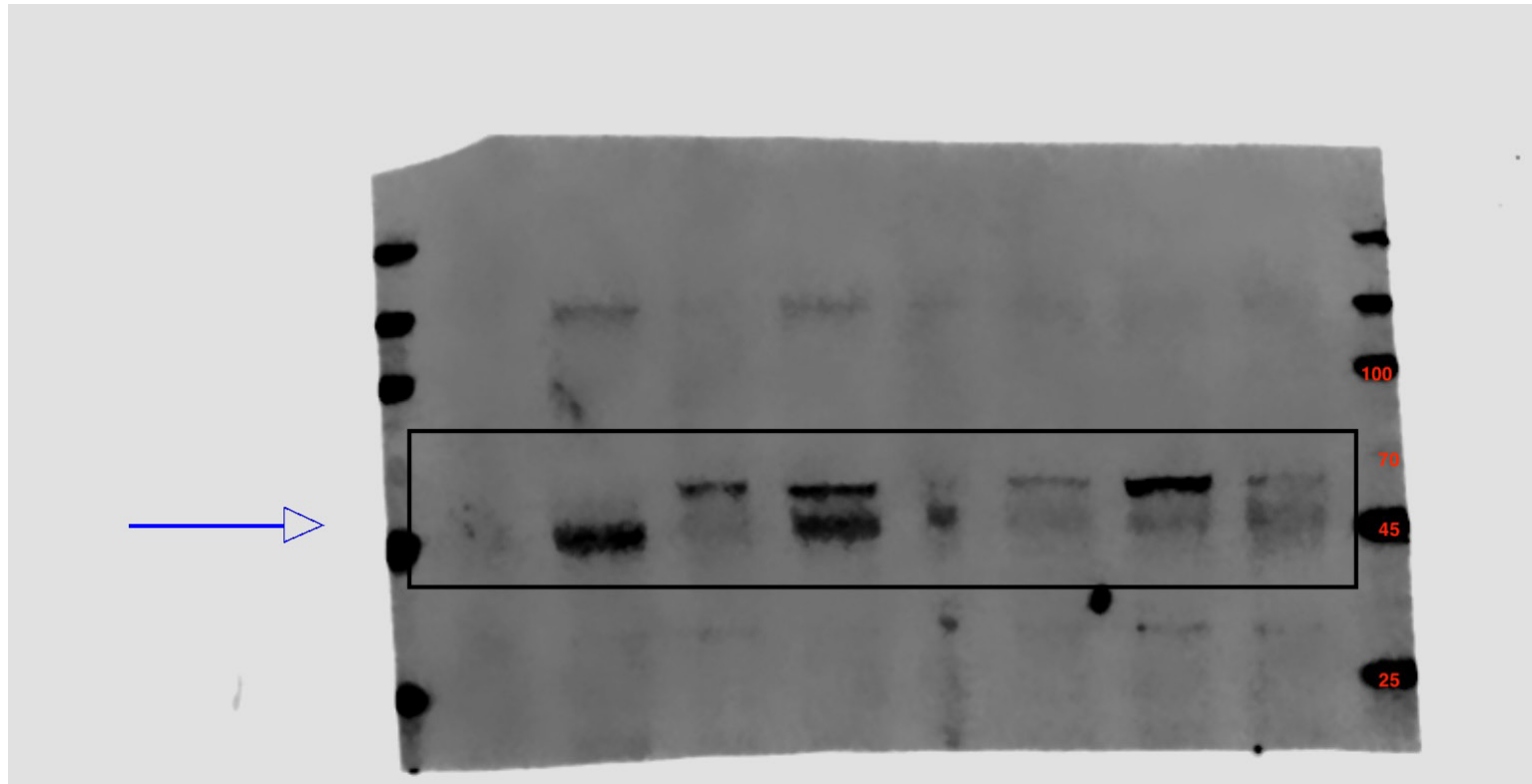

AKT

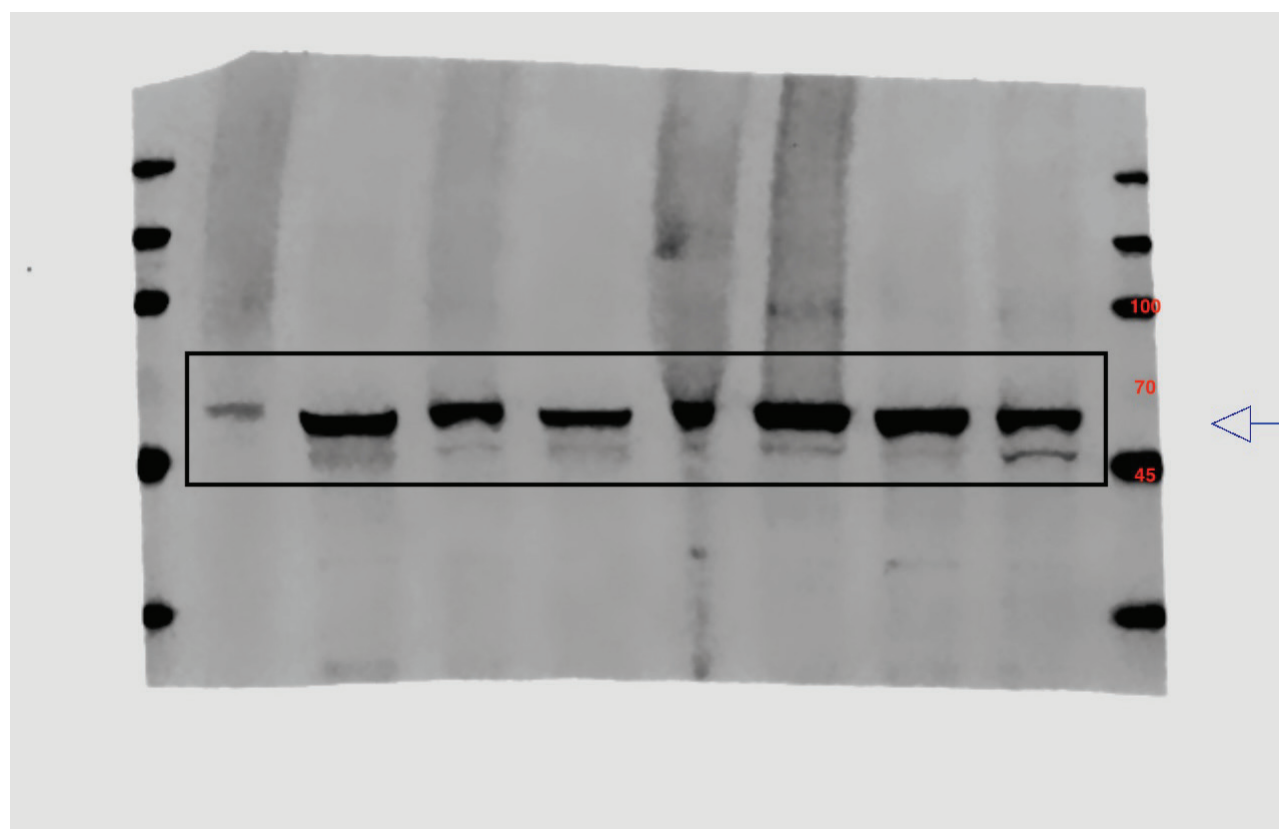

PTEN

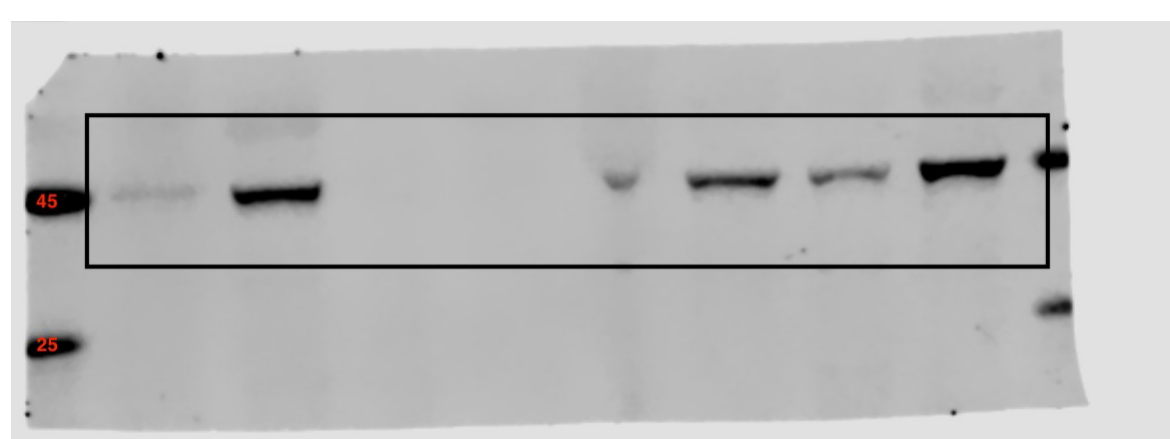

Vinculin

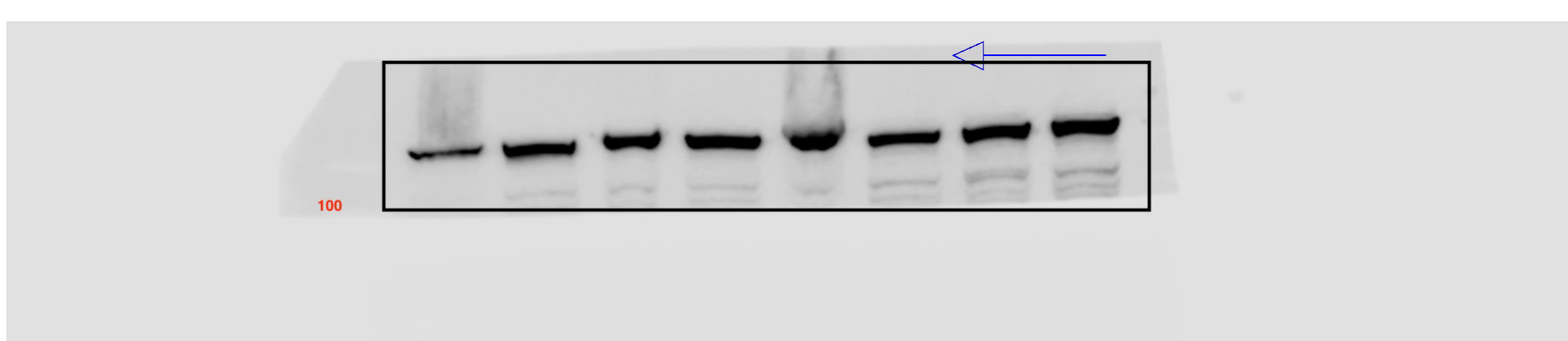

Supplement: Supplementary file 3 [file jem_20251898_sourcedatafs1.pdf]
