# Supplementary material for: Dysregulated AKT signaling reprograms osteosarcoma to drive selective reliance on EP300
Source: J Exp Med. 2026 Oct 20;223(11):e20251898. doi: 10.1084/jem.20251898 (PMC13682445; doi:10.1084/jem.20251898)
Supplement: Supplementary file 4 [file jem_20251898_sourcedatafs2.pdf]

SourceDataFS2C.pdf

EP300

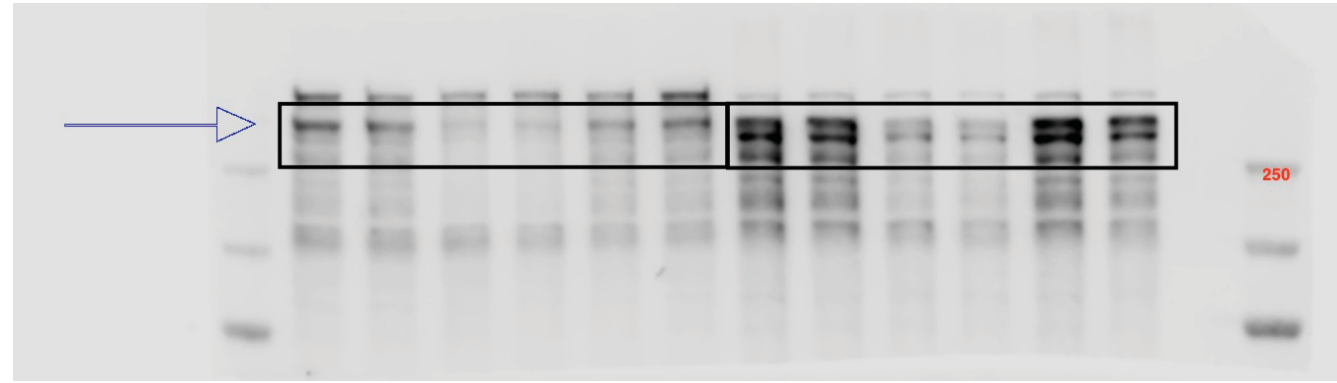

CBP

OS052 and OS525

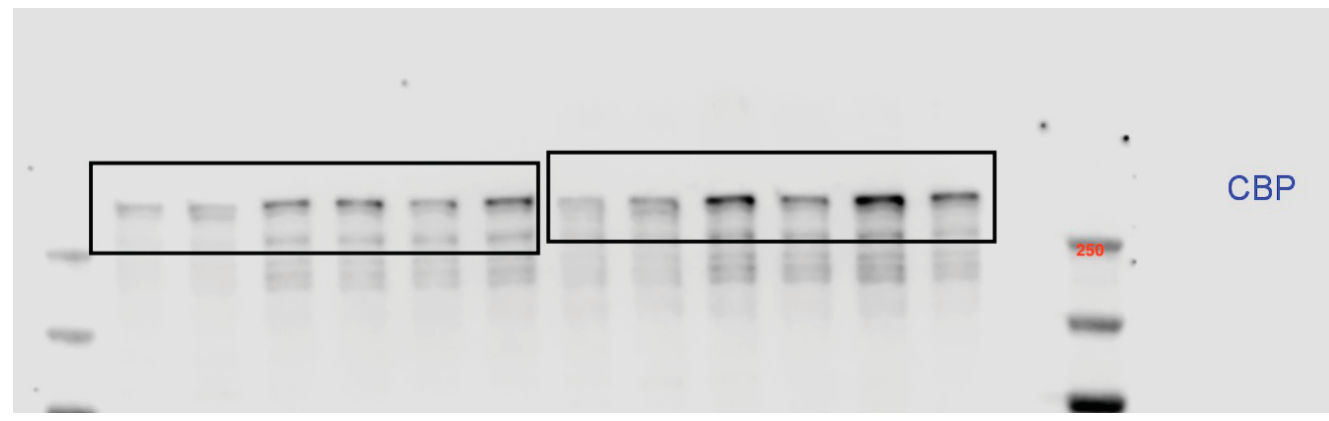

Lamin B1

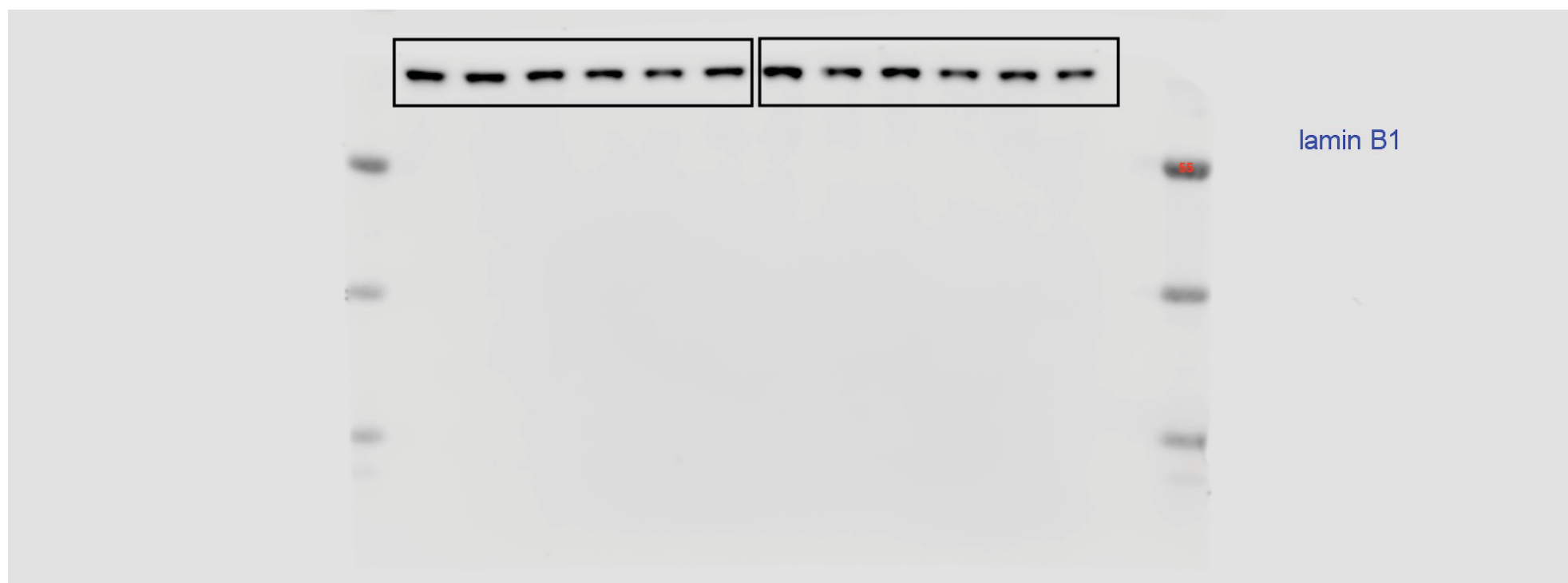

EP300

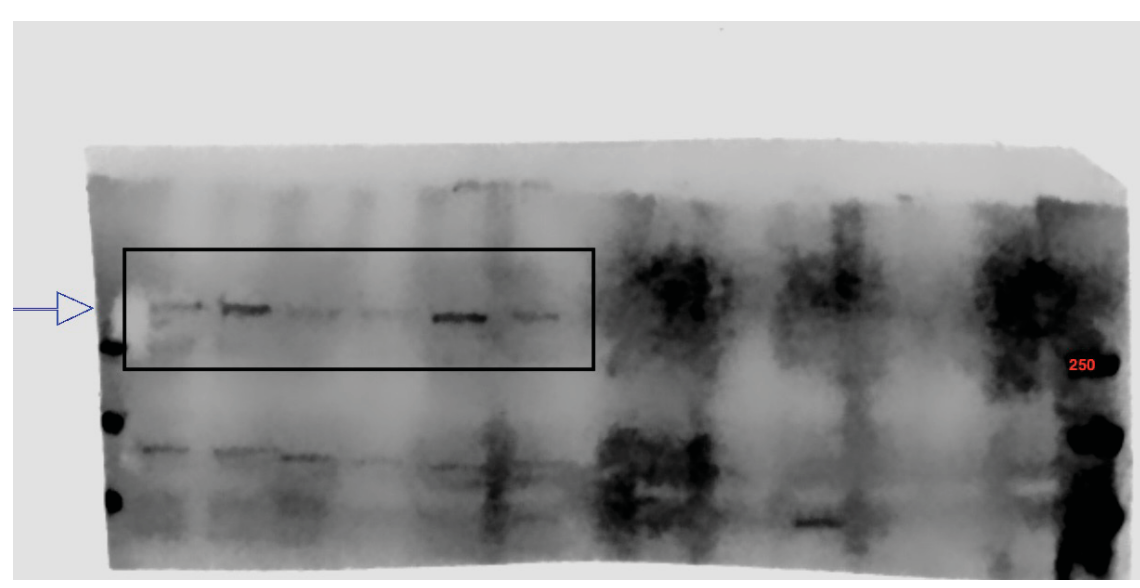

CBP

OS186

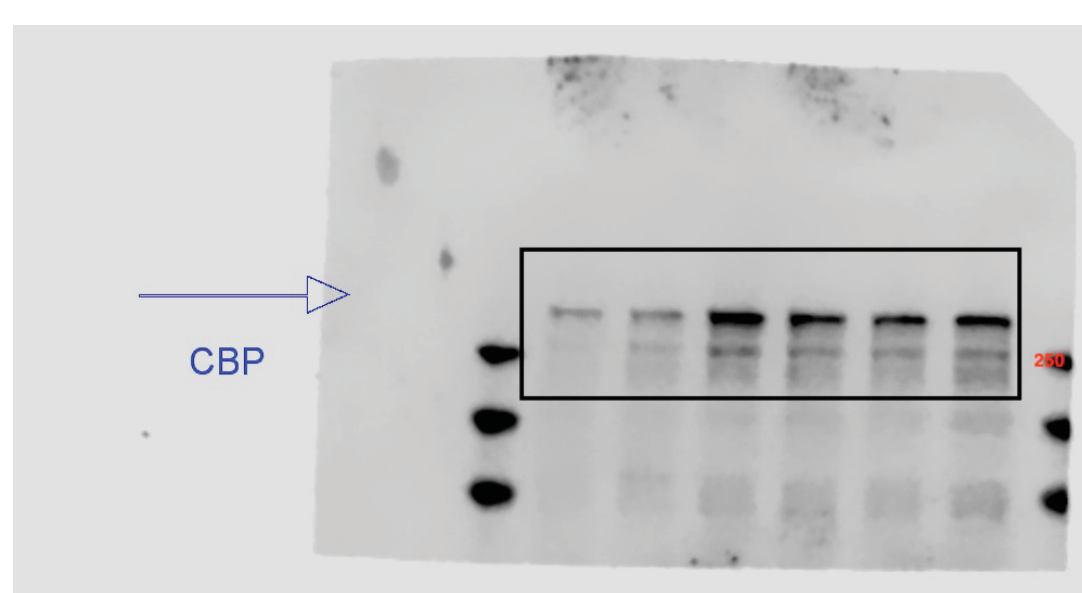

H3

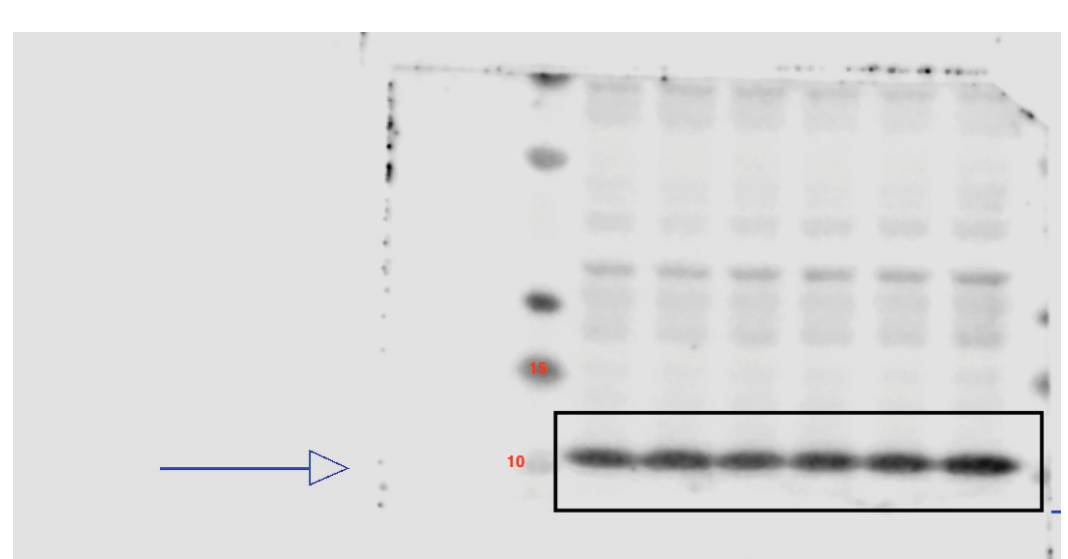

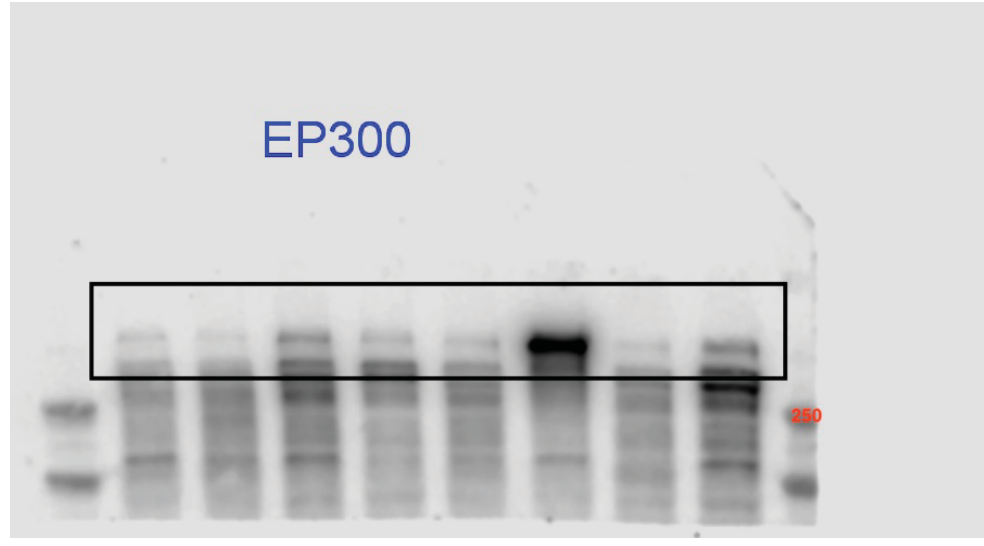
EP300

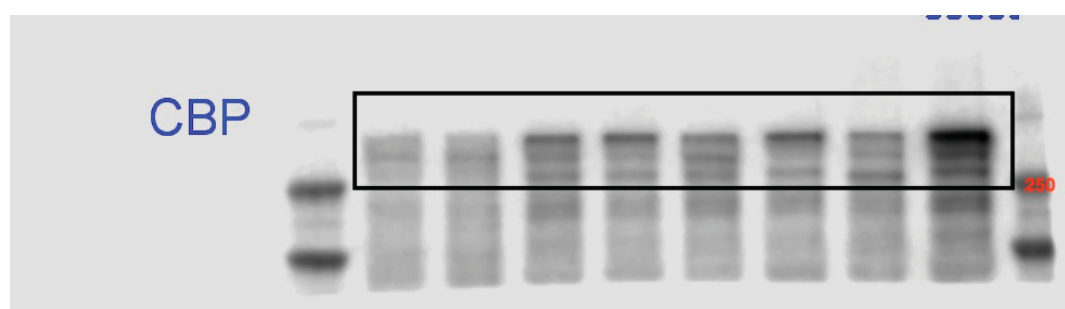
CBP

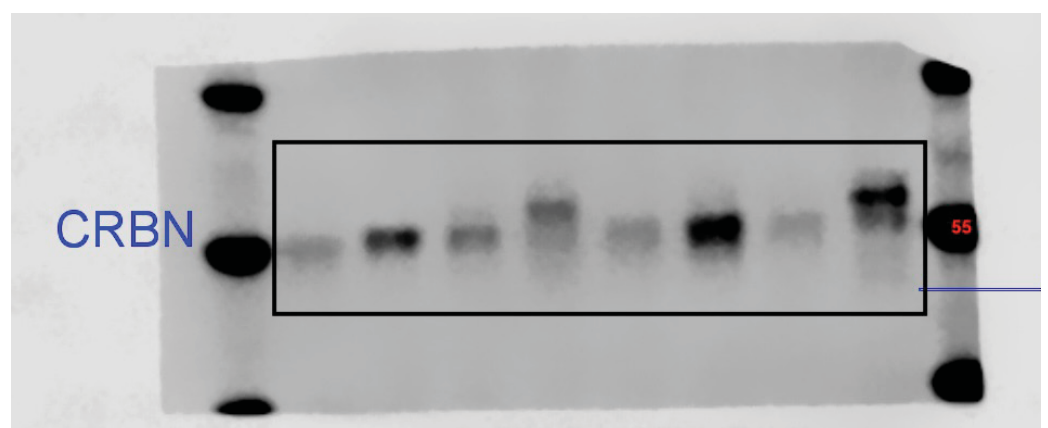
CRBN
55

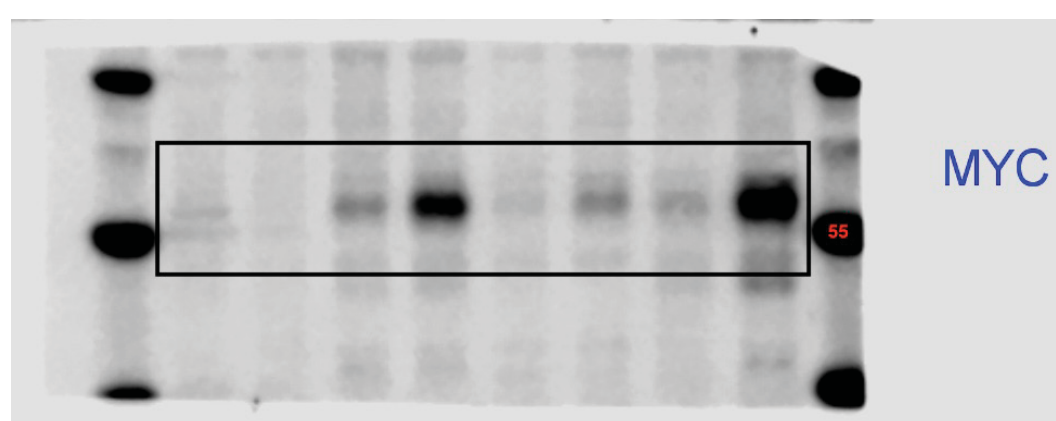
MYC
55

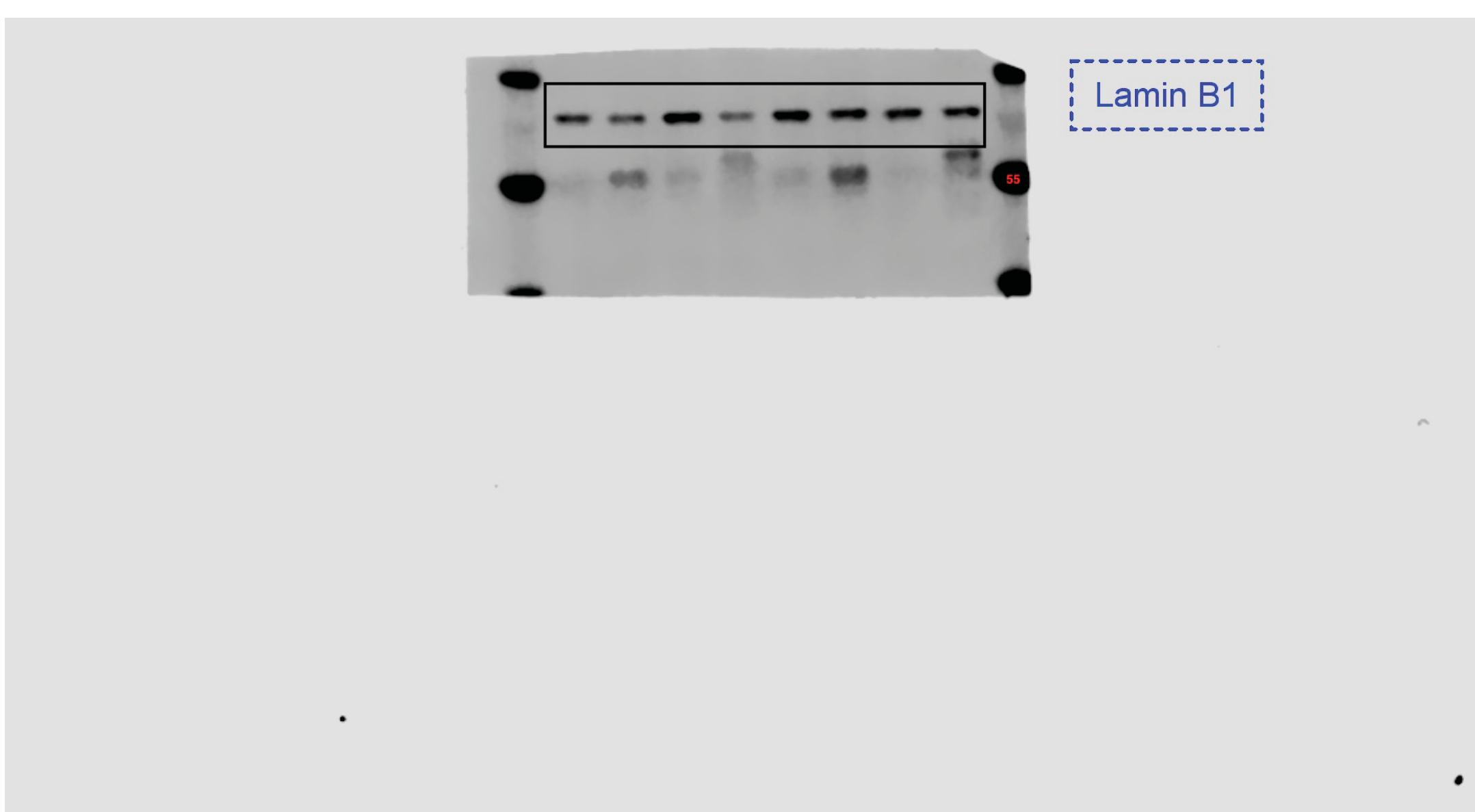
Lamin B1
55
