# Supplementary material for: Dysregulated AKT signaling reprograms osteosarcoma to drive selective reliance on EP300
Source: J Exp Med. 2026 Oct 20;223(11):e20251898. doi: 10.1084/jem.20251898 (PMC13682445; doi:10.1084/jem.20251898)

Fig S4B Source Data

Both lines

pAKT

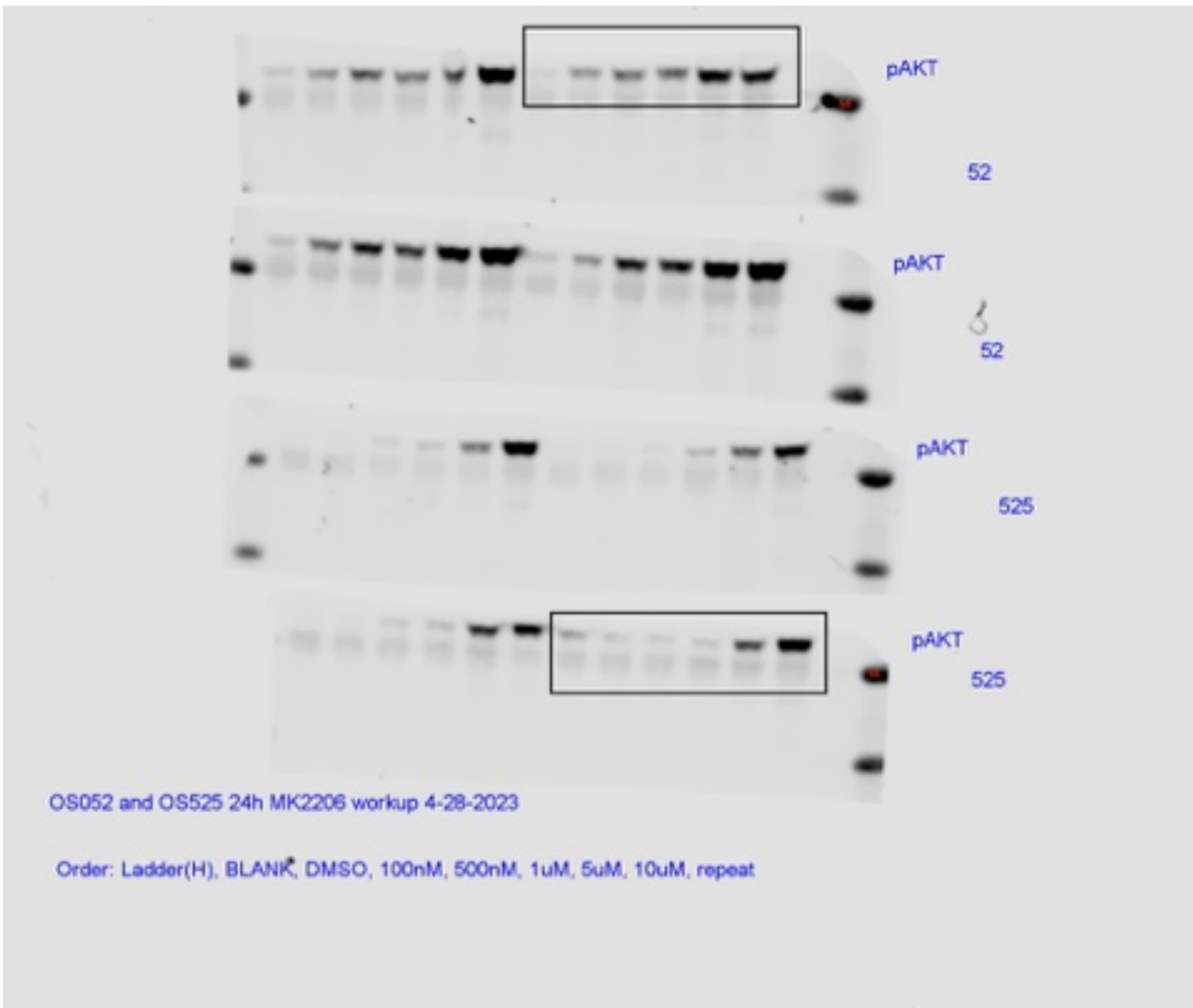

CBP

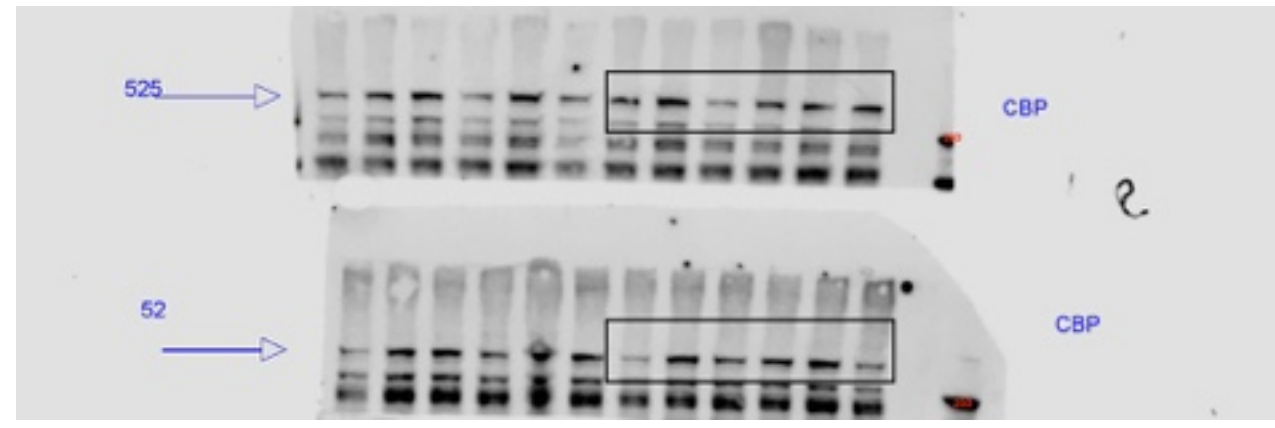

Vinculin

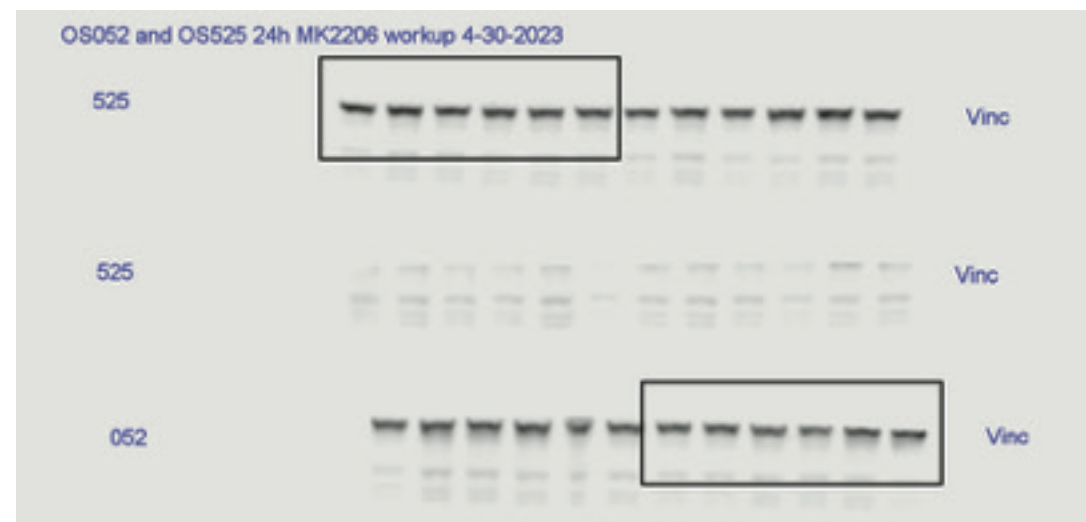

OS052

OS525

CRBN

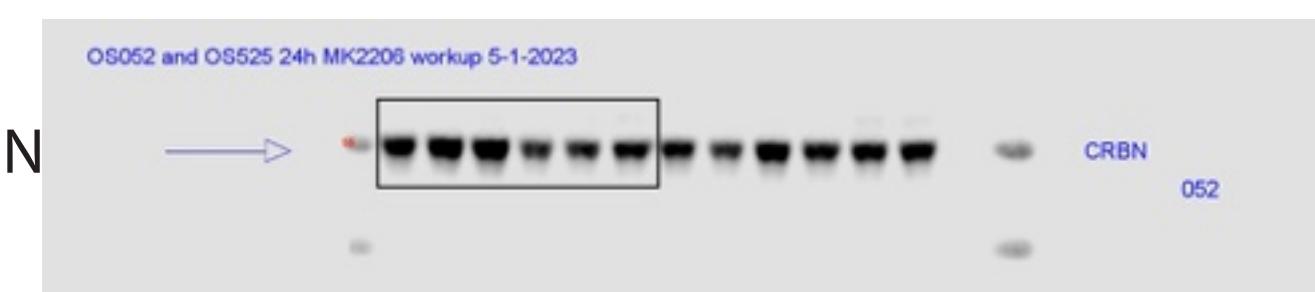

CRBN

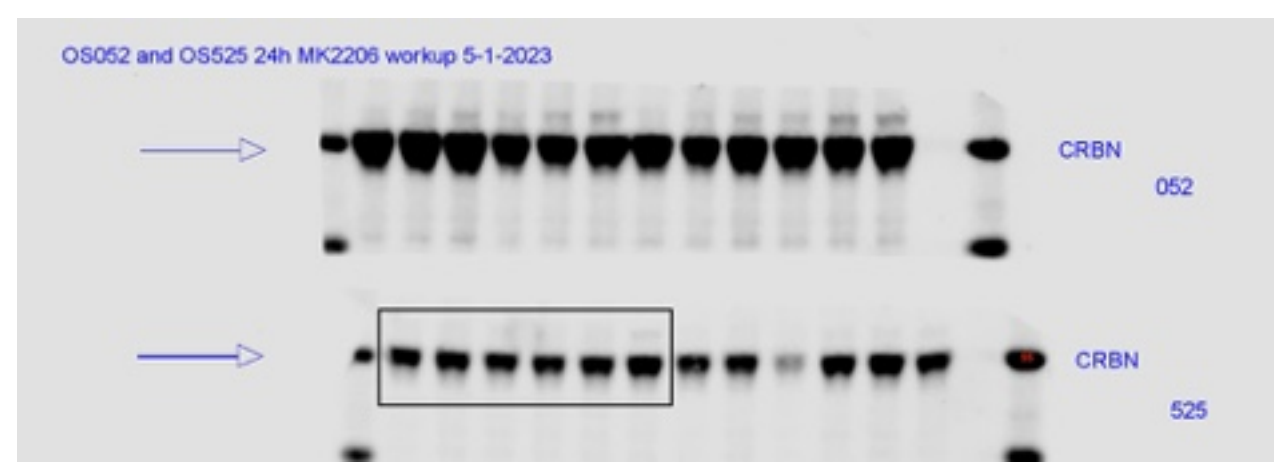

EP300

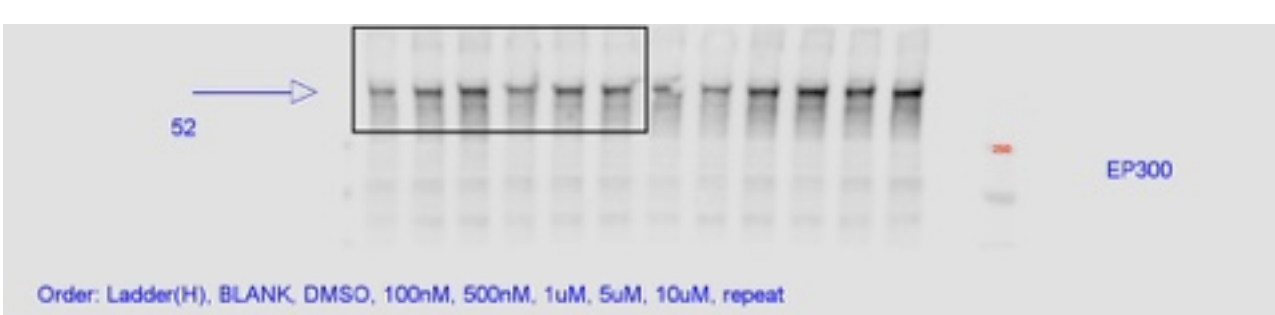

EP300

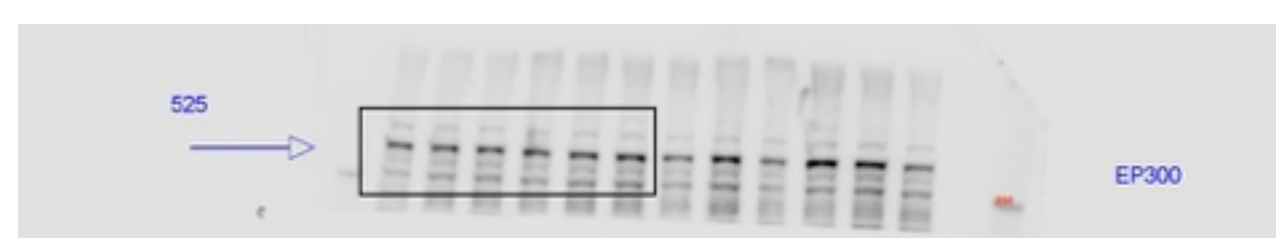

AKT

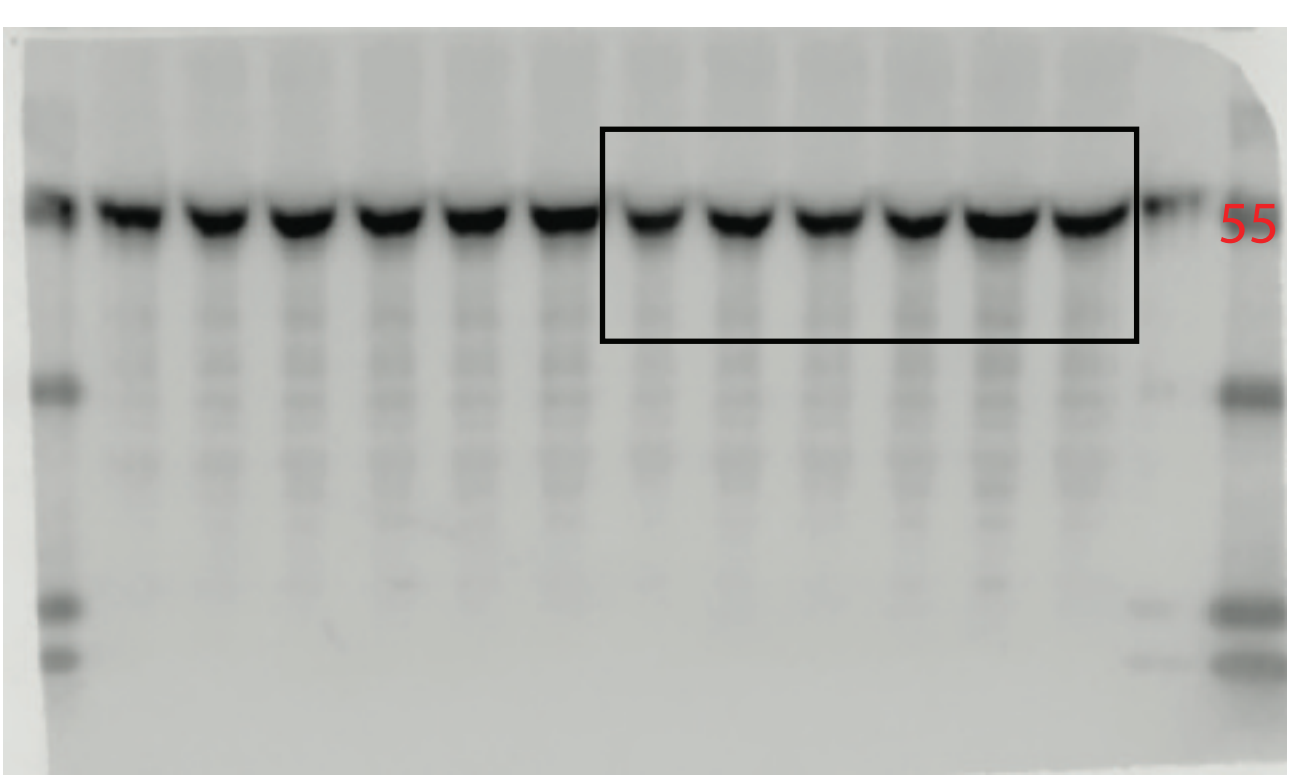

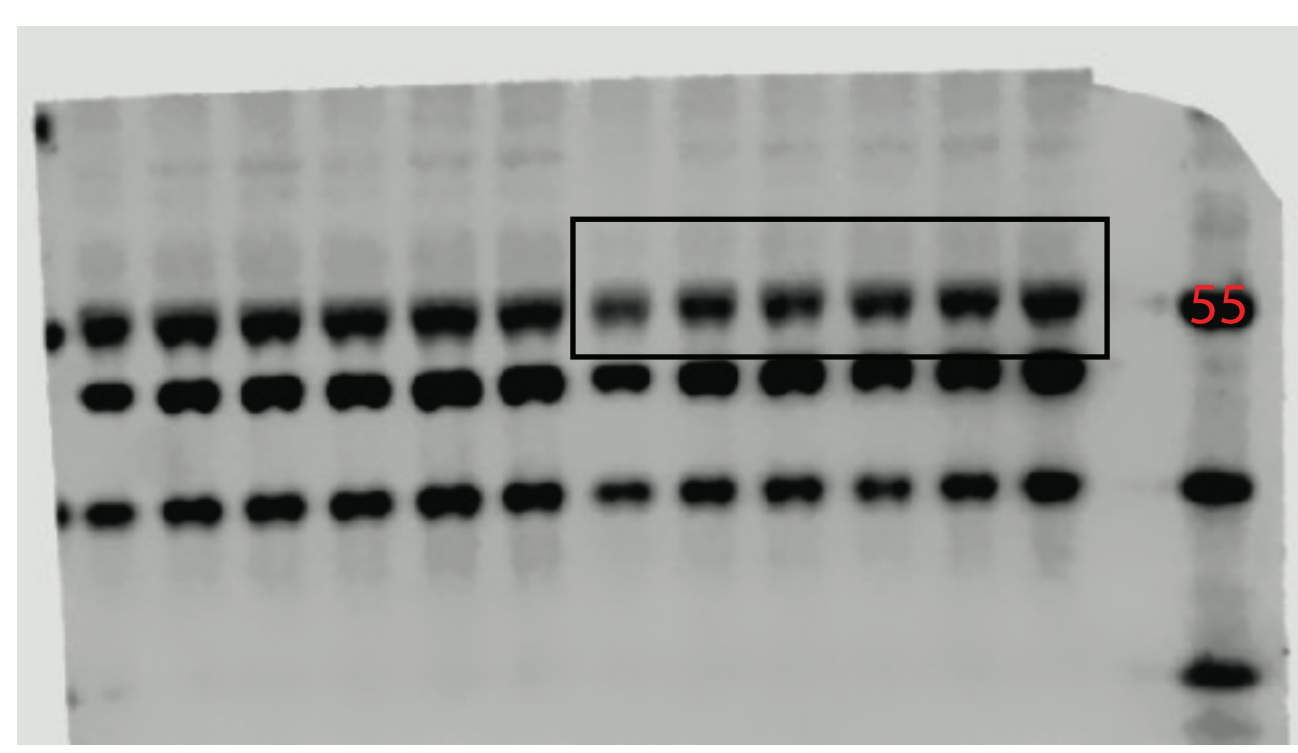

Fig S4N Source Data

p4EBP1

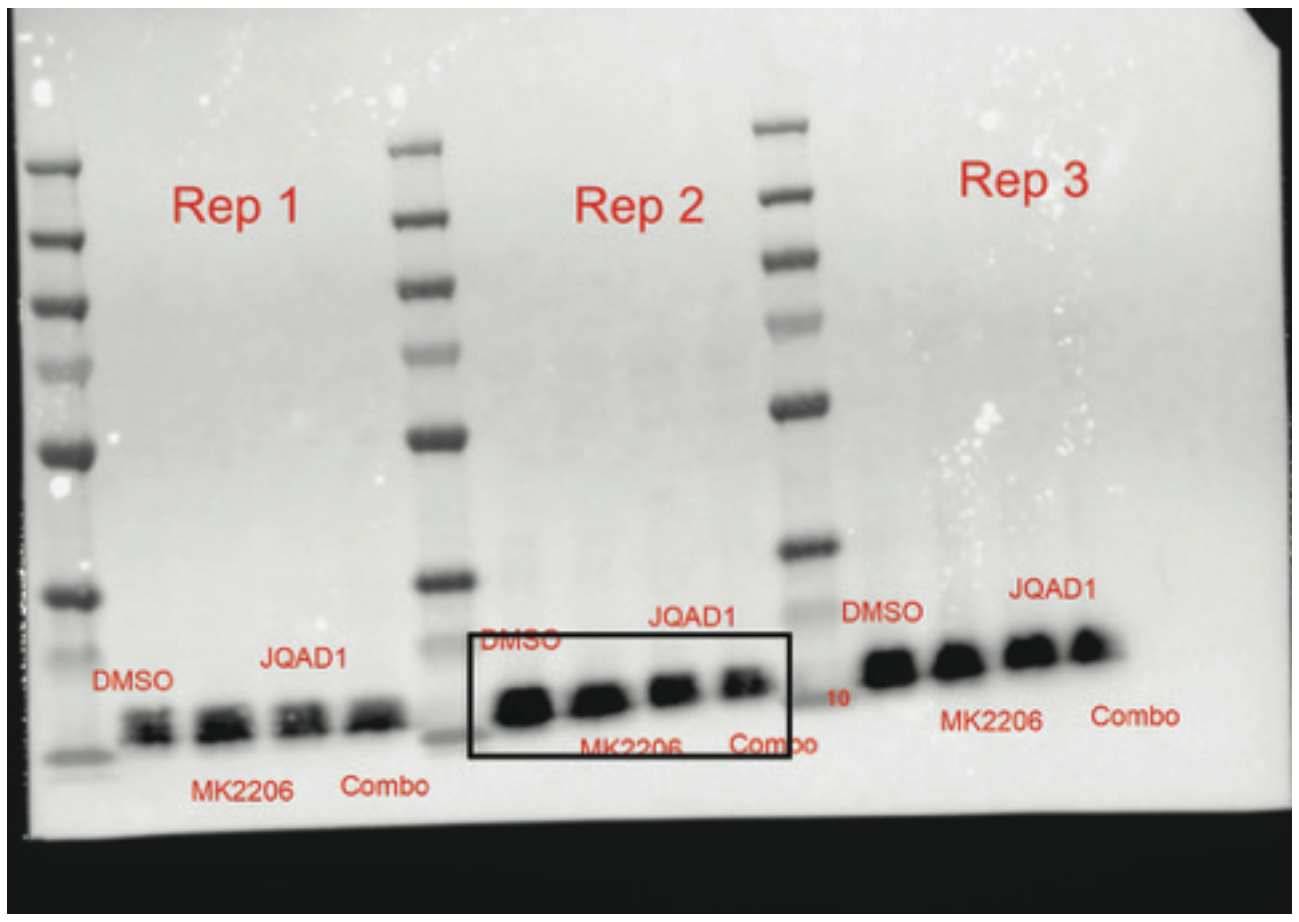

4EBP1

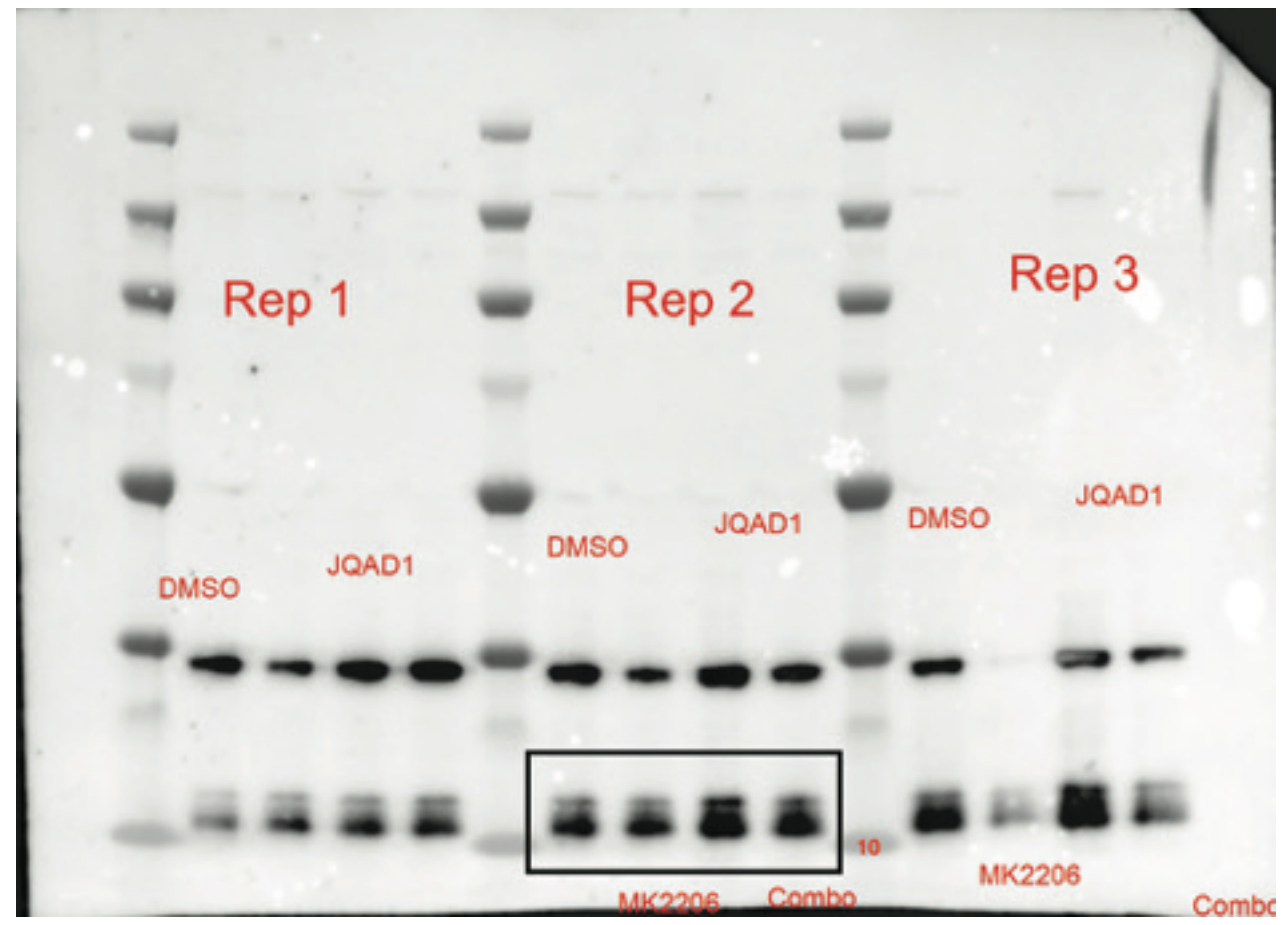

pEIF4E

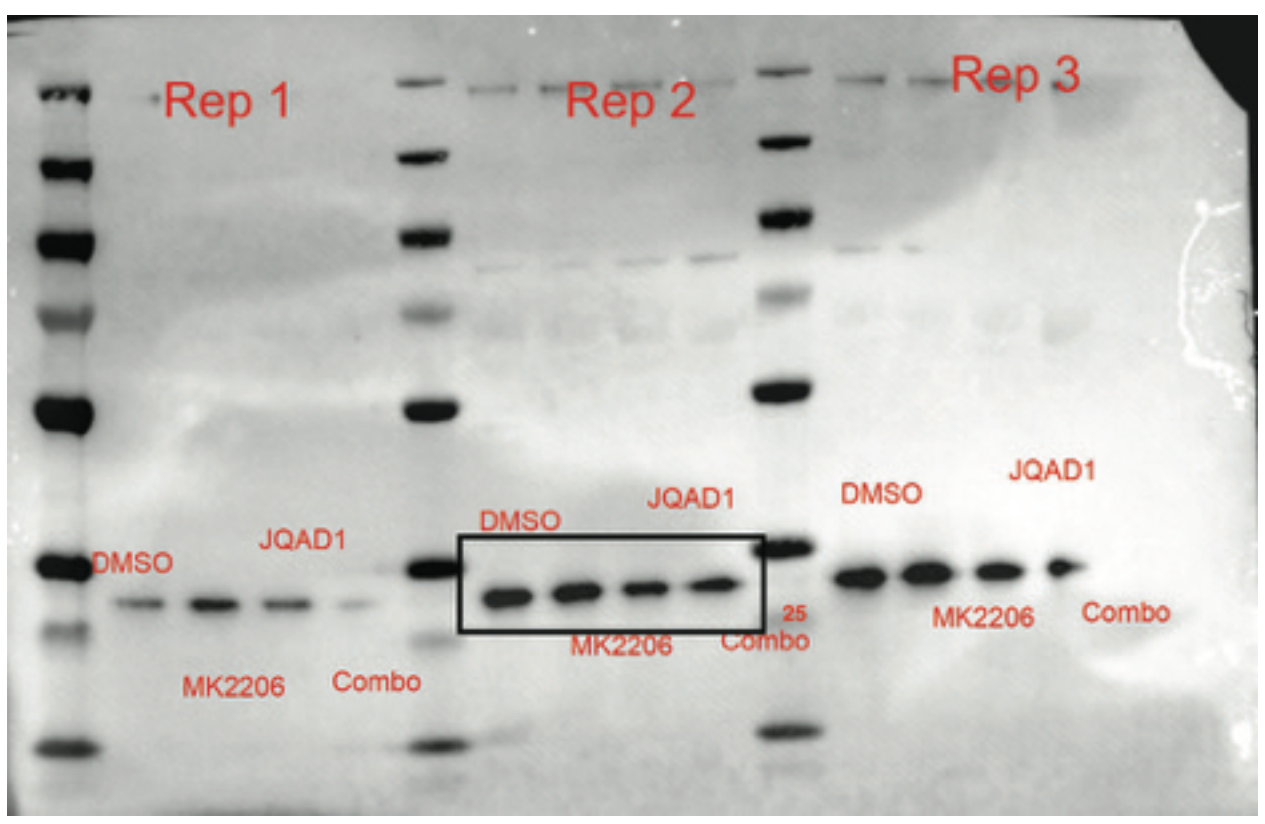

EIF4E

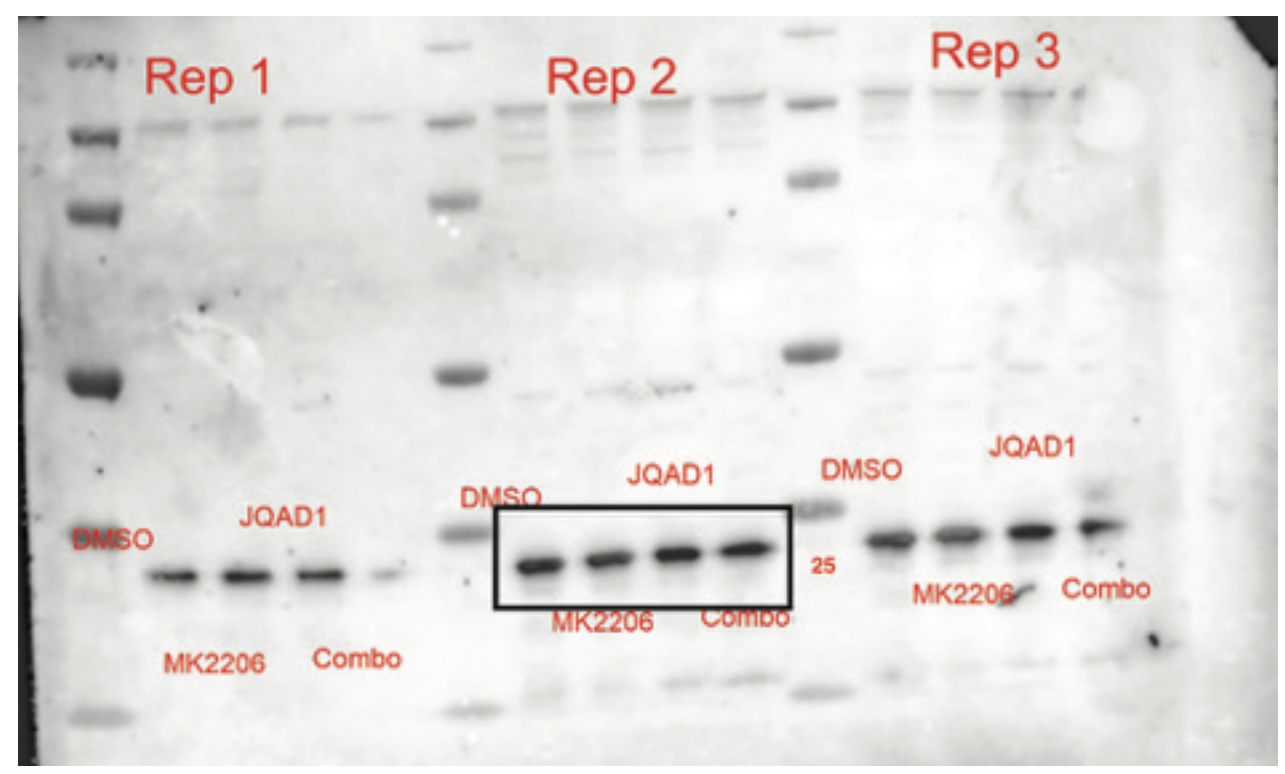

pS235/236-S6

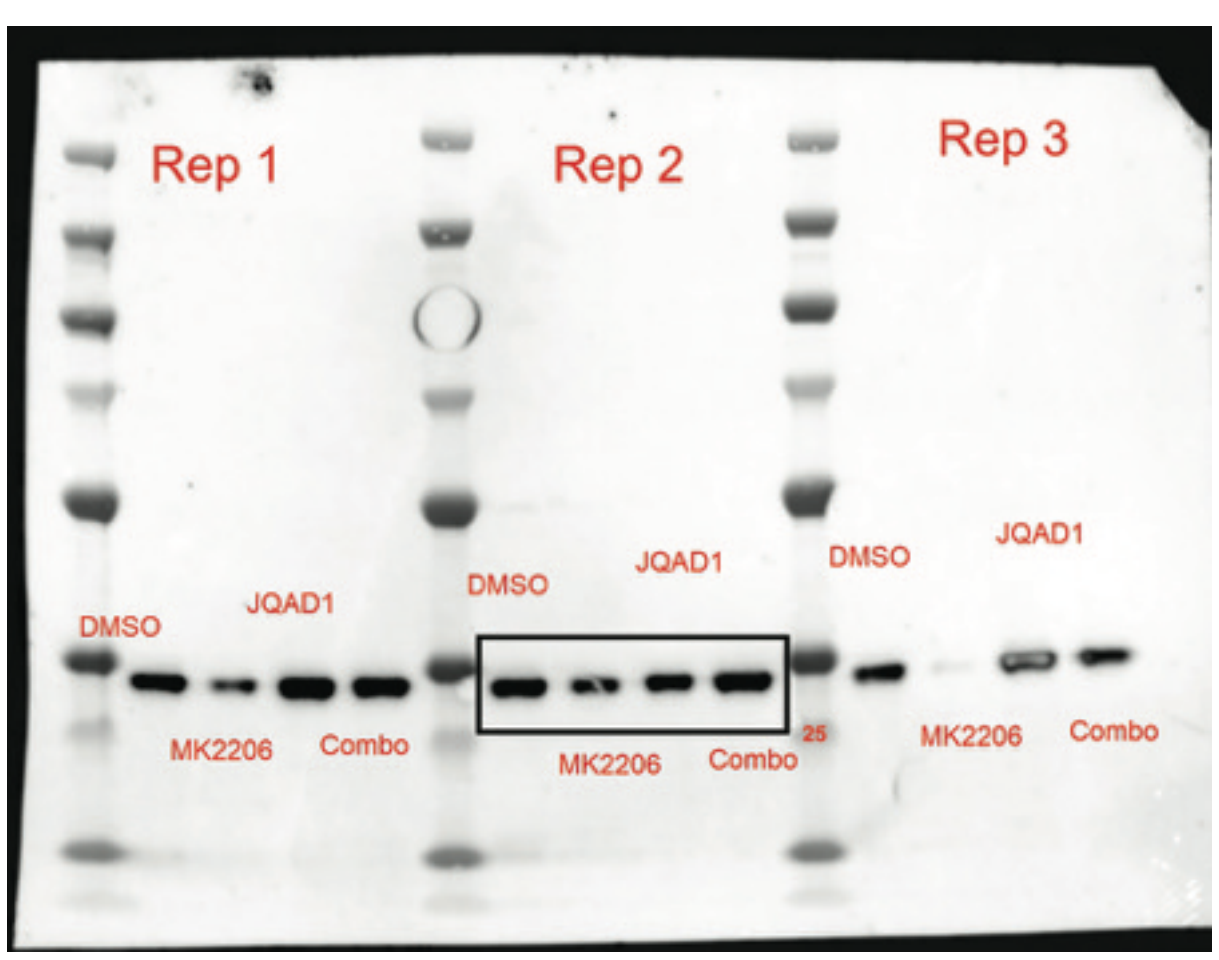

S6

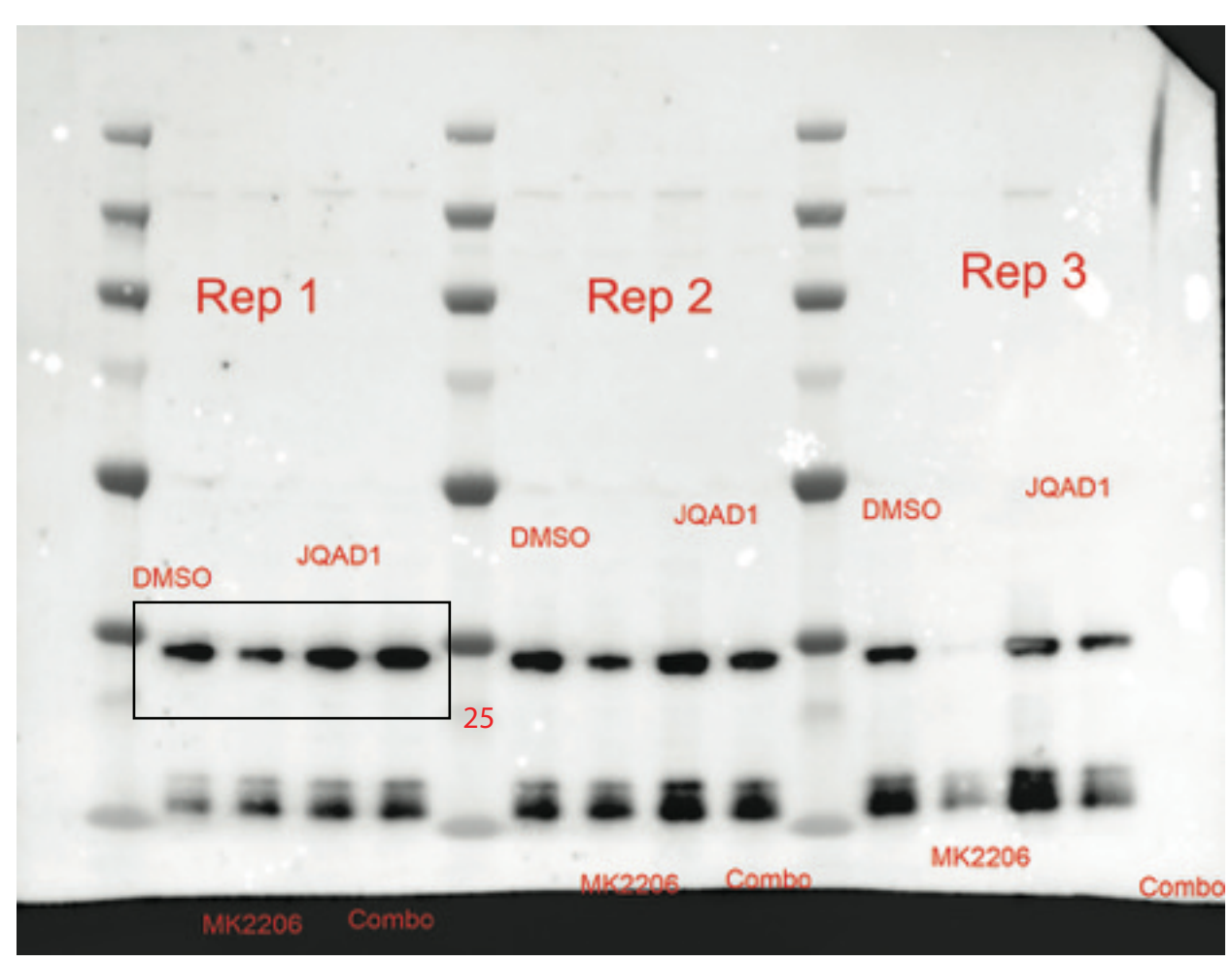

Supplement: Supplementary file 5 [file jem_20251898_sourcedatafs4.pdf]
